# Controllable Synthesis of Thioacetals/Thioketals and β-Sulfanyl Ketones Mediated by Methanesulfonic Anhydride and Sulfuric Acid Sulfuric Acid from Aldehyde/Acetone and Thiols

**DOI:** 10.3390/molecules29204785

**Published:** 2024-10-10

**Authors:** Hexia Ye, Xinyao Zhao, Yajie Fu, Haibo Liu, Junchen Li, Xiaojing Bi

**Affiliations:** 1 State Key Laboratory of NBC Protection for Civilian, Beijing 102205, China; yehexia6688@yeah.net (H.Y.); xy01253@yeah.net (X.Z.); yajief2022@163.com (Y.F.); hbbnu@126.com (H.L.); 2 School of Chemistry and Environmental Engineering, Sichuan University of Science &Engineering, 180 Xueyuan Street, Huixing Lu, Zigong 643000, China

**Keywords:** thia-Michael addition, thioacetal, thioketal, methanesulfonic anhydride, H_2_SO_4_

## Abstract

A novel and controllable synthesis of thioacetals/thioketals and β-sulfanyl ketones mediated by the reaction of aldehyde/acetone with thiols has been developed. In this protocol, β-sulfanyl ketones can be generated without the prior preparation of α, β-unsaturated carbonyl compounds. A variety of thiols reacted with aldehyde/acetone and provided the corresponding thioacetals/thioketals and β-sulfanyl ketones in good to excellent yields, respectively. This protocol is operationally simple, mild, and atom-economical, providing controllable access to thioacetals/thioketals and thia-Michael addition products under mild conditions.

## 1. Introduction

**Figure 1:**
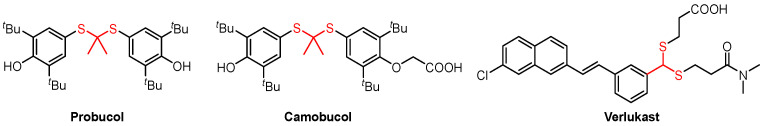
Examples of applications of thioacetals/thioketals.

Sulfides have garnered significant attention from scientists due to their diverse chemical valence states and the resulting rich chemical structure and stereo conformation. Thioether, sulfoxide, sulfone, and sulfonamide are widely utilized pharmacophores in commercially available pharmaceuticals [1]. Furthermore, owing to the high reactivity of sulfides, they are frequently employed as a key intermediate in the total synthesis of natural products [2] and various synthetic reactions [3]. Thioacetals and thioketals, among a wide variety of sulfur-containing compounds, are commonly utilized in organic synthesis as precursors for fluorination and alkylation and for olefin formation [4,5,6]. Furthermore, thioketals exhibit diverse biological activities and are employed as drugs with numerous pharmacological effects (Figure 1) [7,8,9]. The construction of C-S bonds through the Michael addition reaction between mercaptan with α, β-unsaturated carbonyl compounds holds significant value in both chemistry and biology [10].

**Figure 2:**
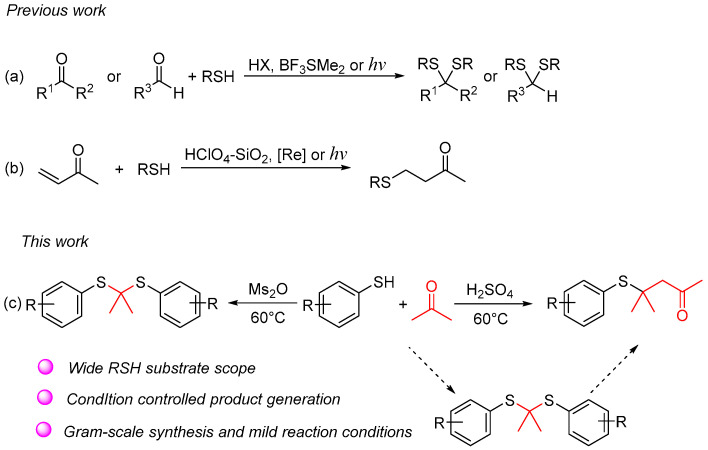
Overview of synthetic strategies to access thioacetals/thioketals and β-sulfanyl ketones. (**a**) The synthesis strategies of thioacetals and thioketals; (**b**) The synthesis strategies of β-sulfanyl ketones; (**c**) Our work.

Thioacetals and thioketals are typically synthesized through the condensation of carbonyl compounds with thiols or dithiols, employing protic acids, Lewis acids, or photocatalysis (Figure 2a) [11,12,13,14,15,16,17,18]. The addition of thiol to aryl and alkyl substituted α, β-unsaturated carbonyl compounds represents the primary method for synthesizing β-sulfanyl ketones (Figure 2b) [19,20,21]. Herein, we investigated a novel and controllable synthesis of thioacetals/thioketals and β-sulfanyl ketones from acetone and thiols. With this synthetic method, β-sulfanyl ketones can be formed without preparation of α, β-unsaturated carbonyl compounds in advance (Figure 2c).

## 2. Results and Discussion

The Michael addition reaction has been extensively studied due to its high application value. In the previous investigations, we discovered that mercaptan can be transformed into a thia-Michael addition product when mediated by p-toluene sulfonic anhydride in acetone as the solvent. Thioketals and α, β-unsaturated ketone were also monitored during the reaction (Table 1). According to preliminary results, a multitude of experiments were conducted to optimize the reaction conditions.

We initiated our investigation using 4-methylbenzenethiol (**1a**) and acetone (**2**) as the model substrates and then allowed them to react at 60 °C for 24 h (Table 1, entry 1). Initially, various additives (Ts_2_O, Ms_2_O, TsOH, HCl, and H_2_SO_4_) were added to the model reaction to identify suitable accelerators. Both Ts_2_O and TsOH exhibited comparable facilitation abilities in this reaction (Table 1, entries 1 and 3). Compared to Ts_2_O, the addition of TsOH makes the reaction proceed more completely (Table 1, entry 3). There are no intermediates remaining after 24 h. Notably, it was observed that the addition of Ms_2_O mainly generates thioketal for an extended period, with only a limited number of products being generated over time.

Inorganic acids can also promote the reaction, and sulfuric acid displayed superior efficacy among them (Table 1, entry 5). This enhancement may be attributed to the high concentration of hydrogen ions dissociated from sulfuric acid relative to other acids used in equivalent amounts. Decreasing the amount of acetone provides better results (Table 1, entry 6), whereas reducing the amount of H_2_SO_4_ is not conducive to the reaction (Table 1, entries 7–8). Enhanced yields were achieved using DCE, DCM, and toluene as solvents (Table 1, entries 6, 9–10), while polar solvents, such as water and acetonitrile, hindered progress (Table 1, entries 11–14). Neither lower nor higher temperatures provided optimal outcomes for this process (Table 1, entries 15–18). According to the above experiments, we established optimal reaction conditions, which yielded product **4a** with an isolated yield of 86% (Table 1, entry 6).

With the optimized reaction conditions established, we investigated the scope and generality of thiol substrates in conjunction with ketones (Table 2). Overall, both electron-donating and electron-withdrawing substituted substrates provided the desired products in good yields. A diverse array of functional groups, such as Me, *i*-Pr, OMe, F, Cl, Br, I, CF_3_, NO_2_, and COOMe, were compatible with this reaction and provided satisfactory product yields (Table 2, **4a**–**4m**). Substituted benzyl mercaptan and naphthalene-2-thiol were well-tolerated under the optimal reaction conditions (Table 2, **4n**–**4q**, **4t**). However, alkyl mercaptans and heteroaryl mercaptans afforded the corresponding products with lower yields (Table 2, **4r**–**4s**, **4u**). Motivated by the above results, we further explored the substrate scope of ketones, such as acetophenone and 2-pentanone. Unfortunately, no desired products were obtained due to the steric hindrance.

In the process of condition optimization, it was observed that when methanesulfonic anhydride was introduced into the reaction mixture, thioketal emerged as the main product, rather than β-sulfanyl ketone (Table 1, entry 2). The results prompted us to carry out an extended study of the reaction with methanesulfonic anhydride as the accelerator (Table 3). It was found that the thiophenol and benzyl mercaptan substituted with Me or OMe can obtain the target substances in medium to good yields (Table 3, **3a**–**3g**), while thiophenol substituted with other substituents (NO_2_, Cl, Br, etc.) has a complex reaction system, and very little thioketal is generated, which is difficult to be separated and purified.

In addition to acetone, the applicability of aromatic aldehydes as substrates was also discussed. The results indicated that the target product could be obtained with medium to good yield from different substituted aromatic aldehydes (Table 4). Notably, even thiophenol substituted with NO_2_ achieved a yield of approximately 70% for the target material (Table 4, **3′o**).

**Figure 3:**
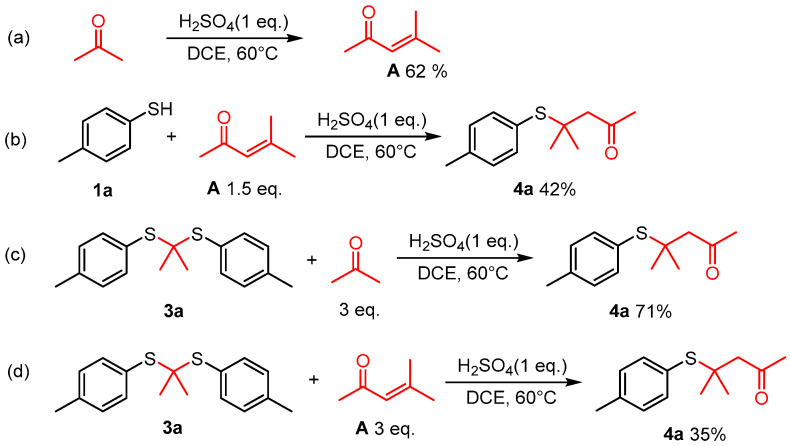
Control experiments. (**a**) Acetone converted into α, β-unsaturated ketone in the absence of 4-methylbenzenethiol under optimal reaction conditions; (**b**) 4-Methylbenzenethiol reacted with **A** to form **4a**; (**c**) Thioketal **3a** reacted with 3 eq acetone under optimal reaction conditions to produce **4a** in a yield of 71%; (**d**) Thioketal **3a** reacted with **A** to produce the target compound **4a** with a yield of 35%.

During the reaction process, α, β-unsaturated ketone and thioketal were identified alongside substrates and desired products at various stages of the reaction. In order to clarify the reaction mechanism, several control experiments were performed. In the absence of 4-methylbenzenethiol, acetone was converted into α, β-unsaturated ketone **A** under current reaction conditions (Figure 3a). When **A** was introduced into the reaction instead of acetone, a yield of 42% for product **4a** was obtained (Figure 3b). Thioketal reacted with acetone under the optimal conditions to produce **4a** in a yield of 71% (Figure 3c). Additionally, with thioketals **3a** and **A** as substrates, only 35% of **4a** was obtained (Figure 3d). The reaction process was monitored via GC-MS, and it was surprising that at the initial stage of the reaction, only thioketal was detected in the reaction mixture without a product or α, β-unsaturated ketone **A**. The presence of compound **A** became evident in later stages. Thus, we assume that the reaction (b), (c), and (d) exist at the same time, and reaction (c) is the main process (Figure 3).

**Figure 4:**
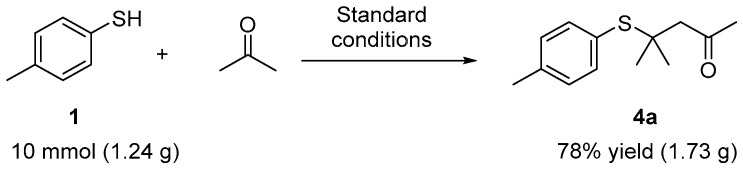
Gram-scale experiment.

To demonstrate the practicality of the present method, a gram-scale experiment was performed under the standard reaction conditions, yielding the product **4a** in a 78% yield (Figure 4). The gram-scale experiment shows the promising application of this method.

**Figure 5:**
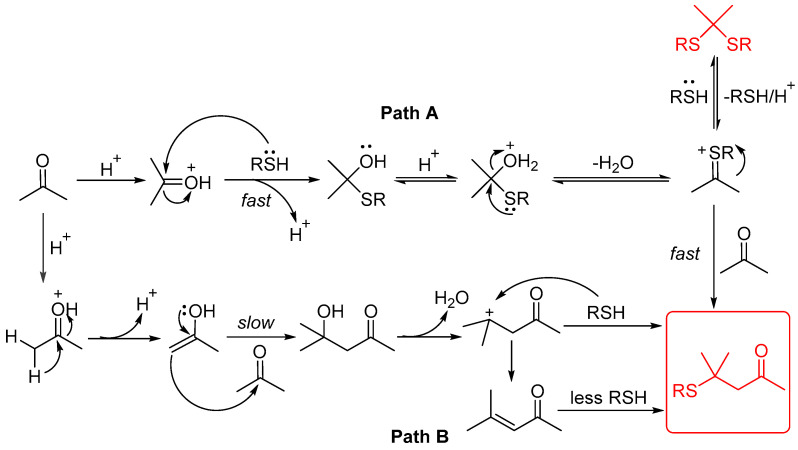
Plausible mechanism.

Based on the above results and the relevant literature [22,23,24,25], a possible mechanism was proposed, as shown in Figure 5. The reaction products (**4**) are generated more rapidly via Path A. Thioketals are formed uniformly and quickly, which is reversible under acidic conditions, leading to positively charged sulfur species that subsequently react with acetone to generate the final products. In contrast, only a minimal quantity of products is produced through Path B. The α, β-unsaturated ketone **A** was detected later using GC-MS. The conversion of acetone into diketone alcohol occurs at a slow rate. Diacetone alcohol eliminates one molecule of H_2_O and directly reacts with thiols to produce the final products. By comparing these two reaction mechanisms, we propose that β-sulfanyl ketones are preferentially formed through reactions with highly reactive charged sulfur species (Path A).

## 3. Materials and Methods

General Information.

All reactions were performed in a sealed tube with magnetic stirring. Unless otherwise stated, all commercially available reagents (innochem, Beijing, China) were used without further purification. Reactions were monitored using thin-layer chromatography (TLC), GC/MS, or LC/MS. NMR spectra were recorded on Bruker DRX-300 instruments (Bruker, Rheinstetten, Germany) and were calibrated using residual undeuterated solvent (CHCl_3_ at 7.26 ppm for ^1^H NMR and 77.16 ppm for ^13^C NMR). Data were reported as follows: chemical shift, multiplicity (s = singlet, d = doublet, t = triplet, q = quartet, dd = doublet of doublets, td = triplet of doublets, qd = quartet of doublets, m = multiplet), coupling constants (Hz) and integration. High-resolution mass spectra (HRMS) were recorded on an Agilent LC/Xevo G_2_-XS QTOF mass spectrometer (Agilent, Palo Alto, CA, USA) using electrospray ionization time of flight reflectron experiments.

Experimental Procedure.

A 10 mL sealed tube was charged with substituted various thiols (0.6 mmol), methanesulfonic anhydride (Ms_2_O, 0.6 mmol), acetone (1 mL), and DCE (5 mL). The resulting solution was stirred at 60 °C and monitored using TLC until the reaction was complete. Saturated aqueous Na_2_CO_3_ solution (10 mL) and EtOAc (10 mL) was added to the mixture. The layers were separated, and the aqueous layer was washed with EtOAc (2 × 10 mL). Combined organic layers were washed with brine (10 mL), dried with anhydrous Na_2_SO_4_, filtered, and concentrated. The residue was purified using column chromatography on silica gel, with hexane/ethyl acetate = 20:1 as the eluent to provide the desired products **3**.

A 10 mL sealed tube was charged with thiols (0.6 mmol), methanesulfonic anhydride (Ms_2_O, 0.6 mmol), **2′** (0.9 mmol), and DCE (5 mL). The resulting solution was stirred at 60 °C and monitored by TLC until the reaction was complete. Saturated aqueous Na_2_CO_3_ solution (10 mL) and EtOAc (10 mL) were added to the resulting mixture. The layers were separated, and the aqueous layer was washed with EtOAc (2 × 10 mL). The combined organic layers were washed with brine (10 mL), dried with anhydrous Na_2_SO_4_, filtered, and concentrated. The residue was purified using column chromatography on silica gel, with hexane/ethyl acetate = 50:1 as the eluent to provide the desired products **3′**.

A 10 mL sealed tube was charged with thiols (0.6 mmol), H_2_SO_4_ (0.6 mmol), acetone (1 mL), and DCE (5 mL). The resulting solution was stirred at 60 °C and monitored using TLC until the reaction was complete. Saturated aqueous Na_2_CO_3_ solution (10 mL) and EtOAc (10 mL) were added to the resulting mixture. The layers were separated, and the aqueous layer was washed with EtOAc (2 × 10 mL). The combined organic layers were washed with brine (10 mL), dried with anhydrous Na_2_SO_4_, filtered, and concentrated. The residue was purified using column chromatography on silica gel, with hexane/ethyl acetate = 10:1 as the eluent to provide the desired products **4**.

The gram-scale experiment procedure. A 250 mL round-bottomed flask was charged with 4-methylbenzenethiol (**1a**) (10 mmol,1.2432 g), H_2_SO_4_ (10 mmol), acetone (16 mL), and DCE (84 mL). The bottle was sealed with a rubber stopper, and the resulting solution was stirred at 60 °C for 24 h. Saturated aqueous Na_2_CO_3_ solution (100 mL) and EtOAc (100 mL) were added to the resulting mixture. The layers were separated, and the aqueous layer was washed with EtOAc (2 × 100 mL). The combined organic layers were washed with brine (100 mL), dried with anhydrous Na_2_SO_4_, filtered, and concentrated. The residue was purified using column chromatography on silica gel, with hexane/ethyl acetate = 10:1 as the eluent to provide the desired product **4a**. Characterization data for the products can be found in the Supplementary Materials, along with references of previous reports which provide support for the identities of the products [20,26,27,28,29,30,31].

Propane-2,2-diylbis(*p*-tolylsulfane) (**3a**) [26]. White solid, 76% yield. ^1^H NMR (300 MHz, CDCl_3_) δ 7.53 (d, *J* = 8.0 Hz, 4H), 7.16 (d, *J* = 7.9 Hz, 4H), 2.37 (s, 6H), 1.49 (s, 6H). ^13^C NMR (75 MHz, CDCl_3_) δ 139.37, 137.16, 129.48, 128.92, 59.24, 30.77, 21.43.

Propane-2,2-diylbis(*m*-tolylsulfane) (**3b**). White solid, 61% yield. ^1^H NMR (300 MHz, Chloroform-d) δ 7.45 (d, J = 6.5 Hz, 4H), 7.20 (dd, J = 12.1, 7.7 Hz, 4H), 2.35 (s, 6H), 1.51 (s, 6H). ^13^C NMR (75 MHz, CDCl_3_) δ 138.35, 137.66, 134.08, 132.14, 129.98, 128.44, 59.34, 30.99, 21.42.

Propane-2,2-diylbis((4-methoxyphenyl)sulfane) (**3c**). White solid, 67% yield. ^1^H NMR (300 MHz, CDCl_3_) δ 7.54 (d, *J* = 8.6 Hz, 4H), 6.88 (d, *J* = 8.7 Hz, 4H), 3.82 (s, 6H), 1.45 (s, 6H). ^13^C NMR (75 MHz, CDCl_3_) δ = 160.62, 138.82, 123.28, 114.14, 59.33, 55.41, 30.52.

Propane-2,2-diylbis((4-isopropylphenyl)sulfane) (**3d**). White solid, 45% yield. ^1^H NMR (300 MHz, Chloroform-*d*) δ 7.56 (d, *J* = 8.1 Hz, 4H), 7.21 (d, *J* = 8.0 Hz, 4H), 2.93 (hept, *J* = 6.8 Hz, 2H), 1.50 (s, 6H), 1.26 (d, *J* = 6.9 Hz, 12H). ^13^C NMR (75 MHz, CDCl_3_) δ 150.14, 137.19, 129.31, 126.83, 59.51, 34.02, 30.88, 24.01.

Propane-2,2-diylbis(benzylsulfane) (**3e**). White solid, 66% yield. ^1^H NMR (300 MHz, CDCl_3_) δ 7.34 (dt, *J* = 14.4, 7.5 Hz, 10H), 3.93 (s, 4H), 1.67 (s, 6H). ^13^C NMR (75 MHz, CDCl_3_) δ 137.87, 129.23, 128.62, 127.03, 57.30, 35.19, 30.85.

Propane-2,2-diylbis((4-methylbenzyl)sulfane) (**3f**). White solid, 63% yield. ^1^H NMR (300 MHz, CDCl_3_) δ 7.45 (d, *J* = 7.8 Hz, 4H), 7.33 (d, *J* = 7.8 Hz, 4H), 4.07 (s, 4H), 2.54 (s, 6H), 1.83 (s, 6H). ^13^C NMR (75 MHz, CDCl_3_) δ 136.62, 134.72, 129.31, 129.12, 57.18, 34.86, 30.84, 21.22.

Propane-2,2-diylbis((4-methoxybenzyl)sulfane) (**3g**). White solid, 67% yield. ^1^H NMR (300 MHz, CDCl_3_) δ 7.37 (d, *J* = 8.5 Hz, 4H), 6.95 (d, *J* = 8.5 Hz, 4H), 3.95 (s, 4H), 3.89 (s, 6H), 1.72 (s, 6H). ^13^C NMR (75 MHz, CDCl_3_) δ 158.66, 130.28, 129.71, 114.03, 57.04, 55.36, 34.52, 30.85.

(Phenylmethylene)bis(*p*-tolylsulfane) (**3′a**) [27]. White solid, 79% yield. ^1^H NMR (300 MHz, CDCl_3_) δ 7.30 (dd, *J* = 7.6, 1.7 Hz, 2H), 7.25–7.20 (m, 7H), 7.02 (d, *J* = 7.9 Hz, 4H), 5.29 (s, 1H), 2.27 (s, 6H). ^13^C NMR (75 MHz, CDCl_3_) δ 140.08, 138.13, 133.25, 130.95, 129.69, 128.48, 128.00, 61.39, 21.31.

(*p*-Tolylmethylene)bis(*p*-tolylsulfane) (**3′b**). White solid, 75% yield. ^1^H NMR (300 MHz, CDCl_3_) δ 7.35 (d, *J* = 8.0 Hz, 6H), 7.16 (t, *J* = 7.9 Hz, 6H), 5.43 (s, 1H), 2.42 (s, 3H), 2.40 (s, 6H). ^13^C NMR (75 MHz, CDCl_3_) δ 137.91, 137.69, 137.14, 133.03, 131.29, 129.64, 129.17, 127.85, 61.18, 21.25.

(*m*-Tolylmethylene)bis(*p*-tolylsulfane) (**3′c**). White solid, 74% yield. ^1^H NMR (300 MHz, CDCl_3_) δ 7.29 (d, *J* = 8.1 Hz, 4H), 7.22–7.15 (m, 3H), 7.09 (d, *J* = 7.9 Hz, 5H), 5.34 (s, 1H), 2.34 (s,9H). ^13^C NMR (75 MHz, CDCl_3_) δ 139.91, 138.13, 138.00, 133.12, 131.10, 129.63, 128.78, 128.56, 128.31, 125.00, 61.44, 21.47, 21.26.

((3, 5-Dimethylphenyl)methylene)bis(*p*-tolylsulfane) (**3′d**). White solid, 77% yield. ^1^H NMR (300 MHz, CDCl_3_) δ 7.29 (d, *J* = 8.1 Hz, 4H), 7.09 (d, *J* = 7.9 Hz, 4H), 7.00 (s, 2H), 6.91 (s, 1H), 5.31 (s, 1H), 2.34 (s, 6H), 2.30 (s, 6H). ^13^C NMR (75 MHz, CDCl_3_) δ 139.82, 137.98, 137.92, 133.05, 131.27, 129.73, 129.61, 125.64, 61.54, 21.35, 21.26.

((4-Ethylphenyl)methylene)bis(*p*-tolylsulfane) (**3′e**). White solid, 76% yield. ^1^H NMR (300 MHz, CDCl_3_) δ 7.31 (t, *J* = 8.5 Hz, 6H), 7.12 (dd, *J* = 16.9, 8.0 Hz, 6H), 5.38 (s, 1H), 2.66 (q, *J* = 7.6 Hz, 2H), 2.34 (s, 6H), 1.26 (t, *J* = 7.6 Hz, 3H). ^13^C NMR (75 MHz, CDCl_3_) δ 144.03, 137.89, 137.24, 132.99, 131.21, 129.62, 127.96, 127.84, 61.17, 28.61, 21.25, 15.52.

((4-Methoxyphenyl)methylene)bis(*p*-tolylsulfane) (**3′f**). White solid, 74% yield. ^1^H NMR (300 MHz, CDCl_3_) δ 7.24–7.20 (m, 6H), 7.03 (d, *J* = 7.9 Hz, 4H), 6.77 (d, *J* = 8.7 Hz, 2H), 5.29 (s, 1H), 3.77 (s, 3H), 2.29 (s, 6H). ^13^C NMR (75 MHz, CDCl_3_) δ 159.27, 138.02, 133.12, 132.14, 131.15, 129.68, 129.22, 113.84, 60.70, 55.40, 21.31.

((4-Fluorophenyl)methylene)bis(*p*-tolylsulfane) (**3′g**). White solid, 87% yield. ^1^H NMR (300 MHz, CDCl_3_) δ 7.39–7.27 (m, 6H), 7.10 (d, *J* = 8.1 Hz, 4H), 6.97 (t, *J* = 8.6 Hz, 2H), 5.38 (s, 1H), 2.35 (s, 6H). ^13^C NMR (75 MHz, CDCl_3_) δ 162.20 (d, *J* = 246.9 Hz), 138.27, 135.85 (d, *J* = 3.2 Hz), 133.35, 130.53, 129.72, 129.61, 115.28 (d, *J* = 21.7 Hz), 60.42, 21.25. ^19^F NMR (282 MHz, CDCl_3_) δ -113.83 (tt, *J* = 8.6, 5.3 Hz).

((4-Chlorophenyl)methylene)bis(*p*-tolylsulfane) (**3′h**). White solid, 88% yield. ^1^H NMR (300 MHz, CDCl_3_) δ 7.36–7.27 (m, 8H), 7.12 (d, *J* = 7.9 Hz, 4H), 5.37 (s, 1H), 2.37 (s, 6H). ^13^C NMR (75 MHz, CDCl_3_) δ 138.62, 138.32, 133.51, 133.36, 130.38, 129.74, 129.31, 128.53, 60.51, 21.25.

((4-Bromophenyl)methylene)bis(*p*-tolylsulfane) (**3′i**). White solid, 87% yield. ^1^H NMR (300 MHz, CDCl_3_) δ 7.41 (d, *J* = 8.5 Hz, 2H), 7.26 (m, 6H), 7.10 (d, *J* = 8.0 Hz, 4H), 5.33 (s, 1H), 2.35 (s, 6H). ^13^C NMR (75 MHz, CDCl_3_) δ 139.14, 138.34, 133.36, 131.49, 130.34, 129.75, 129.63, 121.72, 60.58, 21.28.

4-(Bis(*p*-tolylthio)methyl)benzonitrile (**3′j**). White solid, 86% yield. ^1^H NMR (300 MHz, CDCl_3_) δ 7.44 (d, *J* = 8.2 Hz, 2H), 7.31 (d, *J* = 8.2 Hz, 2H), 7.18 (d, *J* = 8.0 Hz, 4H), 7.01 (d, *J* = 8.0 Hz, 4H), 5.27 (s, 1H), 2.25 (s, 6H). ^13^C NMR (75 MHz, CDCl_3_) δ 145.29, 138.64, 133.54, 132.02, 129.74, 129.50, 128.56, 118.57, 111.30, 60.55, 21.15.

4-(Bis(*p*-tolylthio)methyl)benzaldehyde (**3′k**). White solid, 86% yield. ^1^H NMR (300 MHz, CDCl_3_) δ 9.70 (s, 1H), 7.51 (d, *J* = 8.1 Hz, 2H), 7.21 (d, *J* = 8.1 Hz, 2H), 7.01 (d, *J* = 8.0 Hz, 4H), 6.81 (d, *J* = 7.9 Hz, 4H), 5.12 (s, 1H), 2.06 (s, 6H). ^13^C NMR (75 MHz, CDCl_3_) δ 191.66, 146.82, 138.53, 135.66, 133.51, 129.87, 129.74, 128.54, 60.90, 21.19.

((4-Chlorophenyl)methylene)bis((4-methoxyphenyl)sulfane) (**3′l**). White solid, 72% yield. ^1^H NMR (300 MHz, CDCl_3_) δ 7.73–7.66 (m, 4H), 7.65–7.53 (m, 4H), 7.23–7.17 (m, 4H), 5.54 (s, 1H), 4.19 (s, 6H). ^13^C NMR (75 MHz, CDCl_3_) δ 160.16, 138.83, 136.15, 133.45, 129.34, 128.50, 124.33, 114.50, 62.11, 55.39.

Dimethyl 4,4’-((phenylmethylene)bis(sulfanediyl))dibenzoate (**3′m**). White solid, 75% yield. ^1^H NMR (300 MHz, CDCl_3_) δ 7.89 (d, *J* = 8.5 Hz, 4H), 7.46 (dd, *J* = 7.7, 1.5 Hz, 2H), 7.35 (d, *J* = 8.5 Hz, 4H), 7.29 (d, *J* = 7.4 Hz, 2H), 5.69 (s, 1H), 3.87 (s, 6H). ^13^C NMR (75 MHz, CDCl_3_) δ 166.49, 140.94, 138.13, 130.01, 129.97, 128.85, 128.71, 128.66, 127.93, 57.59, 52.18.

4-(Bis((4-bromophenyl)thio)methyl)benzonitrile (**3′n**). White solid, 75% yield. ^1^H NMR (300 MHz, CDCl_3_) δ 7.55 (d, *J* = 8.3 Hz, 2H), 7.38 (d, *J* = 8.4 Hz, 6H), 7.17 (dd, *J* = 8.8, 2.1 Hz, 4H), 5.33 (s, 1H). ^13^C NMR (75 MHz, CDCl_3_) δ 144.25, 134.79, 132.43, 132.30, 131.97, 128.59, 123.20, 118.39, 112.07, 60.03.

(*p*-tolylmethylene)bis((4-nitrophenyl)sulfane) (**3′o**). White solid, 70% yield. ^1^H NMR (300 MHz, CDCl_3_) δ 8.08 (d, *J* = 8.8 Hz, 4H), 7.42 (dd, *J* = 8.3, 6.5 Hz, 6H), 7.17 (d, *J* = 7.9 Hz, 2H), 5.80 (s, 1H), 2.34 (s, 3H). ^13^C NMR (75 MHz, CDCl_3_) δ 146.34, 144.20, 139.42, 133.67, 129.97, 129.36, 127.88, 124.05, 56.18, 21.29.

(*p*-tolylmethylene)bis(naphthalen-2-ylsulfane) (**3′p**). White solid, 71% yield. ^1^H NMR (300 MHz, CDCl_3_) δ 7.86 (s, 2H), 7.78 (dd, *J* = 5.8, 3.5 Hz, 2H), 7.74–7.65 (m, 4H), 7.52–7.34 (m, 8H), 7.12 (d, *J* = 7.9 Hz, 2H), 5.69 (s, 1H), 2.34 (s, 3H). ^13^C NMR (75 MHz, CDCl_3_) δ 138.11, 136.69, 133.61, 132.62, 132.25, 131.51, 129.57, 129.42, 128.44, 127.90, 127.77, 127.69, 126.54, 126.43, 60.34, 21.31, 1.16.

(Phenylmethylene)bis(benzylsulfane) (**3′q**) [28]. White solid, 73% yield. ^1^H NMR (300 MHz, CDCl_3_) δ 7.32–7.10 (m, 11H), 7.09–7.00 (m, 4H), 4.38 (s, 1H), 3.68 (d, *J* = 13.4 Hz, 2H), 3.45 (d, *J* = 13.4 Hz, 2H). ^13^C NMR (75 MHz, CDCl_3_) δ 139.64, 137.81, 129.05, 128.68, 128.57, 128.09, 128.03, 127.07, 50.96, 36.61.

4-Methyl-4-(*p*-tolylthio)pentan-2-one (**4a**) [20]. Oil liquid, 86% yield. ^1^H NMR (300 MHz, CDCl_3_) δ 7.39 (d, *J* = 8.0 Hz, 2H), 7.14 (d, *J* = 7.9 Hz, 2H), 2.64 (s, 2H), 2.34 (s, 3H), 2.13 (s, 3H), 1.36 (s, 6H). ^13^C NMR (75 MHz, CDCl_3_) δ 206.75, 139.31, 137.62, 129.54, 127.92, 54.41, 46.91, 32.26, 28.13, 21.30. HRMS (ESI-MS) [M + Na]^+^: found 245.0985; calculated for C_13_H_18_NaOS^+^: 245.0971.

4-Methyl-4-(*m*-tolylthio)pentan-2-one (**4b**) [29]. Oil liquid, 84% yield. ^1^H NMR (300 MHz, CDCl_3_) δ 7.38–7.26 (m, 2H), 7.20 (m, *J* = 17.9, 7.3 Hz, 2H), 2.65 (s, 2H), 2.34 (s, 3H), 2.13 (s, 3H), 1.38 (s, 6H). ^13^C NMR (75 MHz, CDCl_3_) δ 206.66, 138.39, 138.22, 134.61, 131.12, 129.91, 128.47, 54.43, 46.94, 32.21, 28.23, 21.27. HRMS (ESI-MS) [M + Na]^+^: found 245.0982; calculated for C_13_H_18_NaOS^+^: 245.0971.

4-Methyl-4-(*o*-tolylthio)pentan-2-one (**4c**) [29]. Oil liquid, 78% yield. ^1^H NMR (300 MHz, CDCl_3_) δ 7.54 (d, *J* = 7.6 Hz, 1H), 7.34–7.25 (m, 2H), 7.23–7.13 (m, 1H), 2.75 (s, 2H), 2.54 (s, 3H), 2.17 (s, 3H), 1.42 (s, 6H). ^13^C NMR (75 MHz, CDCl3) δ 206.66, 144.03, 138.99, 130.99, 130.66, 129.32, 125.97, 54.63, 48.56, 32.27, 28.22, 21.87. HRMS (ESI-MS) [M + Na]^+^: found 245.0984; calculated for C_13_H_18_NaOS^+^: 245.0971.

4-((4-Isopropylphenyl)thio)-4-methylpentan-2-one (**4d**). Oil liquid, 77% yield. ^1^H NMR (300 MHz, CDCl_3_) δ 7.42 (d, *J* = 8.1 Hz, 2H), 7.18 (d, *J* = 8.1 Hz, 2H), 2.89 (dt, *J* = 13.8, 6.9 Hz, 1H), 2.65 (s, 2H), 2.13 (s, 3H), 1.36 (s, 6H), 1.23 (d, *J* = 6.9 Hz, 6H). ^13^C NMR (75 MHz, CDCl_3_) δ 206.81, 150.07, 137.69, 128.23, 126.87, 54.45, 46.93, 33.86, 32.26, 28.15, 23.90. HRMS (ESI-MS) [M + Na]^+^: found 273.1290; calculated for C_15_H_22_NaOS^+^: 273.1284.

4-((2,4-Dimethylphenyl)thio)-4-methylpentan-2-one (**4e**). Oil liquid, 89% yield. ^1^H NMR (300 MHz, CDCl_3_) δ 7.39 (d, *J* = 7.8 Hz, 1H), 7.11 (s, 1H), 6.97 (d, *J* = 7.7 Hz, 1H), 2.71 (s, 2H), 2.47 (s, 3H), 2.32 (s, 3H), 2.15 (s, 3H), 1.37 (s, 6H). ^13^C NMR (75 MHz, CDCl_3_) δ 206.94, 143.90, 139.50, 139.06, 131.58, 127.59, 126.92, 54.73, 48.46, 32.37, 28.21, 21.84, 21.25. HRMS (ESI-MS) [M + Na]^+^: found 259.1136; calculated for C_14_H_20_NaOS^+^: 259.1127.

4-((4-Methoxyphenyl)thio)-4-methylpentan-2-one (**4f**) [20]. Oil liquid, 87% yield. ^1^H NMR (300 MHz, CDCl_3_) δ 7.38 (d, *J* = 8.6 Hz, 2H), 6.82 (d, *J* = 8.6 Hz, 2H), 3.75 (s, 3H), 2.59 (s, 2H), 2.09 (s,3H), 1.31 (s, 6H). ^13^C NMR (75 MHz, CDCl_3_) δ 206.71, 160.47, 139.01, 122.19, 114.15, 55.24, 54.22, 46.74, 32.16, 27.94. HRMS (ESI-MS) [M + Na]^+^: found 261.0920; calculated for C_13_H_18_NaO_2_S^+^: 261.0920.

4-((4-Fluorophenyl)thio)-4-methylpentan-2-one (**4g**) [30]. Oil liquid, 76% yield. ^1^H NMR (300 MHz, CDCl_3_) δ 7.67–7.33 (m, 2H), 7.01 (t, *J* = 8.5 Hz, 2H), 2.62 (s, 2H), 2.12 (s, 3H), 1.34 (s, 6H). ^13^C NMR (75 MHz, CDCl_3_) δ 206.53 (s), 163.68 (d, *J* = 249.7 Hz), 139.63 (d, *J* = 8.4 Hz), 126.94 (d, *J* = 3.5 Hz), 115.93 (d, *J* = 21.6 Hz), 54.39, 47.20 (d, *J* = 1.3 Hz), 32.28, 28.18. ^19^F NMR (282 MHz, Chloroform-*d*) δ -111.91 (tt, *J* = 8.6, 5.5 Hz). HRMS (ESI-MS) [M + Na]^+^: found 249.0726;calculated for C_12_H_15_FNaOS^+^: 249.0720.

4-((4-Chlorophenyl)thio)-4-methylpentan-2-one (**4h**) [20]. Oil liquid, 85% yield. ^1^H NMR (300 MHz, CDCl_3_) δ 7.43 (d, *J* = 8.4 Hz, 2H), 7.30 (d, *J* = 8.4 Hz, 2H), 2.63 (s, 2H), 2.12 (s, 3H), 1.36 (s, 6H). ^13^C NMR (75 MHz, CDCl_3_) δ 206.35, 138.89, 135.74, 130.09, 128.99, 54.39, 47.45, 32.23, 28.24. HRMS (ESI-MS) [M + Na]^+^: found 265.0429; calculated for C_12_H_15_ClNaOS^+^: 265.0424.

4-((4-Bromophenyl)thio)-4-methylpentan-2-one (**4i**) [20]. Oil liquid, 79% yield. ^1^H NMR (300 MHz, CDCl_3_) δ 7.45 (d, *J* = 8.4 Hz, 2H), 7.35 (d, *J* = 8.4 Hz, 2H), 2.62 (s, 2H), 2.11 (s, 3H), 1.35 (s, 6H). ^13^C NMR (75 MHz, CDCl_3_) δ 206.32, 139.13, 131.93, 130.63, 124.02, 54.32, 47.40, 32.21, 28.21. HRMS (ESI-MS) [M + Na]^+^: found 308.9923; calculated for C_12_H_15_BrNaOS^+^: 308.9919.

4-((4-Iodophenyl)thio)-4-methylpentan-2-one (**4j**). Oil liquid, 76% yield. ^1^H NMR (300 MHz, CDCl_3_) δ 7.64 (d, *J* = 8.2 Hz, 2H), 7.21 (d, *J* = 8.2 Hz, 2H), 2.62 (s, 2H), 2.11 (s, 3H), 1.34 (s, 6H). ^13^C NMR (75 MHz, CDCl_3_) δ 206.26, 139.25, 137.85, 131.32, 95.94, 54.27, 47.38, 32.20, 28.18. HRMS (ESI-MS) [M + Na]^+^: found 356.9786; calculated for C_12_H_15_INaOS^+^: 356.9780.

4-Methyl-4-((4-(trifluoromethyl)phenyl)thio)pentan-2-one (**4k**). Oil liquid, 63% yield. ^1^H NMR (300 MHz, CDCl_3_) δ 7.62 (q, *J* = 8.4 Hz, 4H), 2.68 (s, 2H), 2.15 (s, 3H), 1.40 (s, 6H). ^13^C NMR (75 MHz, CDCl_3_) δ 206.28, 137.77, 136.40, 131.19 (q, *J* = 32.7 Hz), 125.59 (q, *J* = 3.6 Hz). 124.05 (q, J = 270.75 Hz), 54.46, 48.00, 32.25, 28.42. ^19^F NMR (282 MHz, CDCl_3_) δ = −62.74. HRMS (ESI-MS) [M + Na]^+^: found 299.0678; calculated for C_13_H_15_F_3_NaOS^+^: 299.0688.

4-Methyl-4-((4-nitrophenyl)thio)pentan-2-one (**4l**) [20]. Oil liquid, 68% yield. ^1^H NMR (300 MHz, CDCl_3_) δ 8.18 (d, *J* = 8.7 Hz, 2H), 7.68 (d, *J* = 8.7 Hz, 2H), 2.71 (s,2H), 2.15 (s, 3H), 1.44 (s, 6H). ^13^C NMR (75 MHz, CDCl_3_) δ 205.88, 148.19, 140.87, 137.59, 123.61, 54.47, 48.80, 32.21, 28.61. HRMS (ESI-MS) [M + Na]^+^: found 276.0668; calculated for C_12_H_15_NNaO_3_S^+^: 276.0665.

Methyl 4-((2-methyl-4-oxopentan-2-yl)thio)benzoate (**4m**). Oil liquid, 69% yield. ^1^H NMR (300 MHz, CDCl_3_) δ 7.92 (d, *J* = 8.1 Hz, 2H), 7.52 (d, *J* = 8.1 Hz, 2H), 3.84 (s, 3H), 2.61 (s, 2H), 2.06 (s, 3H), 1.33 (s, 6H). ^13^C NMR (75 MHz, CDCl_3_) δ 206.10, 166.44, 137.51, 137.08, 130.45, 129.56, 54.27, 52.22, 47.84, 32.06, 28.29. HRMS (ESI-MS) [M + Na]^+^: found 289.0872; calculated for C_14_H_18_NaO_3_S^+^: 289.0869.

4-(Benzylthio)-4-methylpentan-2-one (**4n**). Oil liquid, 85% yield. ^1^H NMR (300 MHz, CDCl_3_) δ 7.46–7.27 (m, 5H), 3.84 (s, 2H), 2.74 (s, 2H), 2.19 (s, 3H), 1.52 (s, 6H). ^13^C NMR (75 MHz, CDCl_3_) δ 206.76, 137.99, 129.03, 128.57, 127.00, 54.56, 44.32, 33.32, 32.28, 28.49. HRMS (ESI-MS) [M + Na]^+^: found 245.0978; calculated for C_13_H_18_NaOS^+^: 245.0971.

4-Methyl-4-((4-methylbenzyl)thio)pentan-2-one (**4o**). Oil liquid, 79% yield. ^1^H NMR (300 MHz, CDCl_3_) δ 7.22 (d, *J* = 7.9 Hz, 2H), 7.10 (d, *J* = 7.8 Hz, 2H), 3.75 (s, 2H), 2.69 (s, 2H), 2.31 (s, 3H), 2.14 (s, 3H), 1.46 (s, 6H). ^13^C NMR (75 MHz, CDCl_3_) δ 206.67, 136.47, 134.72, 129.17, 128.83, 54.47, 44.16, 32.89, 32.20, 28.42, 21.04. HRMS (ESI-MS) [M + Na]^+^: found 259.1130; calculated for C_14_H_20_NaOS^+^: 259.1127.

4-((4-Methoxybenzyl)thio)-4-methylpentan-2-one (**4p**) [31]. Oil liquid, 52% yield. ^1^H NMR (300 MHz, CDCl_3_) δ 7.33 (d, *J* = 8.6 Hz, 2H), 6.91 (d, *J* = 8.6 Hz, 2H), 3.86 (s, 3H), 3.82 (s, 2H), 2.77 (s, 2H), 2.24 (s, 3H), 1.53 (s, 6H). ^13^C NMR (75 MHz, CDCl_3_) δ 206.95, 158.66, 130.14, 129.79, 114.04, 55.35, 54.68, 44.30, 32.70, 32.40, 28.56. HRMS (ESI-MS) [M + Na]^+^: found 275.0924; calculated for C_14_H_20_NaO_2_S^+^: 275.1076.

4-((4-Chlorobenzyl)thio)-4-methylpentan-2-one (**4q**) [31]. Oil liquid, 85% yield. ^1^H NMR (300 MHz, CDCl_3_) δ 7.29 (s,4H), 3.77 (s, 2H), 2.72 (s, 2H), 2.18 (s, 3H), 1.47 (s, 6H). ^13^C NMR (75 MHz, CDCl_3_) δ 206.52, 136.56, 132.65, 130.35, 128.63, 54.54, 44.43, 32.58, 32.24, 28.48. HRMS (ESI-MS) [M + Na]^+^: found 279.0586; calculated for C_13_H_17_ClNaOS^+^: 279.0581.

4-(Butylthio)-4-methylpentan-2-one (**4r**). Oil liquid, 36% yield. ^1^H NMR (300 MHz, CDCl_3_) δ 2.66 (s, 2H), 2.51 (t, *J* = 7.3 Hz, 2H), 2.16 (s, 3H), 1.59–1.45 (m, 2H), 1.45–1.29 (m, 8H), 0.89 (t, *J* = 7.2 Hz, 3H). ^13^C NMR (75 MHz, CDCl_3_) δ 207.10, 54.73, 43.35, 32.46, 31.64, 28.57, 27.86, 22.43, 13.81. HRMS (ESI-MS) [M + Na]^+^: found 211.1126; calculated for C_10_H_20_NaOS^+^: 211.1127.

4-(Cyclohexylthio)-4-methylpentan-2-one (**4s**). Oil liquid, 41% yield. ^1^H NMR (300 MHz, CDCl_3_) δ 2.67 (s, 2H), 2.66–2.57 (m, 1H), 2.15 (s, 3H), 1.91 (d, *J* = 9.0 Hz, 2H), 1.73–1.64 (m, 2H), 1.56–1.49 (m, 1H), 1.39 (s, 6H), 1.35–1.09 (m, 5H).^13^C NMR (75 MHz, CDCl_3_) δ 207.05, 55.50, 44.70, 41.22, 36.24, 32.54, 29.07, 26.44, 25.52. HRMS (ESI-MS) [M + Na]^+^: found 237.1288; calculated for C_12_H_22_NaOS^+^: 237.1284.

4-Methyl-4-(naphthalen-2-ylthio)pentan-2-one (**4t**) [29]. Oil liquid, 85% yield. ^1^H NMR (300 MHz, CDCl_3_) δ 8.08 (s, 1H), 7.89–7.73 (m, 3H), 7.59 (dd, *J* = 8.4, 1.4 Hz, 1H), 7.51 (dd, *J* = 6.2, 3.3 Hz, 2H), 2.72 (s, 2H), 2.14 (s, 3H), 1.46 (s, 6H). ^13^C NMR (75 MHz, CDCl_3_) δ 206.52, 137.45, 134.17, 133.32, 133.16, 128.85, 128.05, 127.91, 127.61, 126.94, 126.43, 54.38, 47.51, 32.16, 28.30. HRMS (ESI-MS) [M + Na]^+^: found 281.0980; calculated for C_16_H_18_NaOS^+^: 281.0971.

4-Methyl-4-(thiophen-2-ylthio)pentan-2-one (**4u**). Oil liquid, 21% yield. ^1^H NMR (300 MHz, CDCl_3_) δ 7.45 (dd, *J* = 5.4, 0.9 Hz, 1H), 7.17 (dd, *J* = 3.4, 0.9 Hz, 1H), 7.06 (dd, *J* = 5.3, 3.6 Hz, 1H), 2.70 (s, 2H), 2.16 (s, 3H), 1.41 (s, 6H). ^13^C NMR (75 MHz, CDCl_3_) δ 206.68, 137.63, 131.52, 130.63, 127.87, 54.04, 48.05, 32.30, 27.8 HRMS (ESI-MS) [M + Na]^+^: found 237.0383; calculated for C_10_H_14_NaOS_2_^+^: 237.0378.

## 4. Conclusions

In summary, the synthesis methods of thioacetal, thioacetone, and 4-methyl-4-(arylsulfide)pentane-2-ketone promoted by methanesulfonic anhydride/sulfuric acid were discussed under mild conditions. In this paper, the mechanism of reaction is explored, and it is suggested that two reaction processes may have occurred simultaneously. Notably, using this synthetic approach eliminates the need for the prior preparation of α, β-unsaturated ketones. This strategy is characterized as simple and efficient while demonstrating good substrate compatibility. It is an effective method to prepare thioacetals/thioketals and thio-Michael addition products.

## Figures and Tables

**Table 1:**
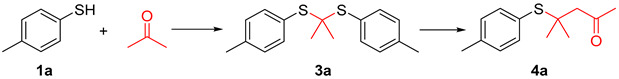
Optimization of reaction conditions *^a^*.

Entry	Acid (eq.)	T (°C)	Solvent	Yield of 3a (%) *^b^*	Yield of 4a (%) *^c^*
1	Ts_2_O (1)	60	Acetone	7	72
2	Ms_2_O (1)	60	Acetone	62	3
3	TsOH (1)	60	Acetone	-	78
4	Hydrochloric acid (1)	60	Acetone	35	4
5	H_2_SO_4_ (1)	60	Acetone	-	88
6	H_2_SO_4_ (1)	60	Acetone + DCE	-	95 (86) *^d^*
7	H_2_SO_4_ (0.1)	60	Acetone + DCE	--	21
8	H_2_SO_4_ (0.5)	60	Acetone + DCE	-	74
9	H_2_SO_4_ (1)	60	Acetone + DCM	-	91
10	H_2_SO_4_ (1)	60	Acetone + Toluene	-	92
11	H_2_SO_4_ (1)	60	Acetone + H_2_O	-	1
12	H_2_SO_4_ (1)	60	Acetone + CH_3_CN	-	26
13	H_2_SO_4_ (1)	60	Acetone + EtOAc	-	49
14	H_2_SO_4_ (1)	60	Acetone + THF	-	0
15	H_2_SO_4_ (1)	rt	Acetone + DCE	-	15
16	H_2_SO_4_ (1)	40	Acetone + DCE	-	42
17	H_2_SO_4_ (1)	50	Acetone + DCE	-	78
18	H_2_SO_4_ (1)	70	Acetone + DCE	-	92

*^a^* Reaction conditions. Entries 1–5: **1a** (0.2 mmol), acid (0.2 mmol), acetone (2 mL), 60 °C, 24 h; entries 6–14: **1a** (0.2 mmol), H_2_SO_4_, acetone:solvent = 1:5 (2 mL), 60 °C, 24 h; *^b^* GC-MS; *^c^* LC-MS; *^d^* isolated yield.

**Table 2:**
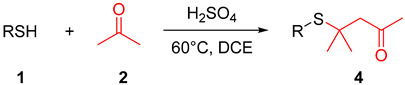
H_2_SO_4_ facilitated thia-Michael addition between thiols and ketones *^a^*^,^*^d^*.

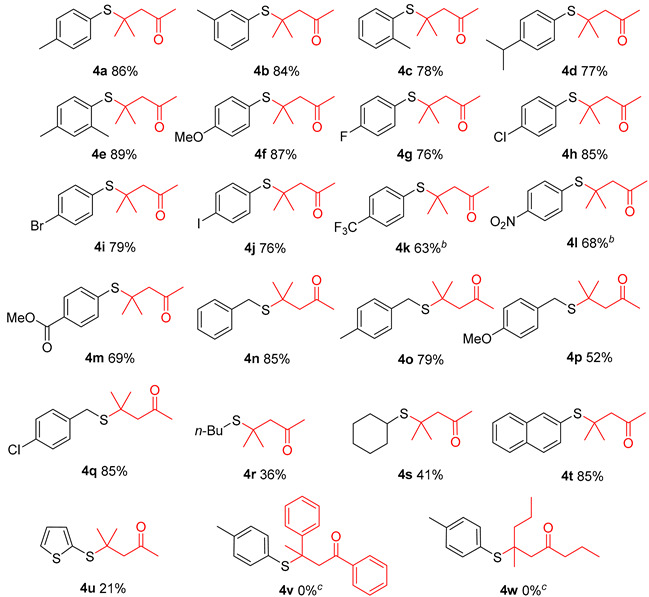

*^a^* Reaction conditions: **1** (0.6 mmol), H_2_SO_4_ (0.6 mmol), acetone:DCE = 1:5 (6 mL), 60 °C, 24 h, *^b^* 28 h; *^c^* reaction conditions: **1** (0.6 mmol), acetophenone/2-pentanone (0.9 mmol), H_2_SO_4_ (0.6 mmol), DCE (6 mL), 60 °C, 24 h. *^d^* Isolated yield.

**Table 3:**
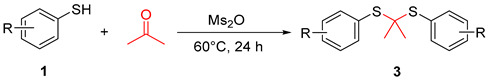
Ms_2_O facilitated thioacetalization of acetone to thioacetals *^a^*^,*b*^.

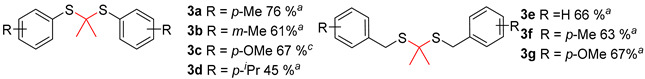

*^a^* Reaction conditions: **1** (0.6 mmol), Ms_2_O (0.6 mmol), acetone: DCE = 1:5 (6 mL), 60 °C, 20 h; *^b^* isolated yield; *^c^* 8 h.

**Table 4:**
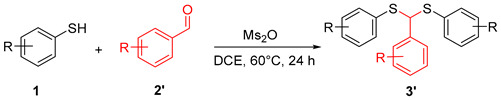
Ms_2_O facilitated thioacetalization of aldehyde to thioacetals ^*a*,*b*^.

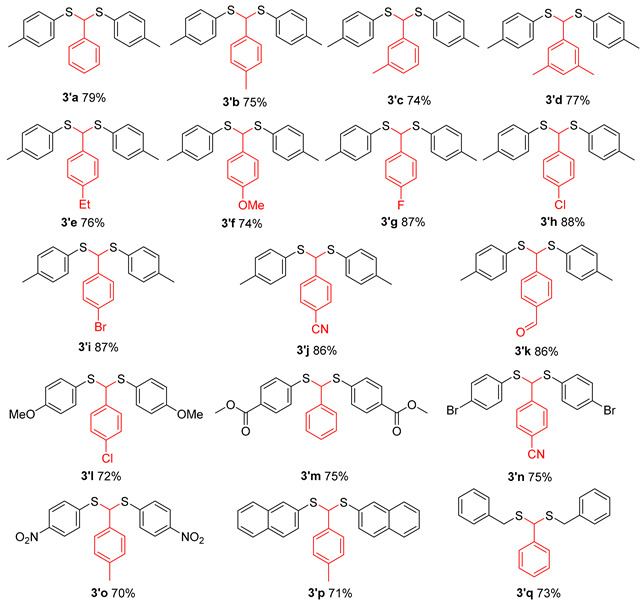

*^a^* Reaction conditions: **1** (0.6 mmol), **2′** (0.9 mmol), Ms_2_O (0.6 mmol), DCE (5 mL), 60 °C, 20 h; *^b^* isolated yield.

## Data Availability

The original contributions presented in this study are included in the article; further inquiries can be directed to the corresponding author/s.

## Supplementary Materials

The following supporting information can be downloaded at https://www.mdpi.com/article/10.3390/molecules29204785/s1: NMR spectra of products.
