# Supplementary material for: Controllable Synthesis of Thioacetals/Thioketals and β-Sulfanyl Ketones Mediated by Methanesulfonic Anhydride and Sulfuric Acid Sulfuric Acid from Aldehyde/Acetone and Thiols
Source: Molecules. 2024 Oct 10;29(20):4785. doi: 10.3390/molecules29204785 (PMC11510270; doi:10.3390/molecules29204785)

Supplementary Information

Controllable Synthesis of Thioacetals/Thioketals and β-Sulfanyl Ketones Mediated by Methanesulfonic Anhydride and H_2SO_4 from Aldehyde/Acetone and Thiols

Hexia Ye [1], Xinyao Zhao [1], Yajie Fu [1, 2], Haibo Liu [1], Junchen Li [1,*] and Xiaojing Bi [1,*]

[1] AffiliationState Key Laboratory of NBC Protection for Civilian, Beijing 102205, China
[2] School of Chemistry and Environmental Engineering, Sichuan University of Science &Engineering, 180 Xueyuan Street, Huixing Lu, Zigong, Sichuan 643000, China.
* Correspondence: xiaojingbimail@yeah.net (X.B.); lijch07@163.com (J.L.)

Copies of ^{1}H NMR, ^{13}C NMR and ^{19}F NMR spectra

^{1}H NMR of propane-2,2-diylbis(*p*-tolylsulfane) (**3a**).

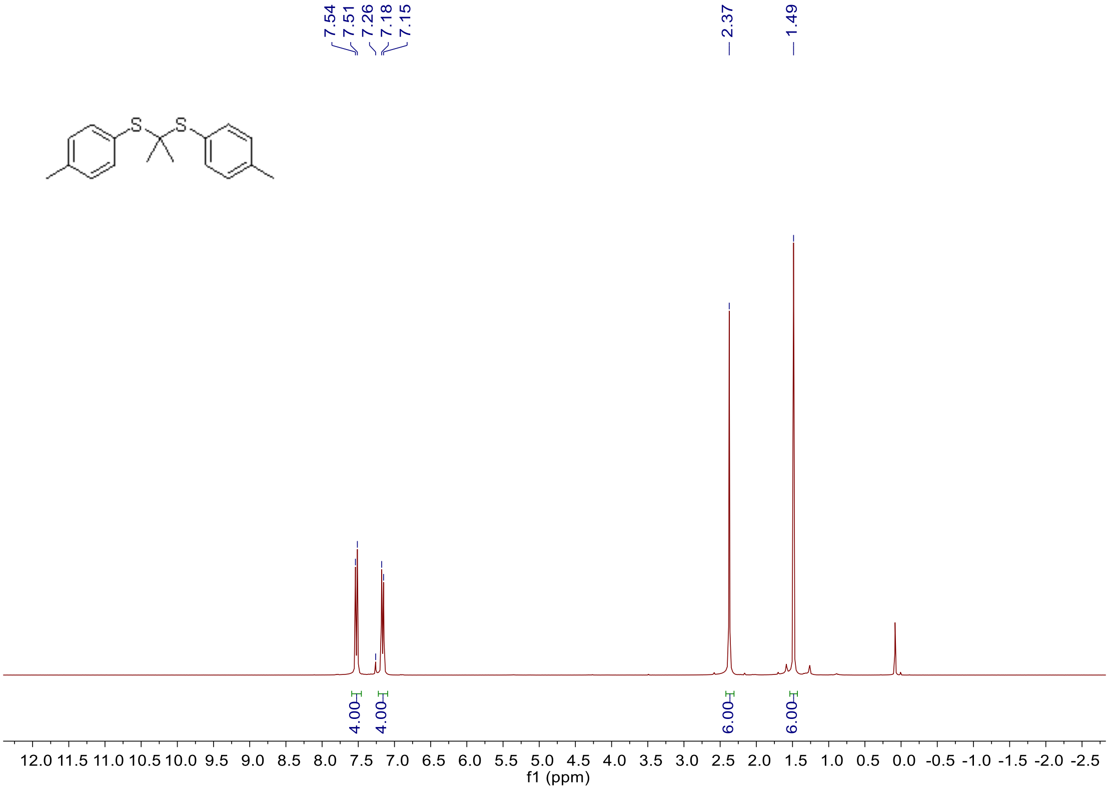

^{13}C NMR of propane-2,2-diylbis(*p*-tolylsulfane) (**3a**).

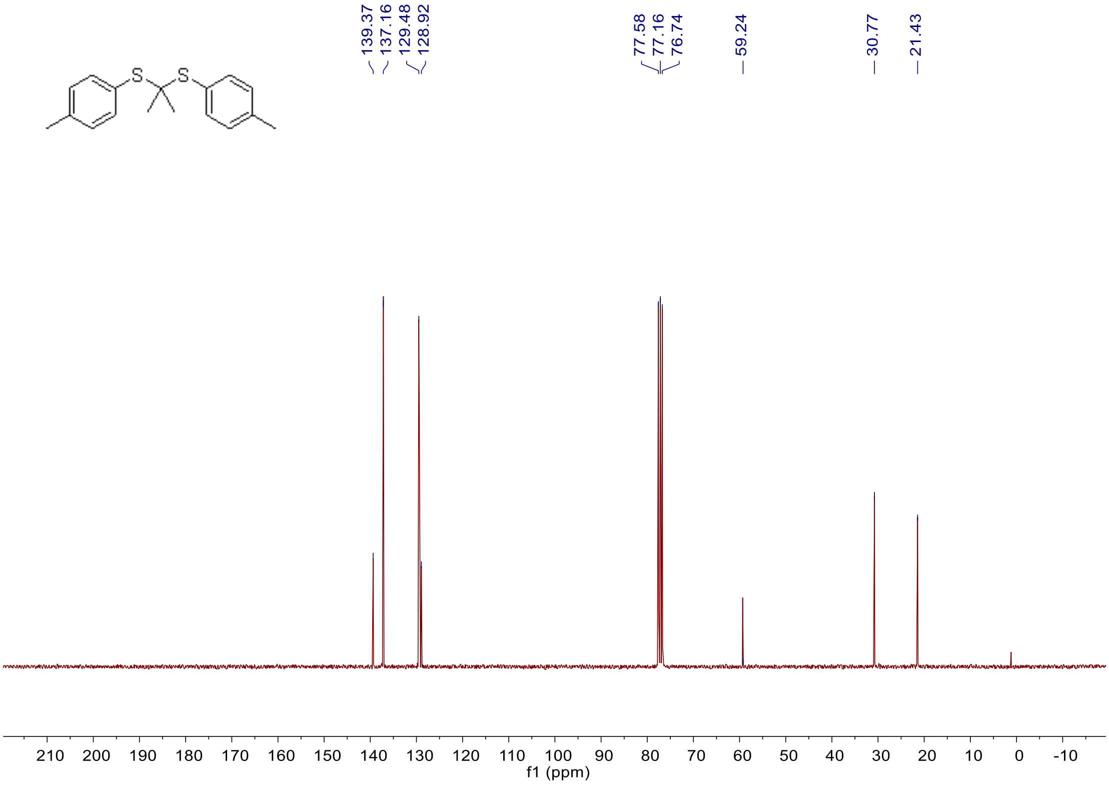

^{1}H NMR of propane-2,2-diylbis(*m*-tolylsulfane) (**3b**)

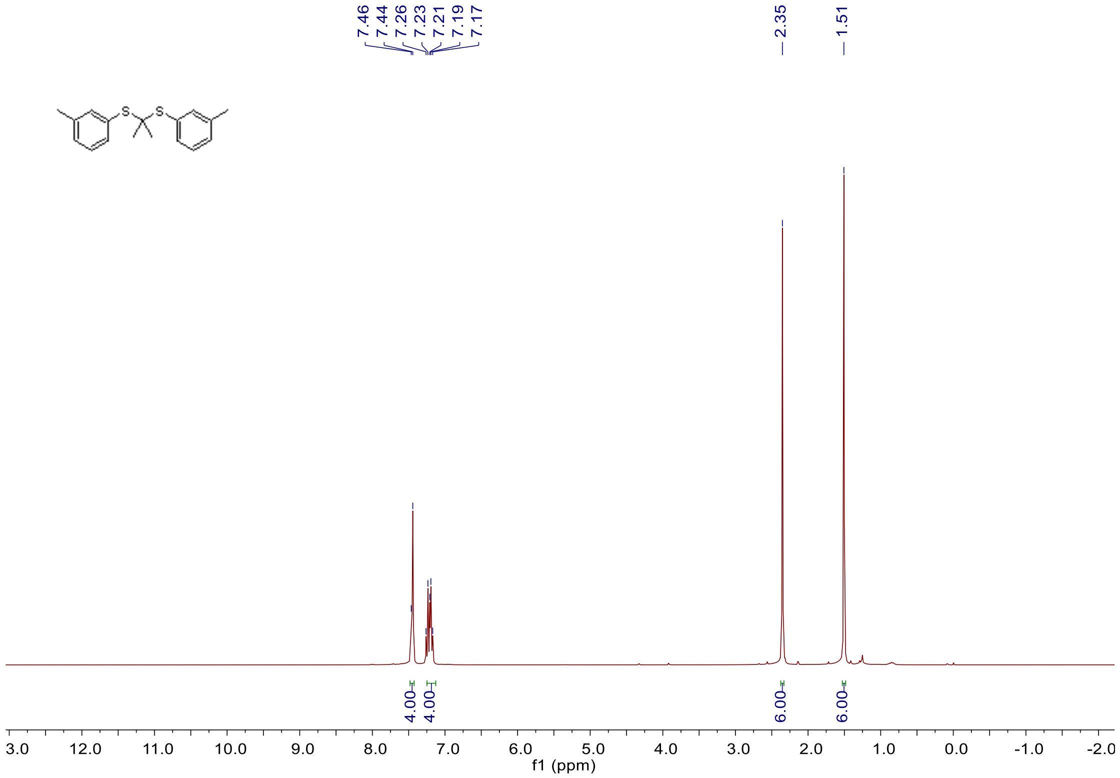

^{13}C NMR of propane-2,2-diylbis(*m*-tolylsulfane) (**3b**)

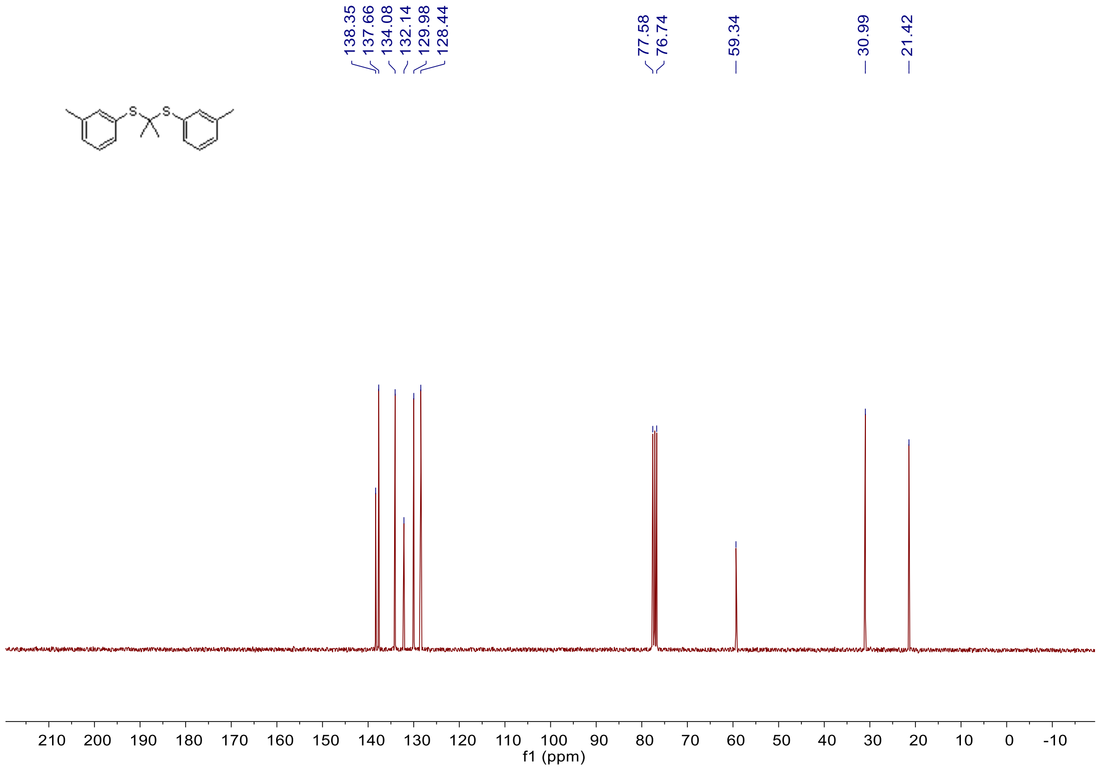

^{1}H NMR of propane-2,2-diylbis((4-methoxyphenyl)sulfane) (**3c**).

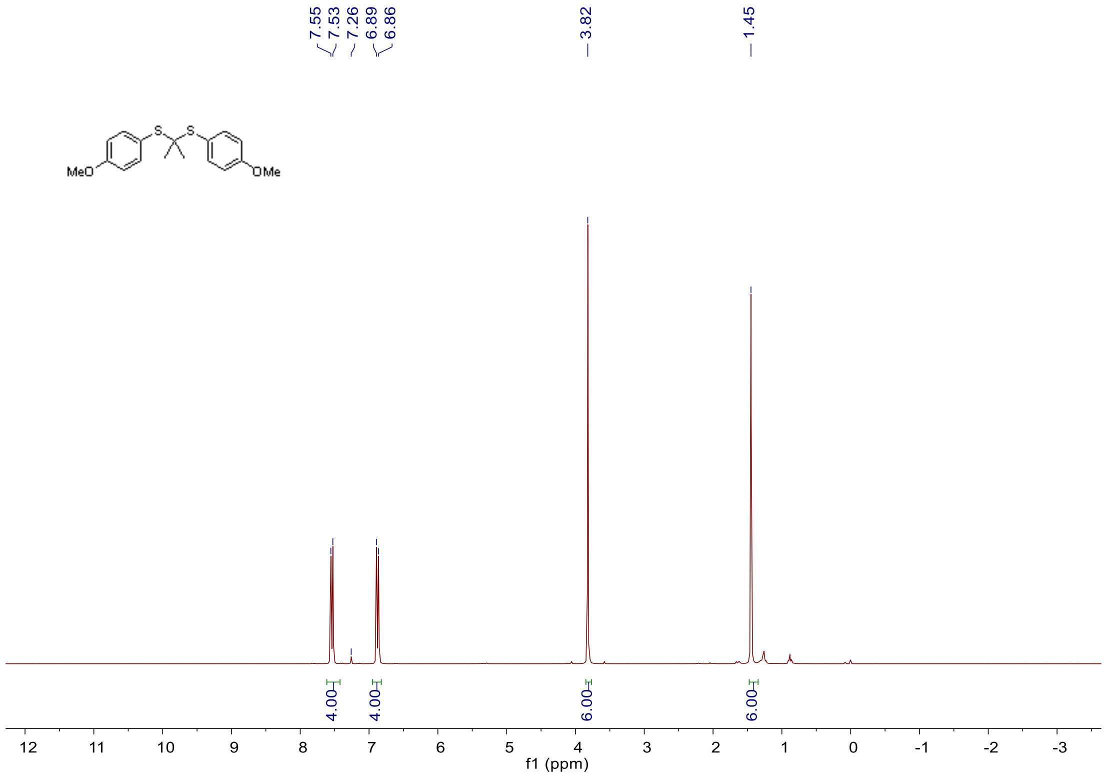

^{13}C NMR of propane-2,2-diylbis((4-methoxyphenyl)sulfane) (**3c**).

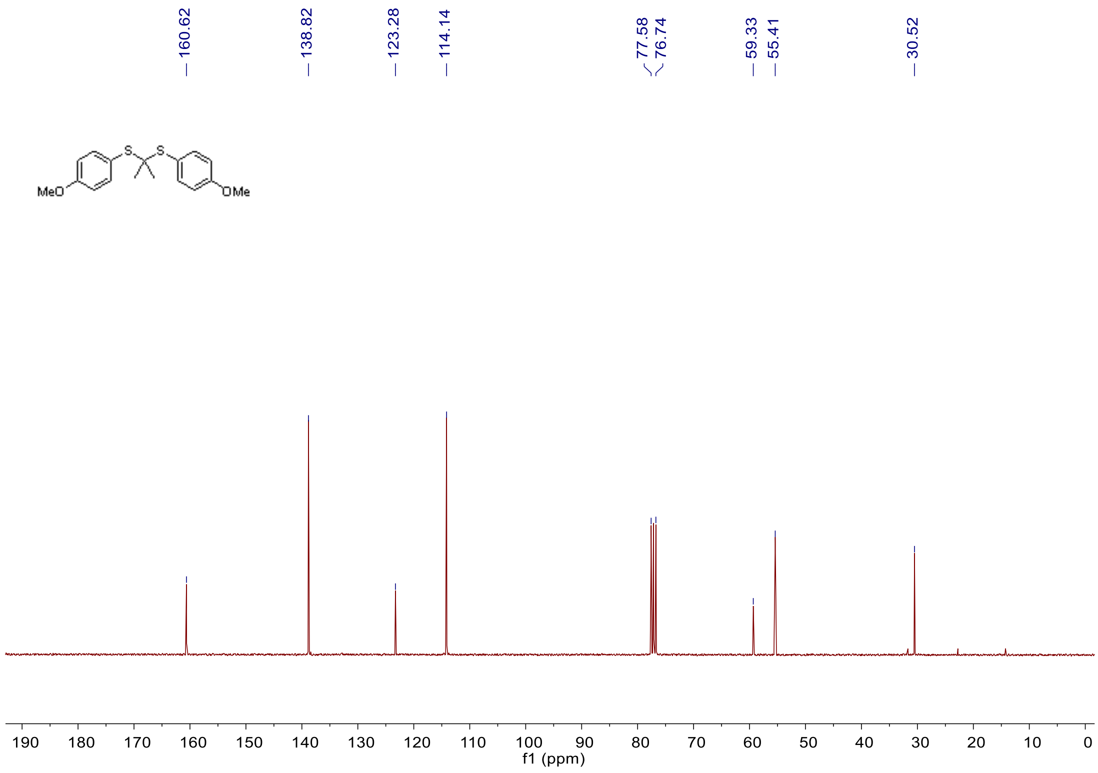

^{1}H NMR of propane-2,2-diylbis((4-isopropylphenyl)sulfane) (**3d**).

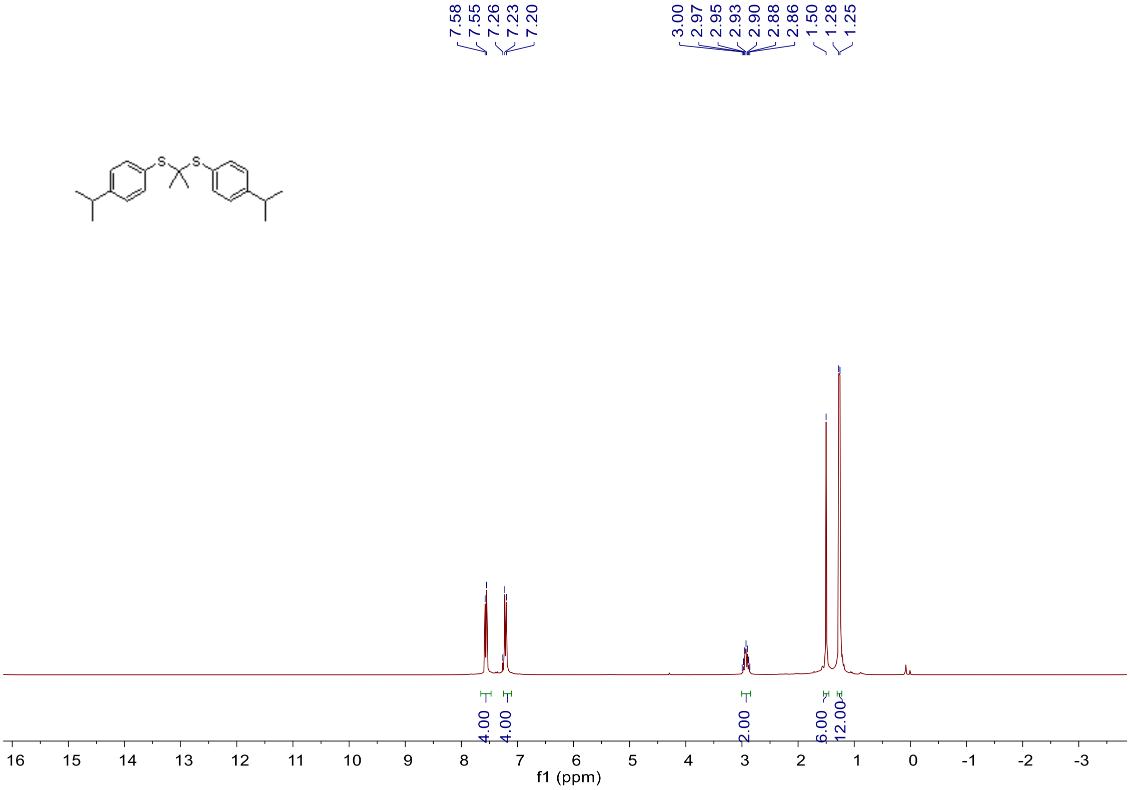

^{13}C NMR of propane-2,2-diylbis((4-isopropylphenyl)sulfane) (**3d**).

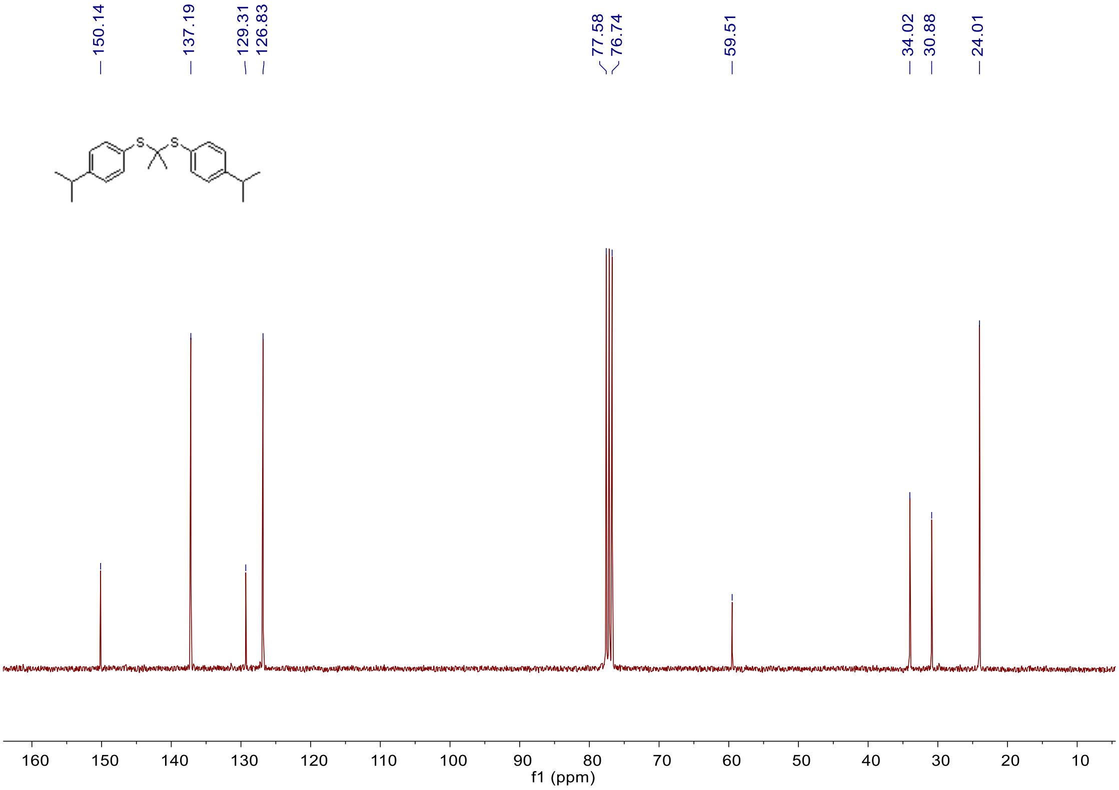

^{1}H NMR of propane-2,2-diylbis(benzylsulfane) (**3e**).

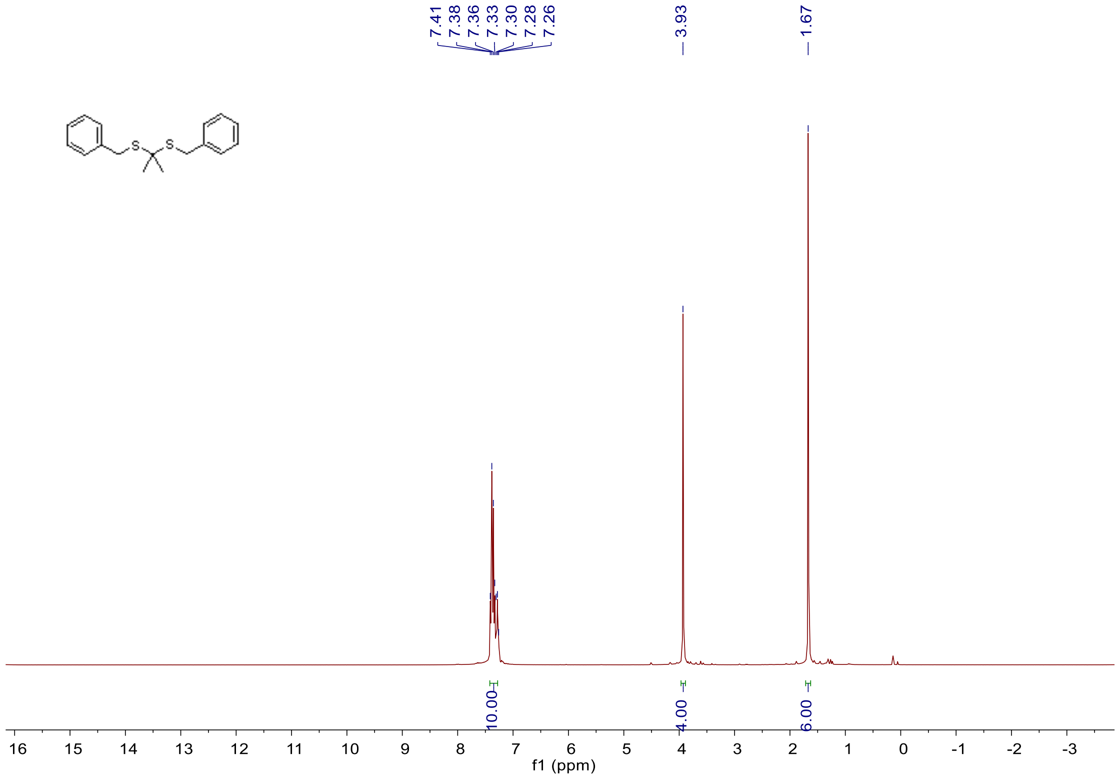

^{13}C NMR of propane-2,2-diylbis(benzylsulfane) (**3e**).

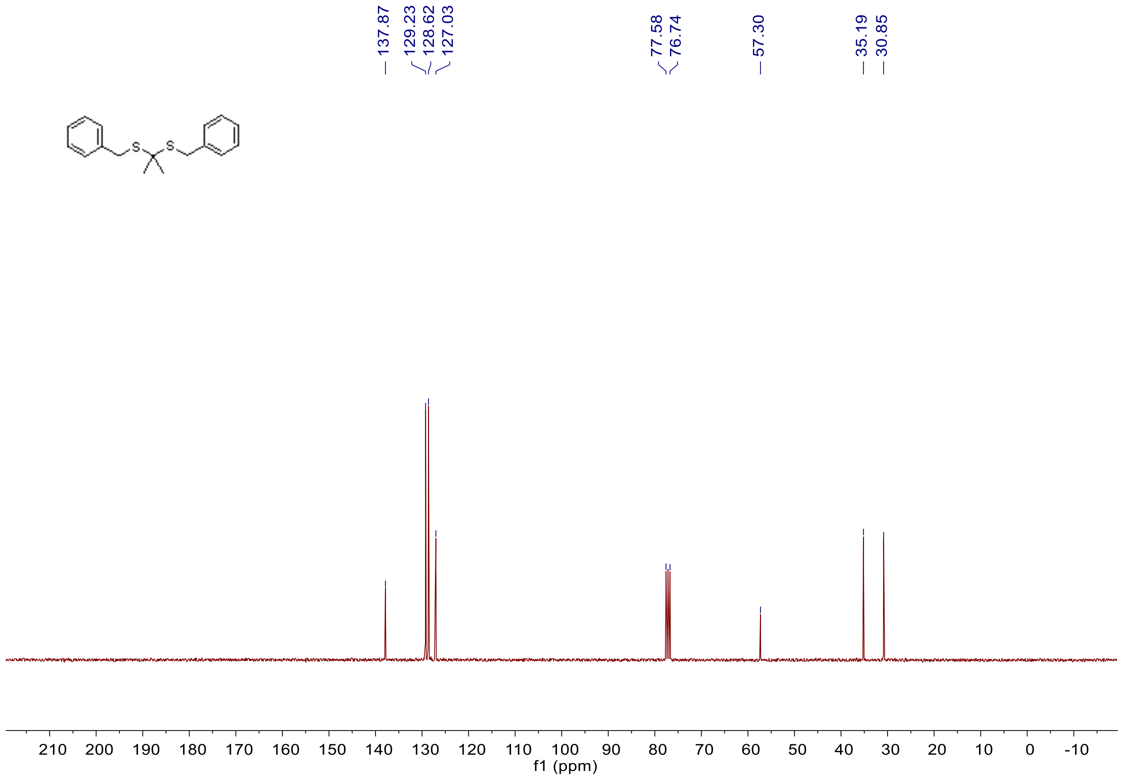

^{1}H NMR of propane-2,2-diylbis((4-methylbenzyl)sulfane) (**3f**).

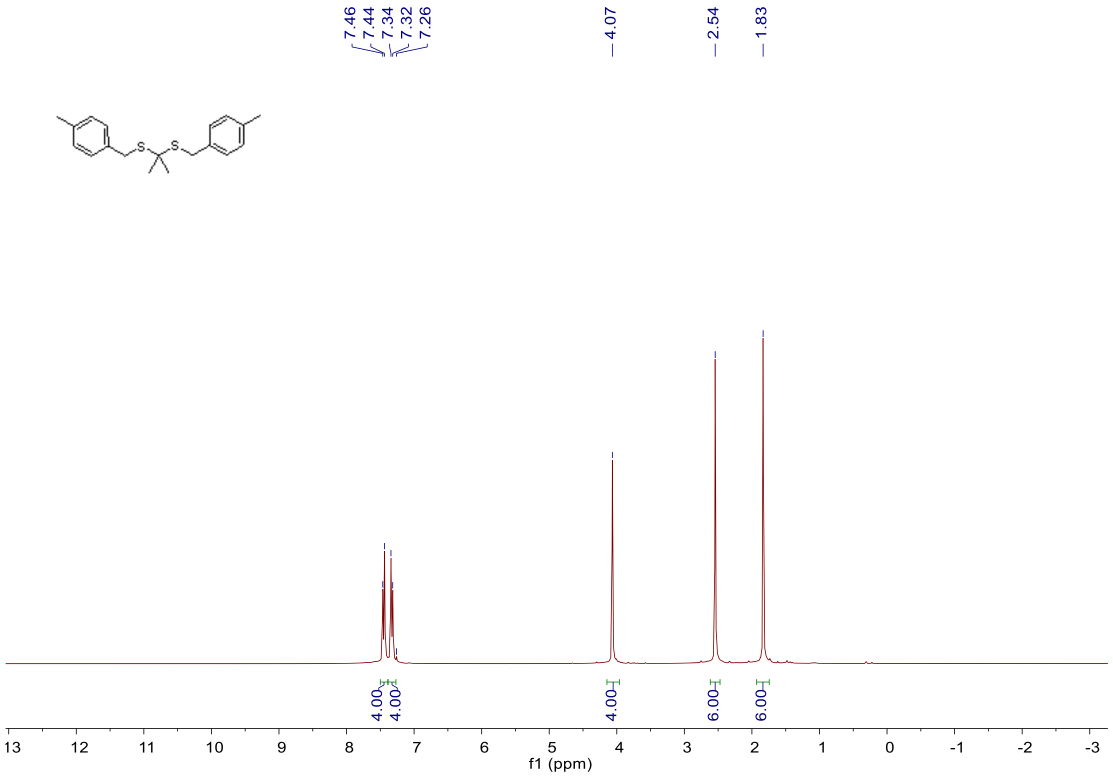

^{13}C NMR of propane-2,2-diylbis((4-methylbenzyl)sulfane) (**3f**).

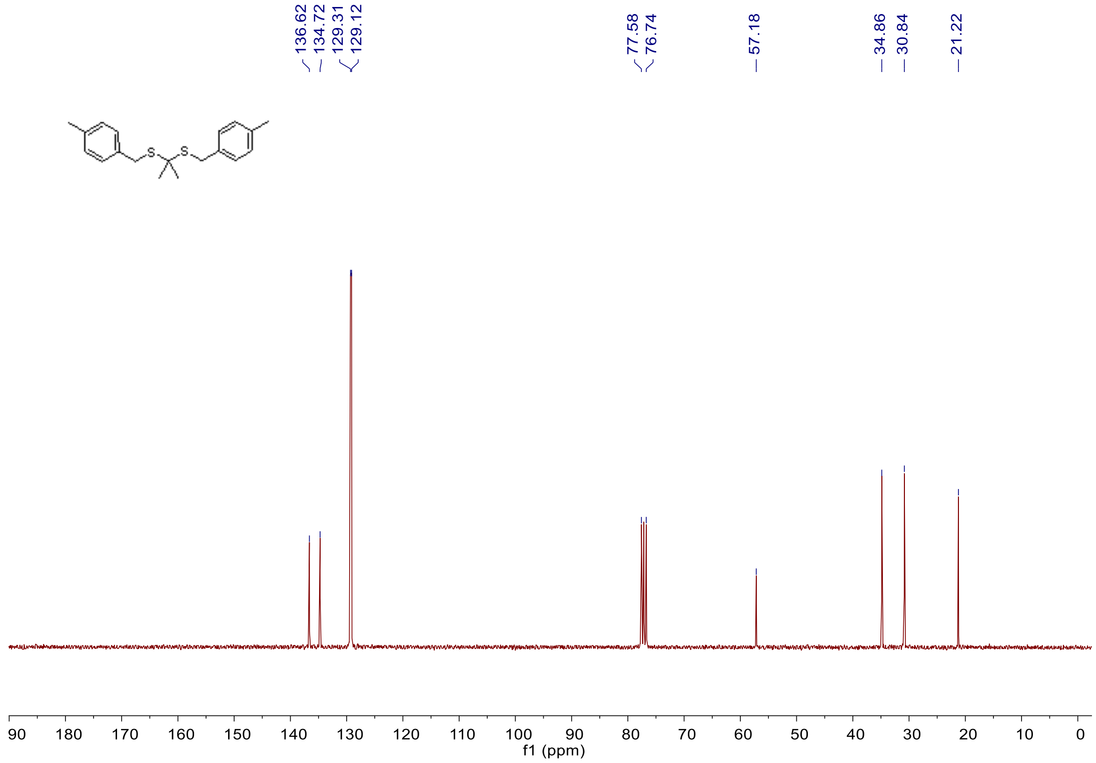

^{1}H NMR of propane-2,2-diylbis((4-methoxybenzyl)sulfane) (**3g**).

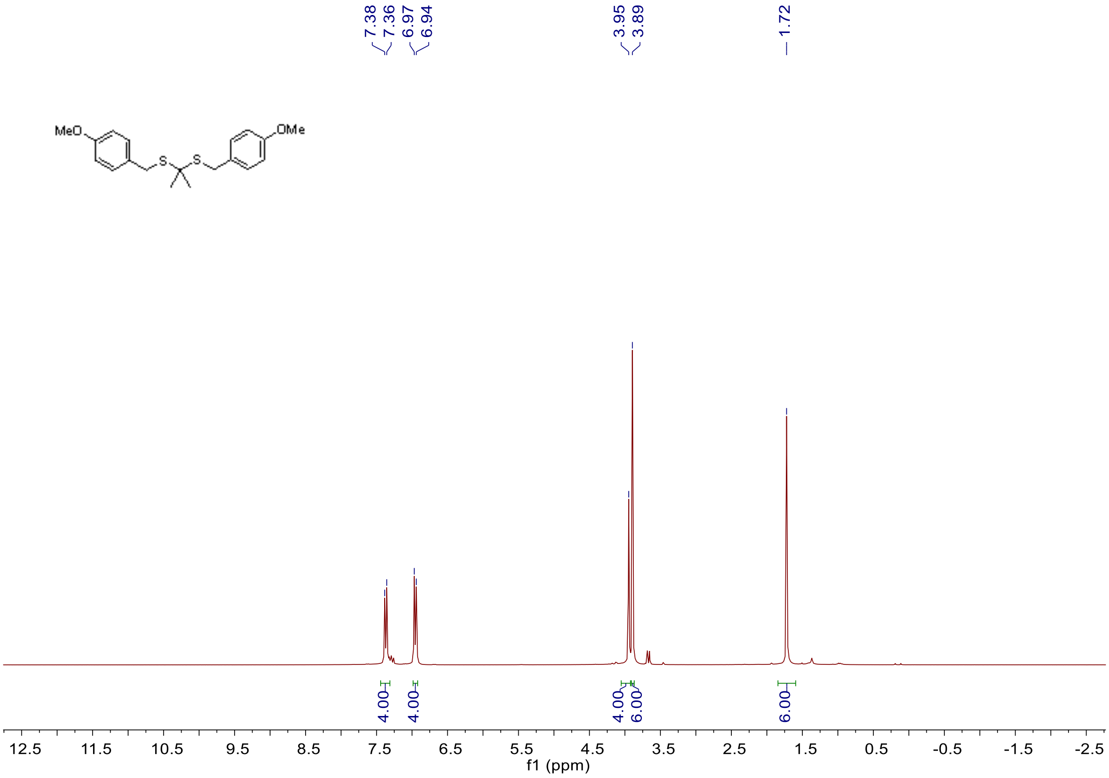

^{13}C NMR of propane-2,2-diylbis((4-methoxybenzyl)sulfane) (**3g**).

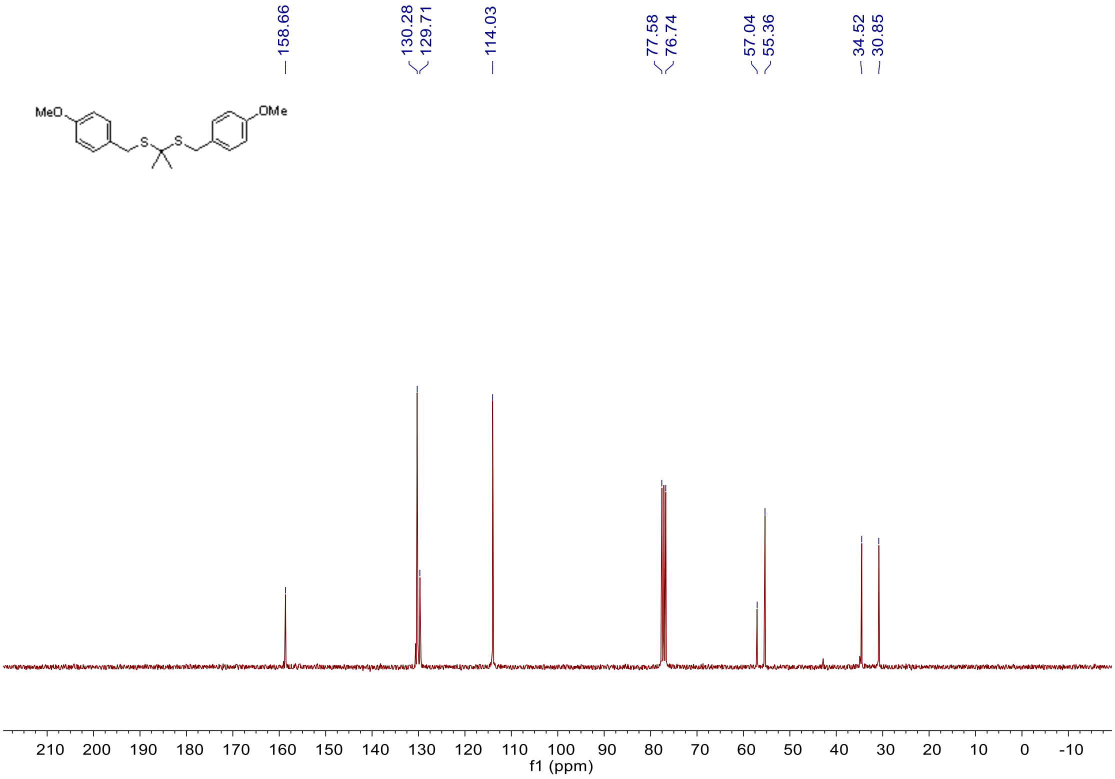

^{1}H NMR of (phenylmethylene)bis(p-tolylsulfane) (**3′a**)

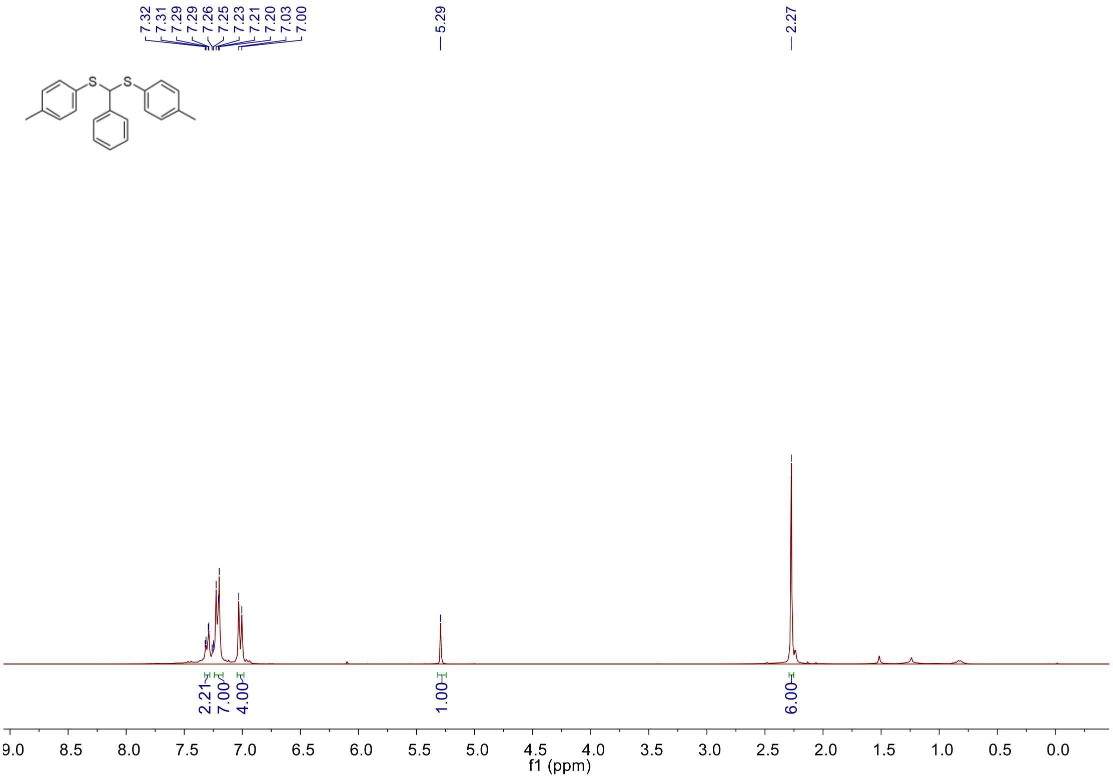

^{13}C NMR of (phenylmethylene)bis(p-tolylsulfane) (**3′a**)

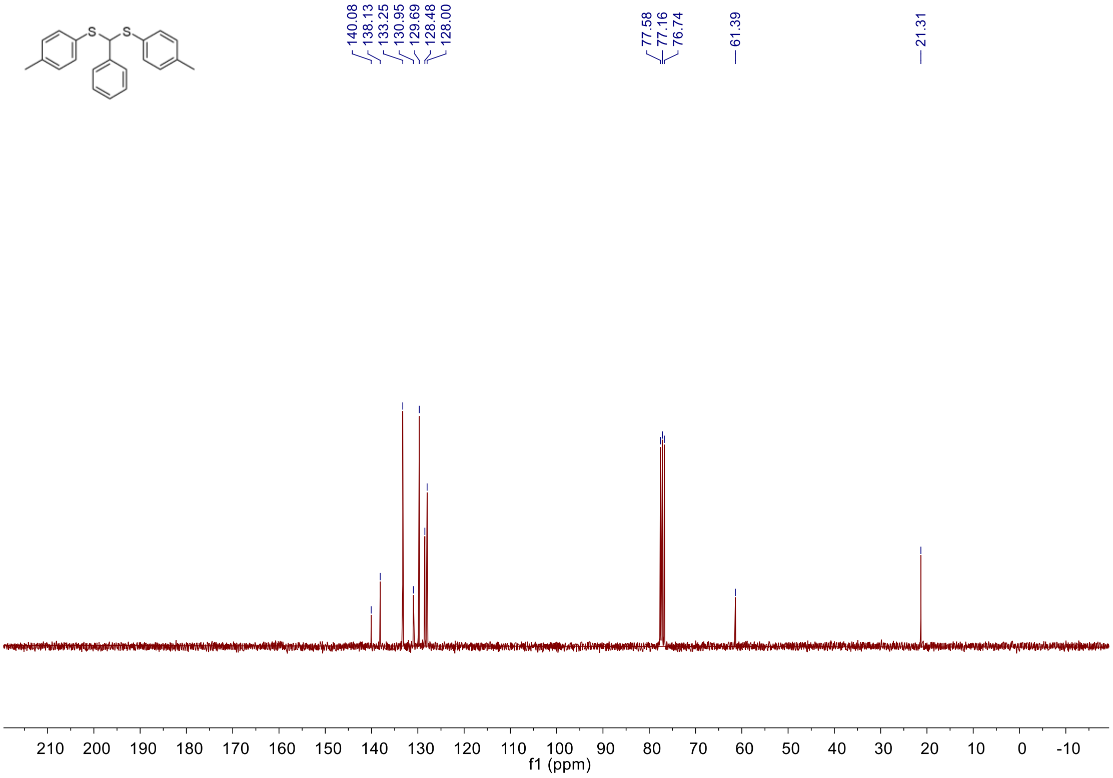

^{1}H NMR of (p-tolylmethylene)bis(p-tolylsulfane) (**3′b**)

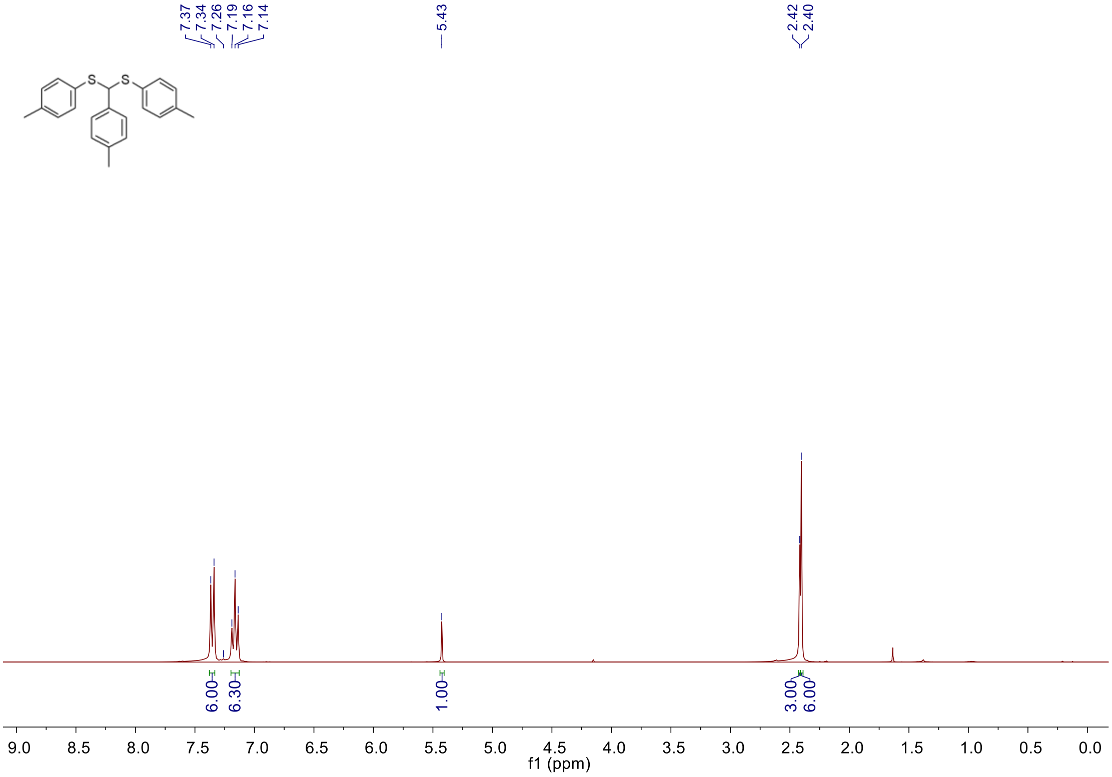

^{13}C NMR of (p-tolylmethylene)bis(p-tolylsulfane) (**3′b**)

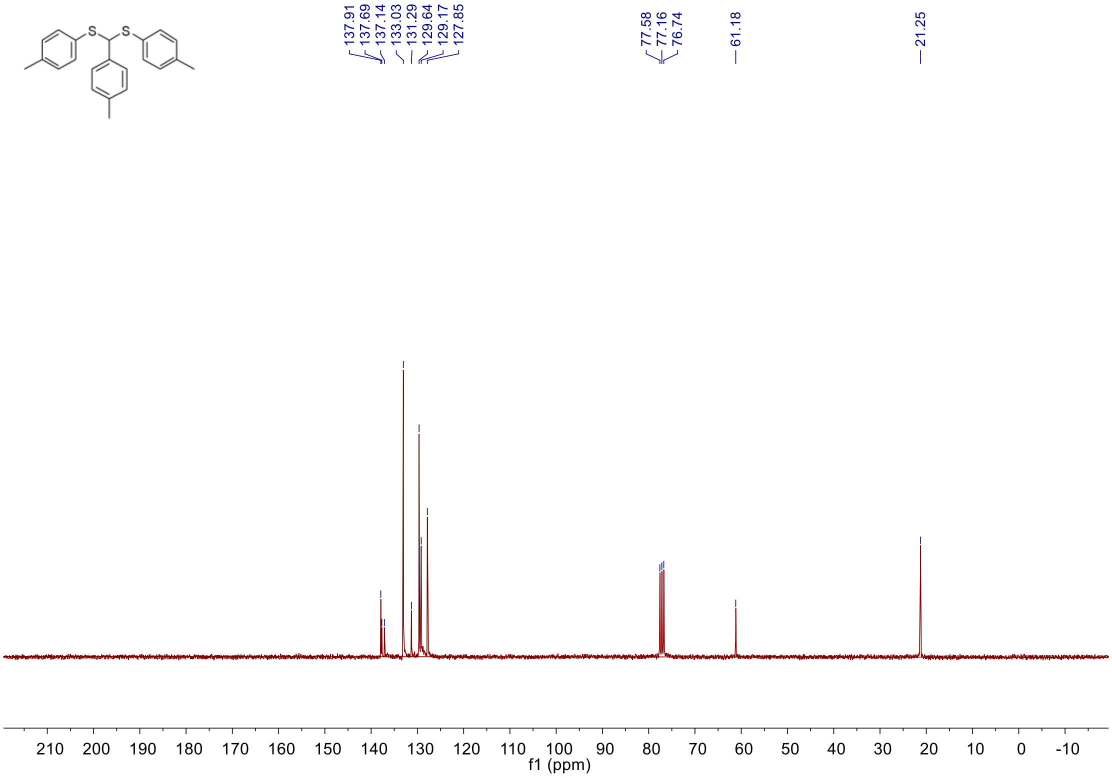

^{1}H NMR of (m-tolylmethylene)bis(p-tolylsulfane) (**3′c**)

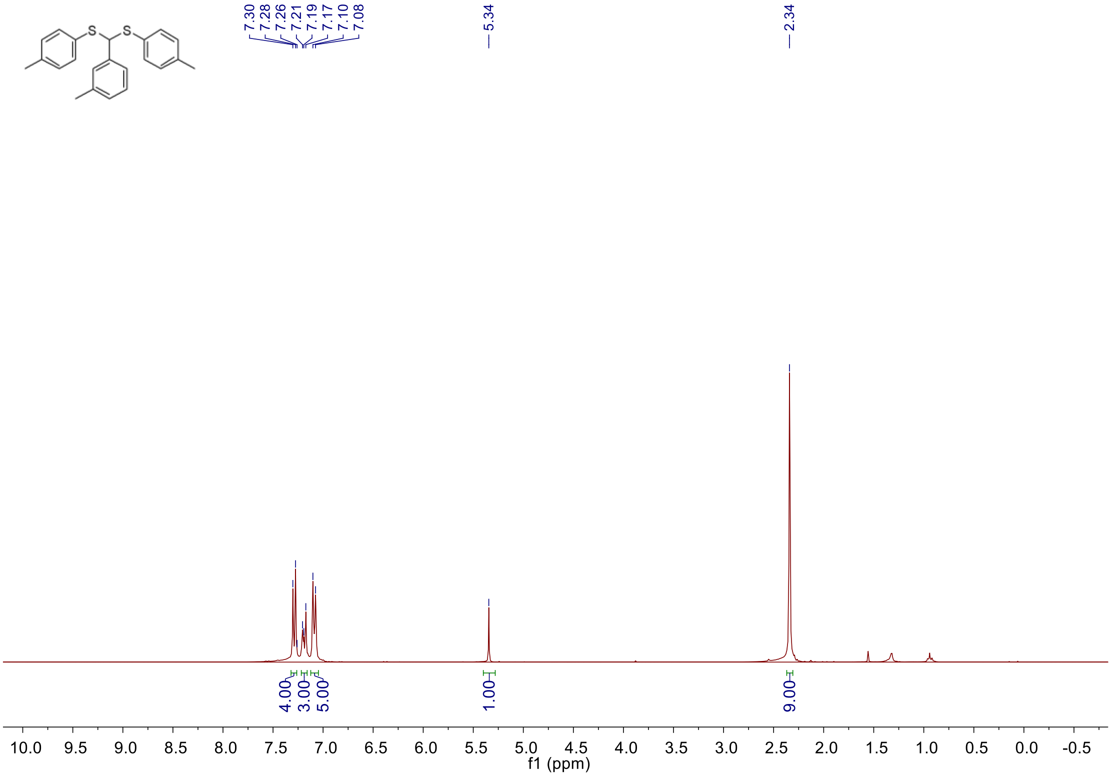

^{13}C NMR of (m-tolylmethylene)bis(p-tolylsulfane) (**3′c**)

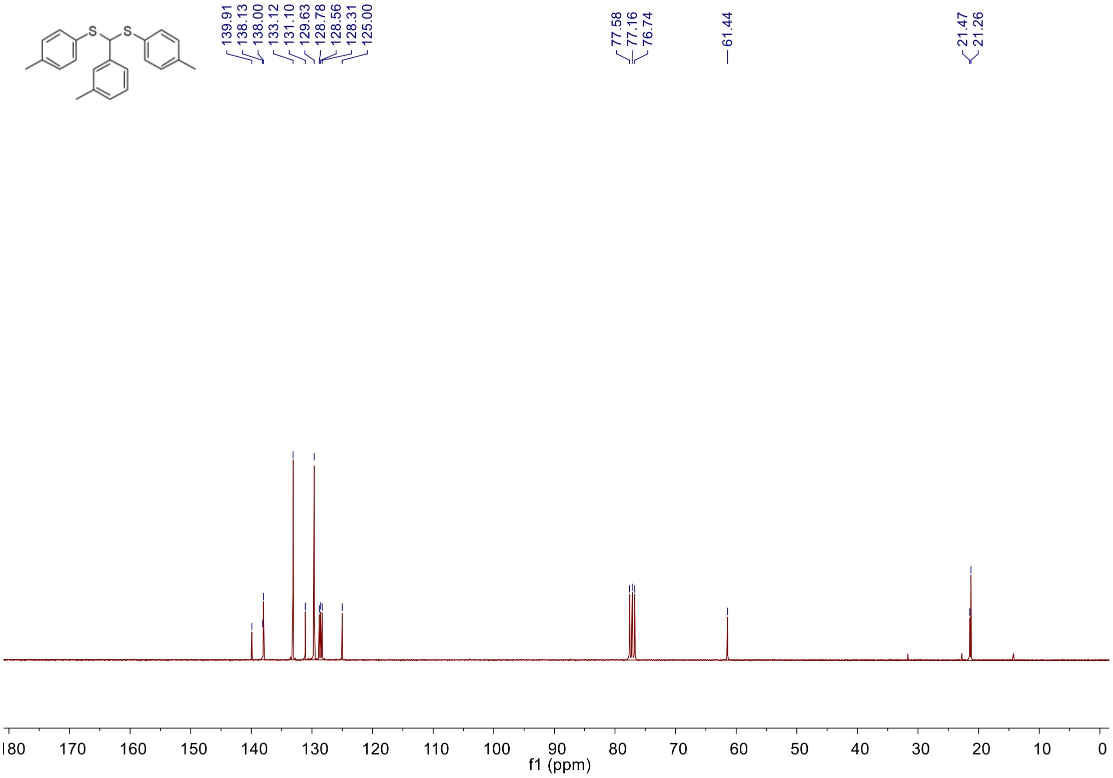

^{1}H NMR of ((3,5-dimethylphenyl)methylene)bis(p-tolylsulfane) (**3′d**)

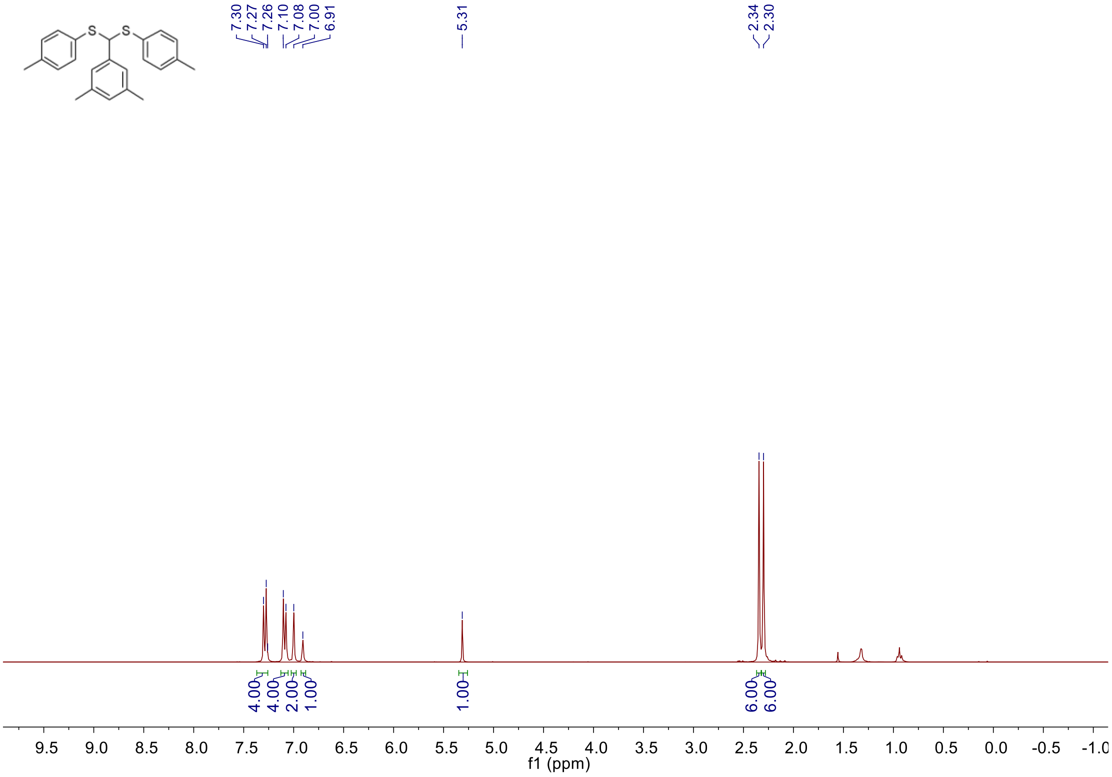

^{13}C NMR of ((3,5-dimethylphenyl)methylene)bis(p-tolylsulfane) (**3′d**)

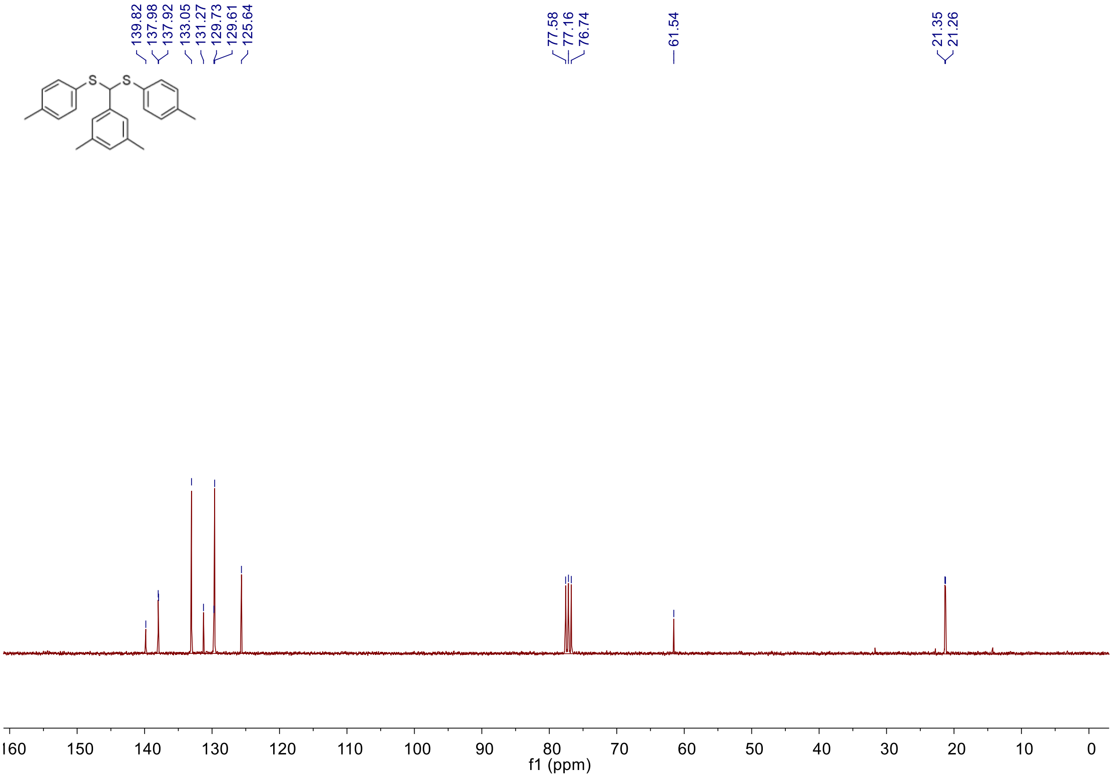

^{1}H NMR of ((4-ethylphenyl)methylene)bis(p-tolylsulfane) (**3′e**)

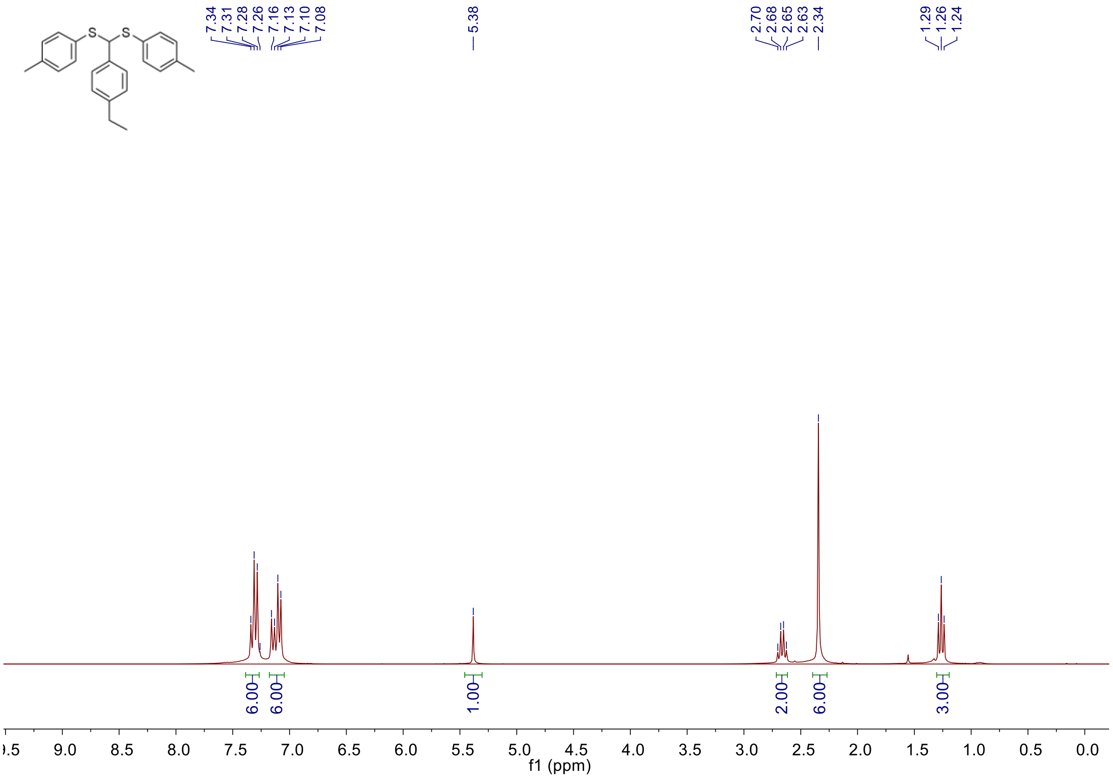

^{13}C NMR of ((4-ethylphenyl)methylene)bis(p-tolylsulfane) (**3′e**)

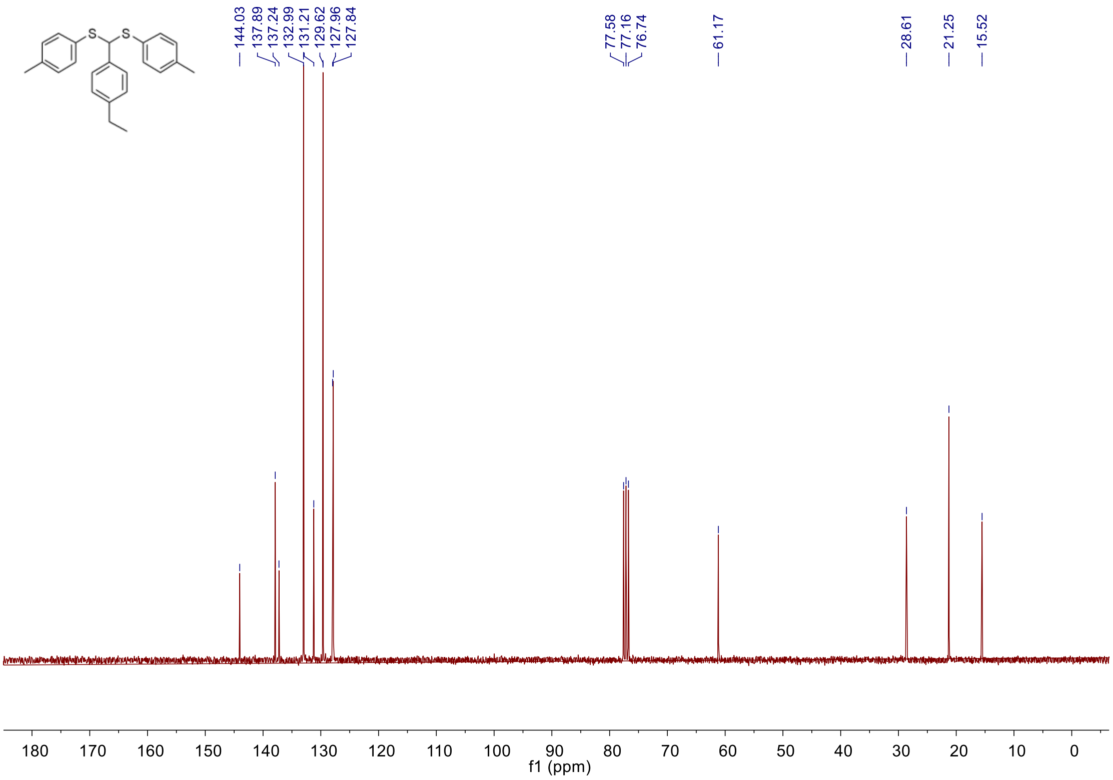

^{1}H NMR of ((4-methoxyphenyl)methylene)bis(p-tolylsulfane) (**3′f**)

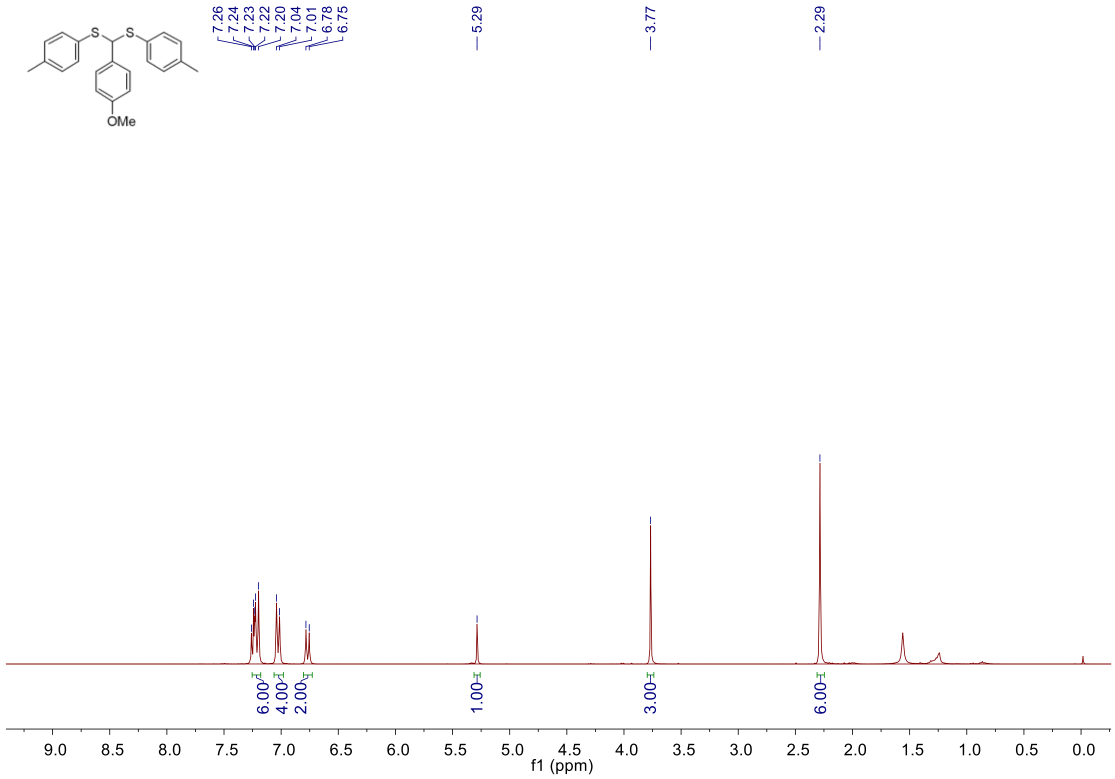

^{13}C NMR of ((4-methoxyphenyl)methylene)bis(p-tolylsulfane) (**3′f**)

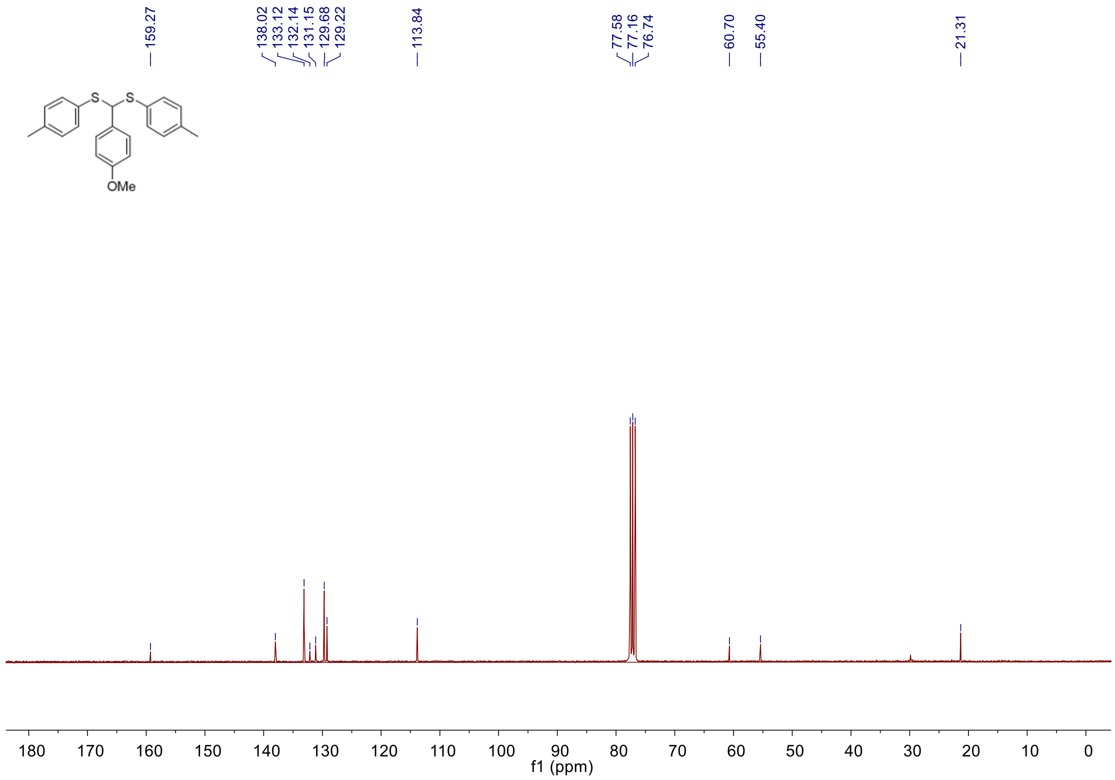

^{1}H NMR of ((4-fluorophenyl)methylene)bis(p-tolylsulfane) (**3′g**)

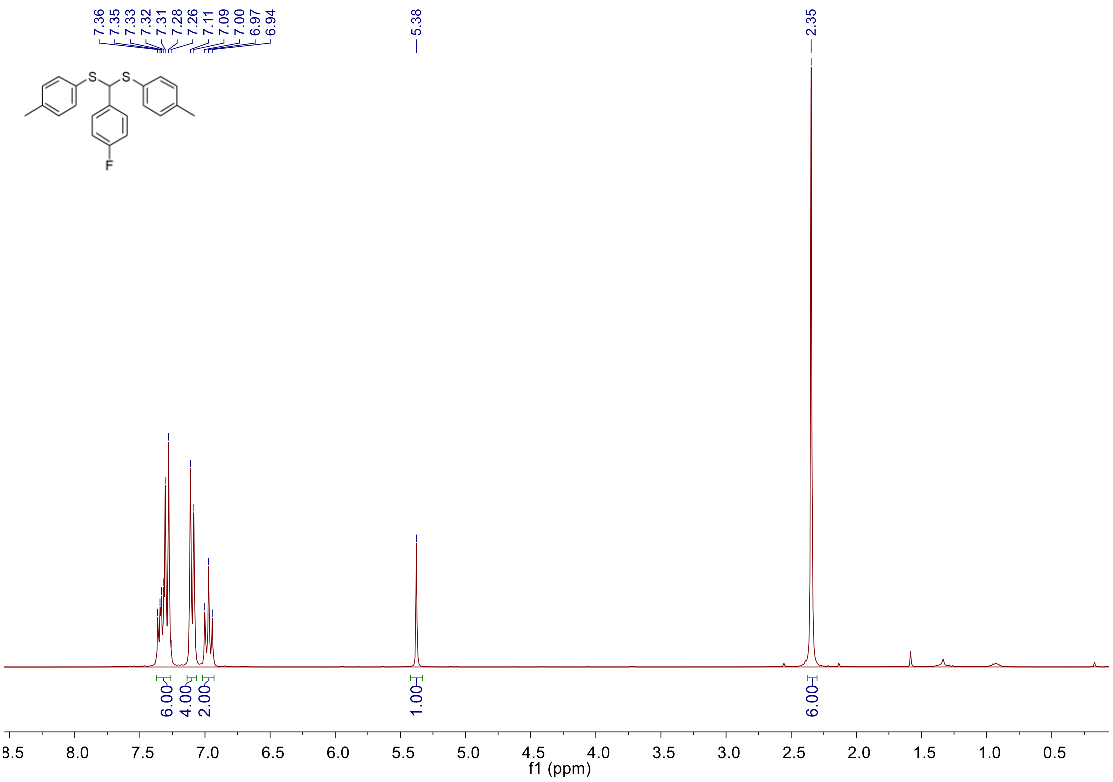

^{13}C NMR of ((4-fluorophenyl)methylene)bis(p-tolylsulfane) (**3′g**)

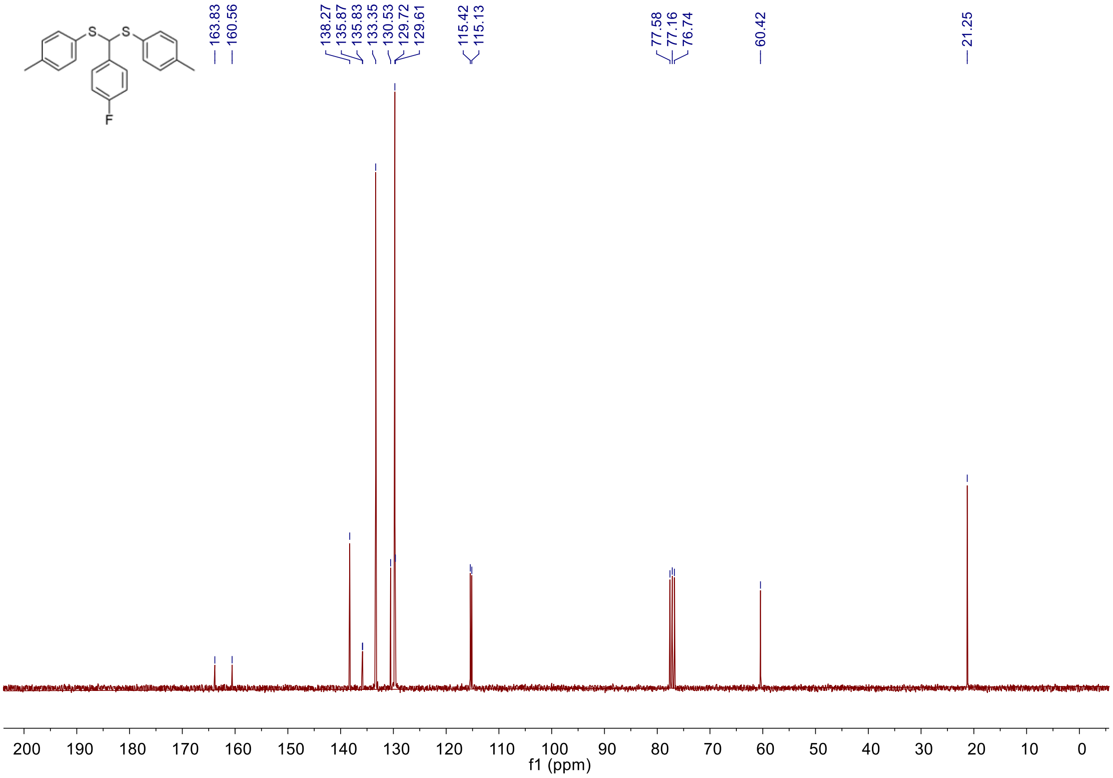

^{19}F NMR of ((4-fluorophenyl)methylene)bis(p-tolylsulfane) (**3′g**)

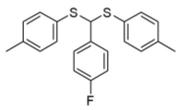

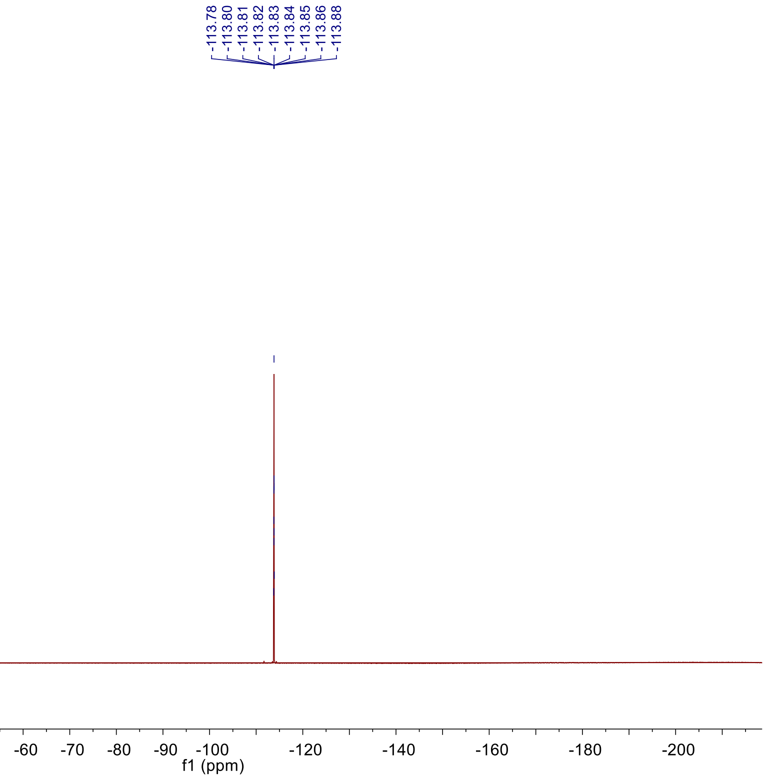

^{1}H NMR of ((4-chlorophenyl)methylene)bis(p-tolylsulfane) (**3′h**)

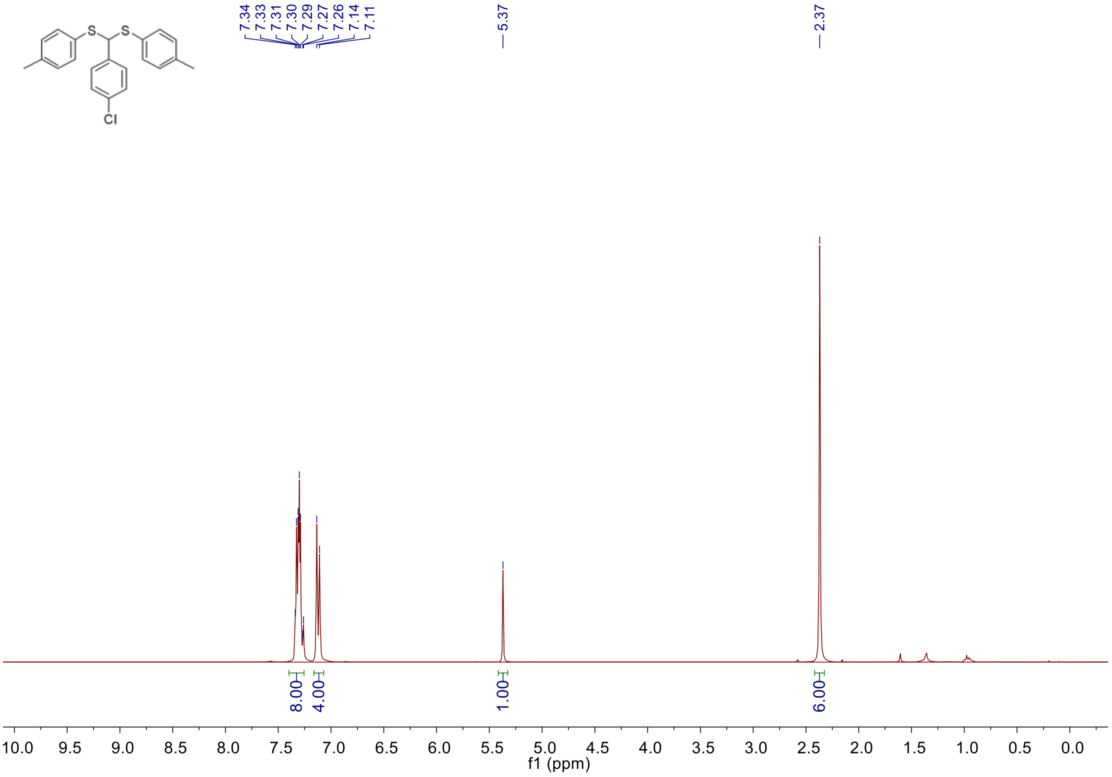

^{13}C NMR of ((4-chlorophenyl)methylene)bis(p-tolylsulfane) (**3′h**)

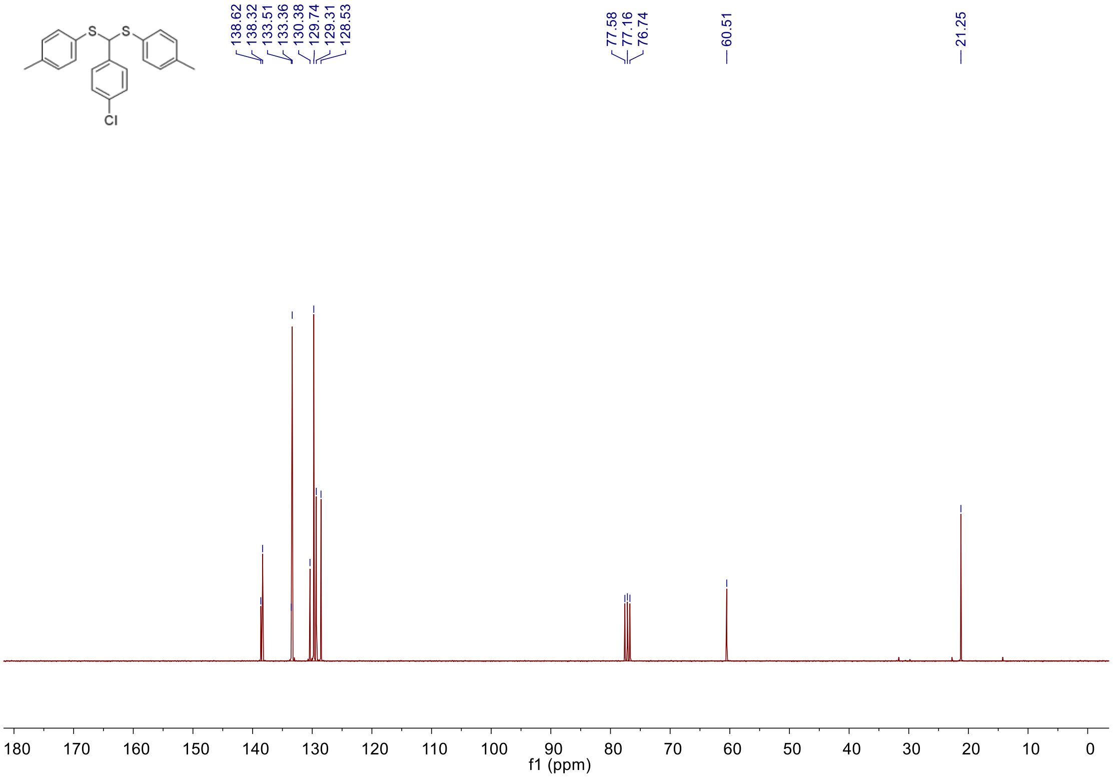

^{1}H NMR of ((4-bromophenyl)methylene)bis(p-tolylsulfane) (**3′i**)

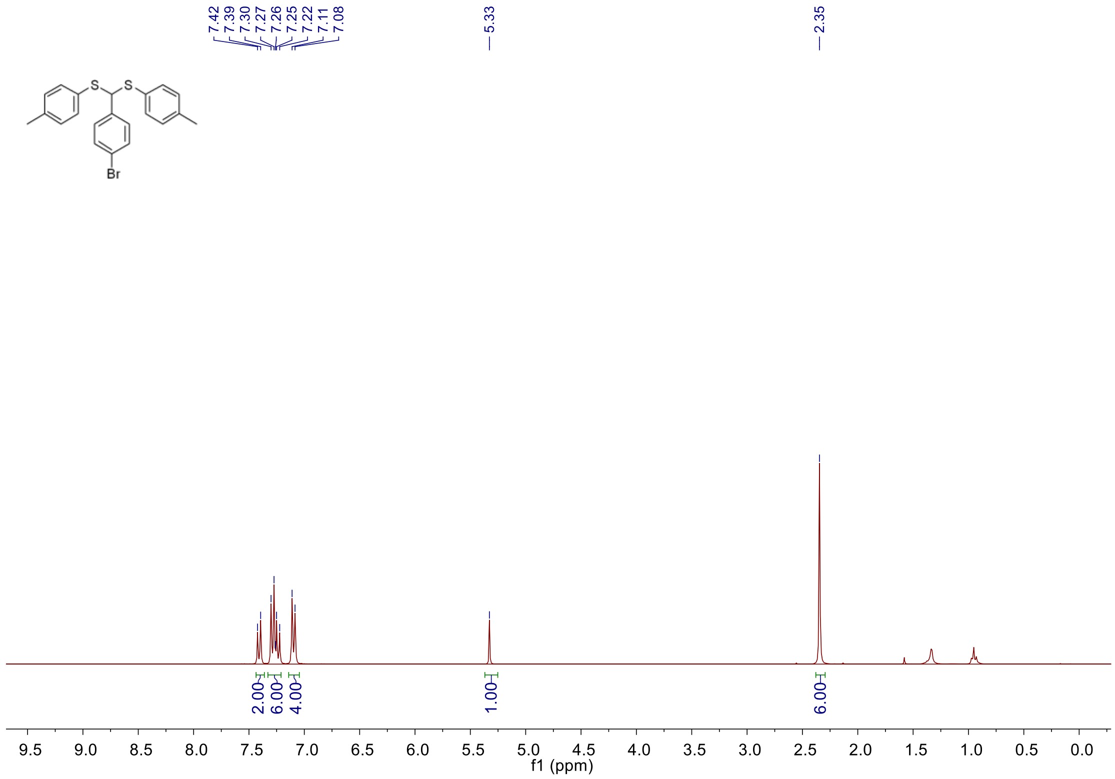

^{13}C NMR of ((4-bromophenyl)methylene)bis(p-tolylsulfane) (**3′i**)

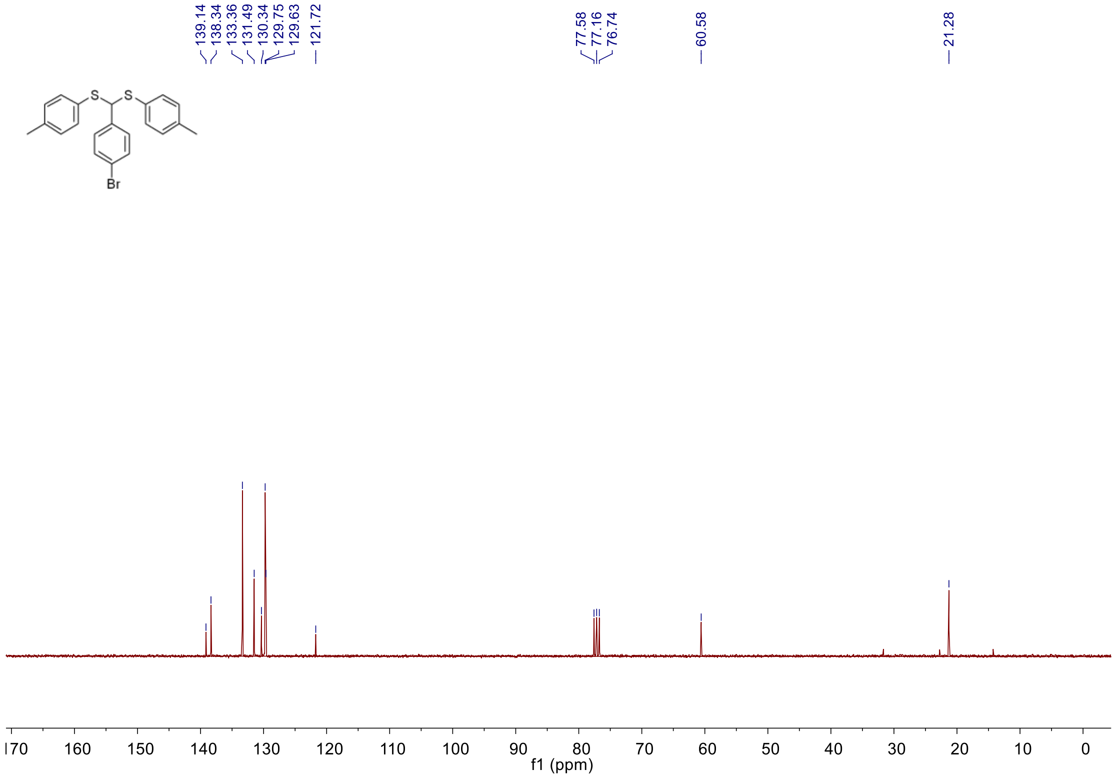

^{1}H NMR of 4-(bis(p-tolylthio)methyl)benzonitrile (**3′j**)

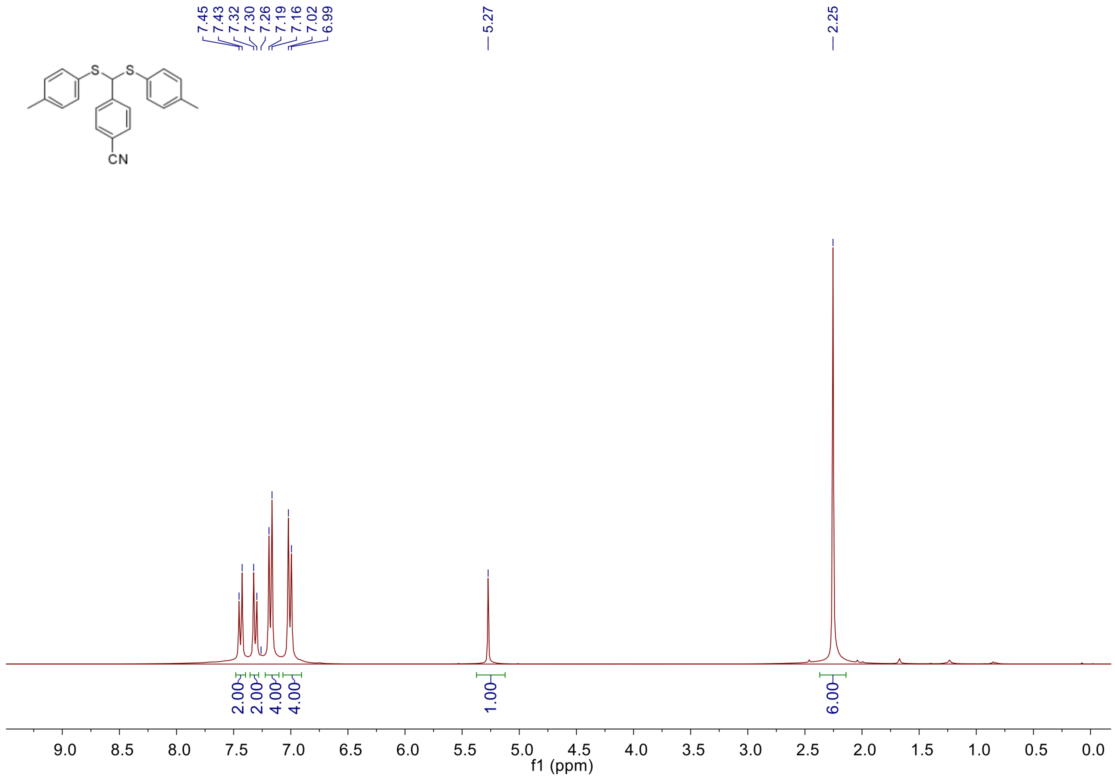

^{13}C NMR of 4-(bis(p-tolylthio)methyl)benzonitrile (**3′j**)

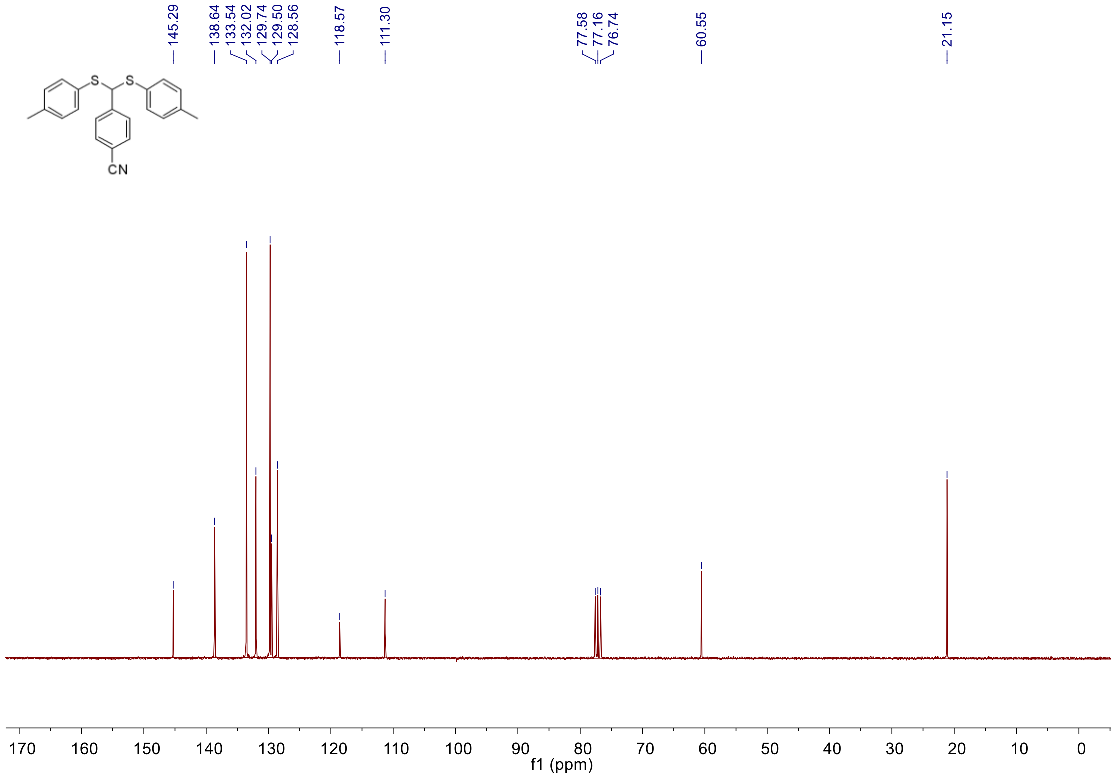

^{1}H NMR of 4-(bis(p-tolylthio)methyl)benzaldehyde (**3′k**)

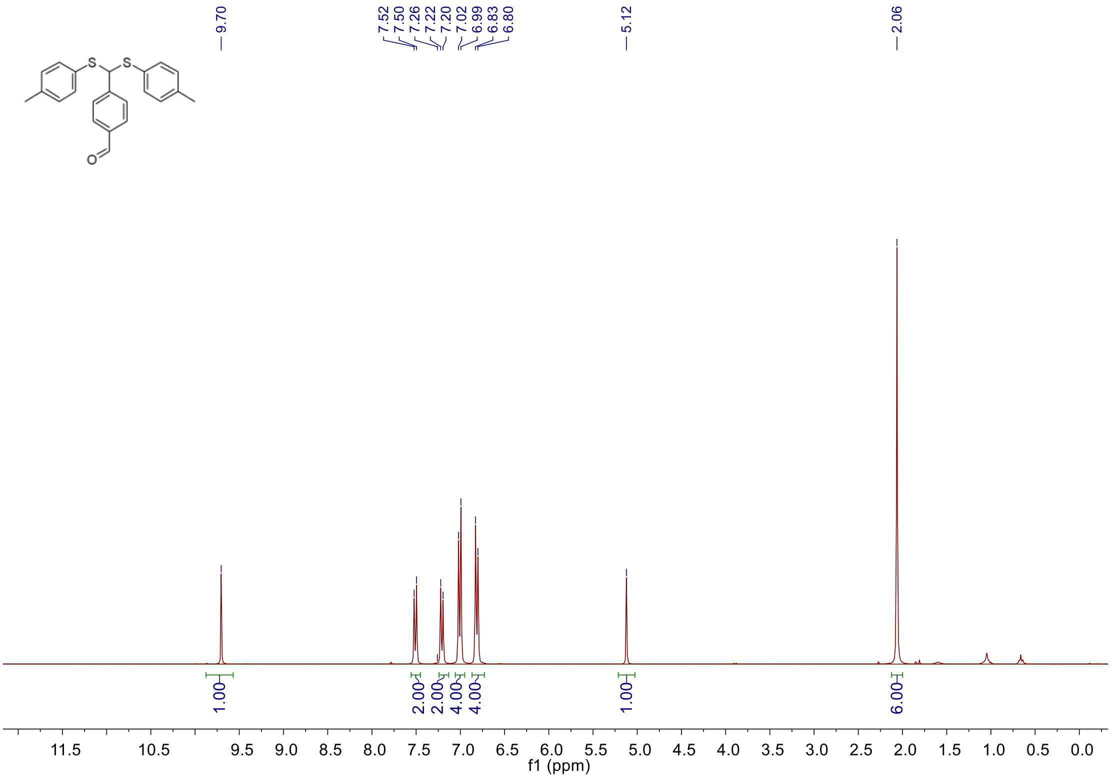

^{13}C NMR of 4-(bis(p-tolylthio)methyl)benzaldehyde (**3′k**)

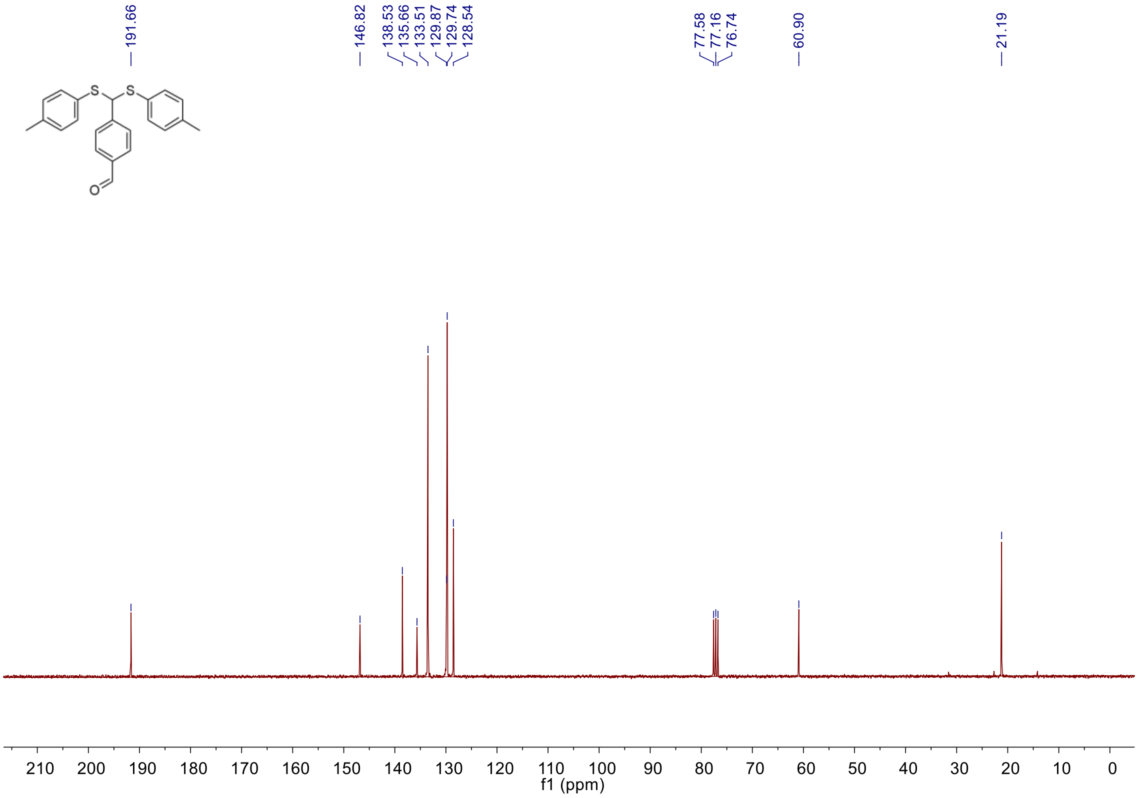

^{1}H NMR of ((4-chlorophenyl)methylene)bis((4-methoxyphenyl)sulfane) (**3′l**)

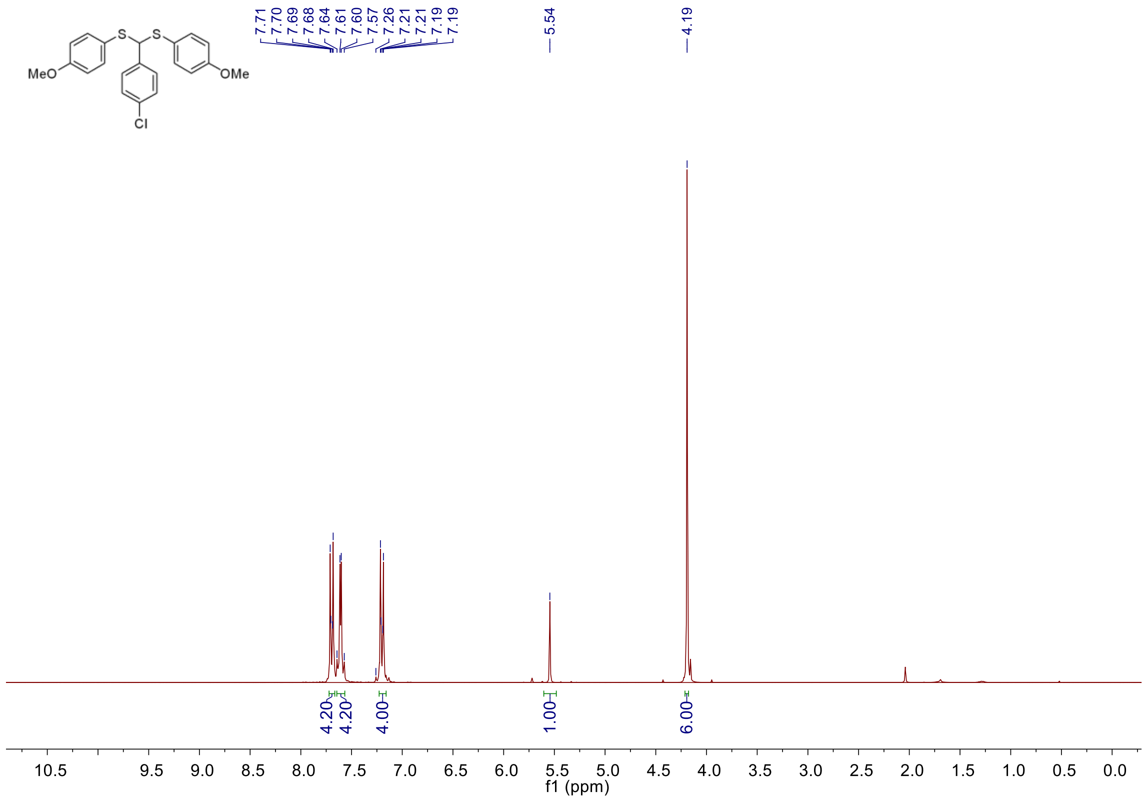

^{13}C NMR of ((4-chlorophenyl)methylene)bis((4-methoxyphenyl)sulfane) (**3′l**)

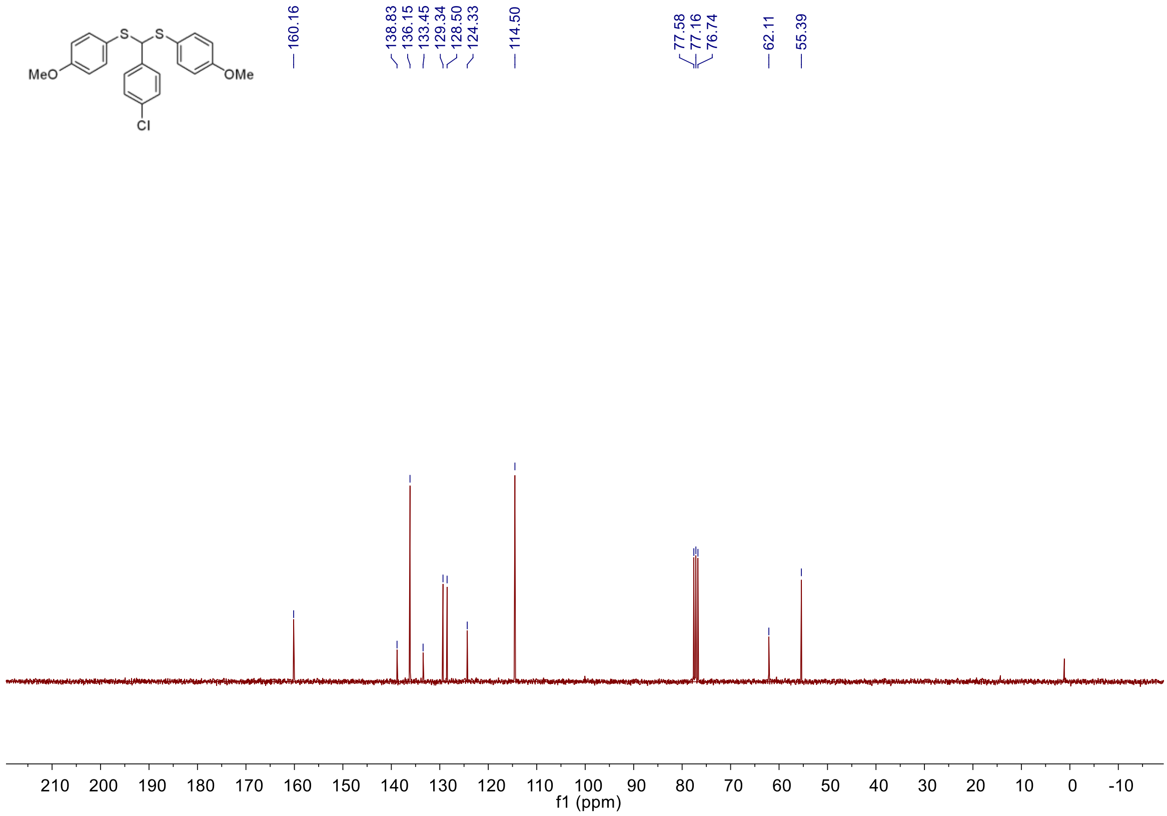

^{1}H NMR of dimethyl 4,4'-((phenylmethylene)bis(sulfanediyl))dibenzoate(**3′m**)

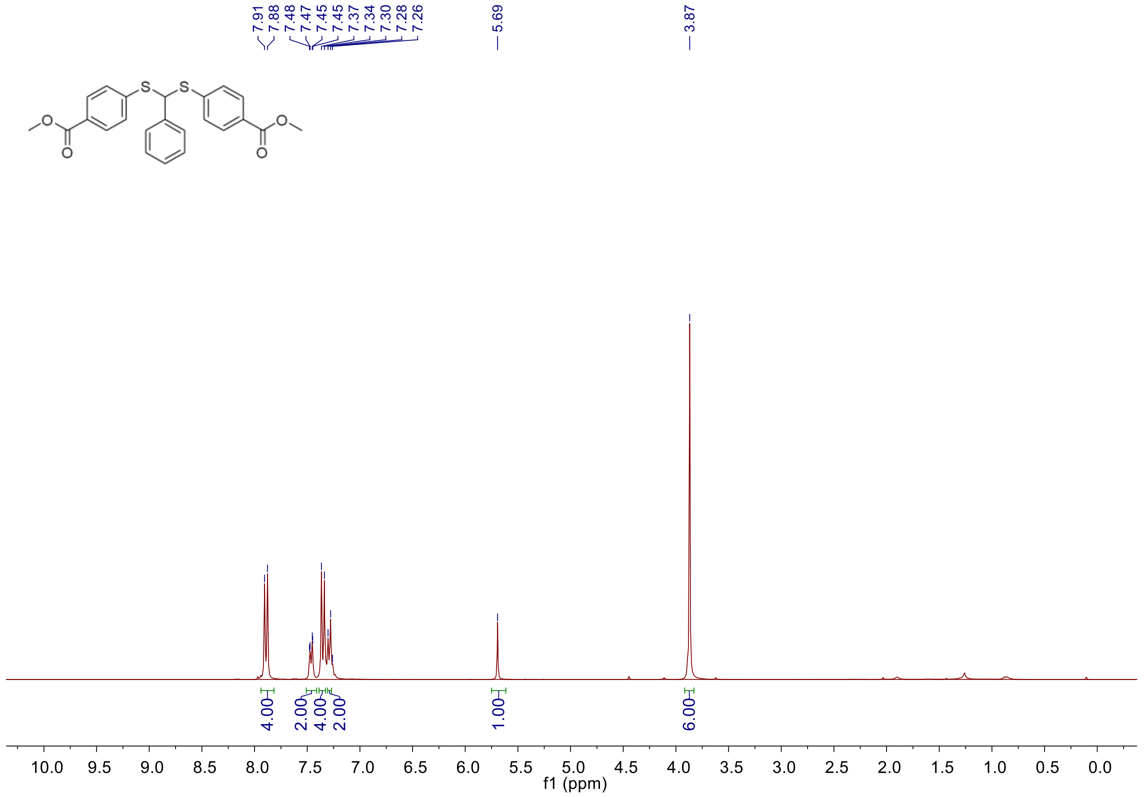

^{13}C NMR of dimethyl 4,4'-((phenylmethylene)bis(sulfanediyl))dibenzoate(**3′m**)

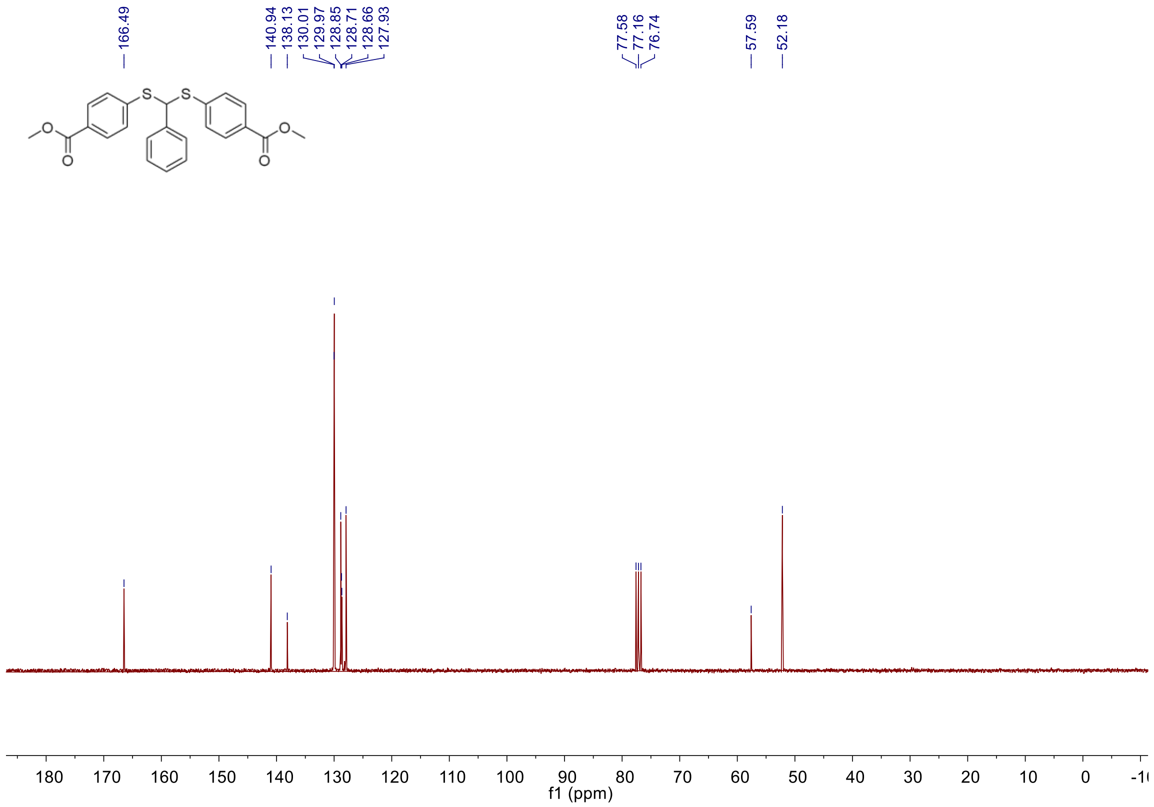

^{1}H NMR of 4-(bis((4-bromophenyl)thio)methyl)benzonitrile(**3′n**)

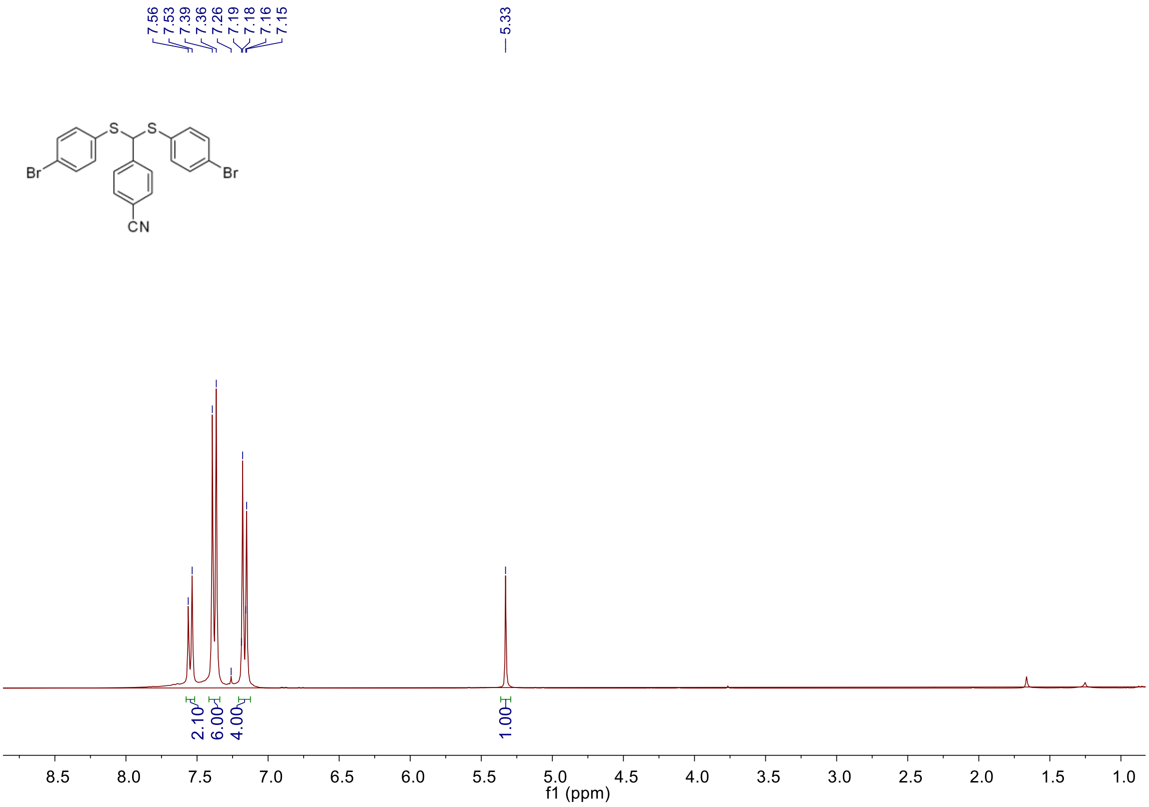

^{13}C NMR of 4-(bis((4-bromophenyl)thio)methyl)benzonitrile(**3′n**)

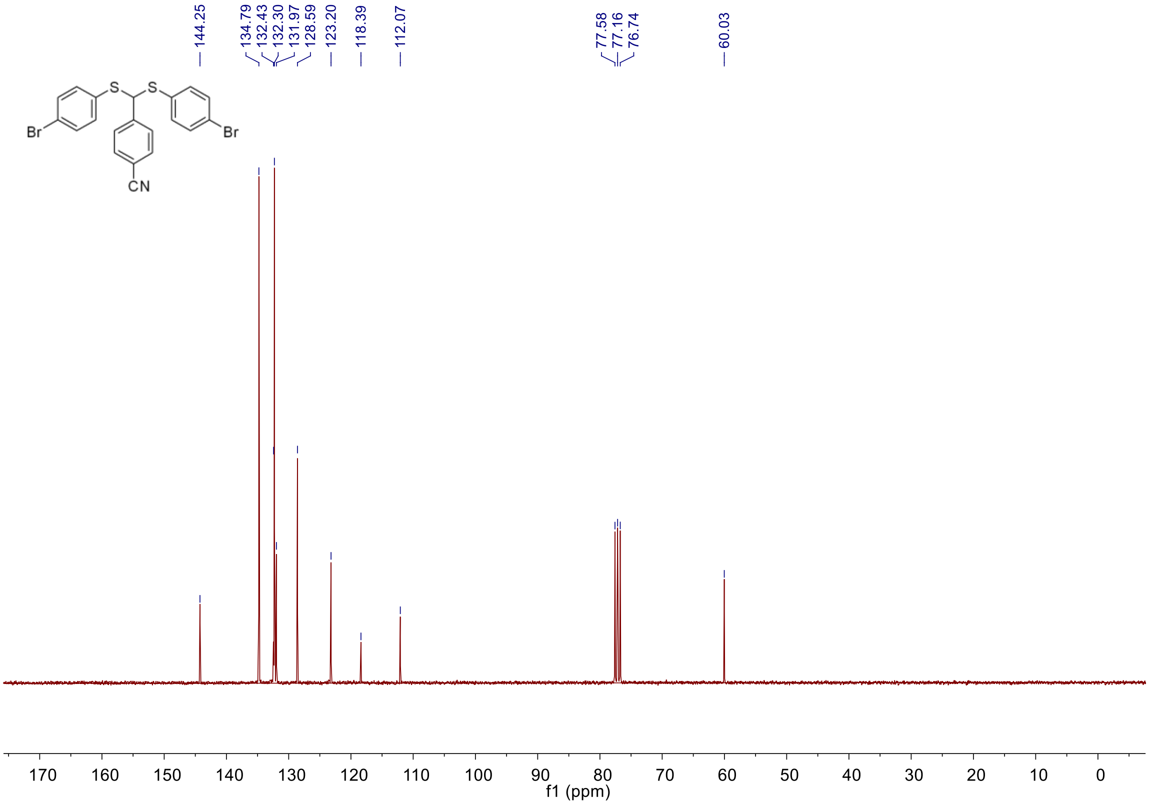

^{1}H NMR of (p-tolylmethylene)bis((4-nitrophenyl)sulfane) (**3′o**)

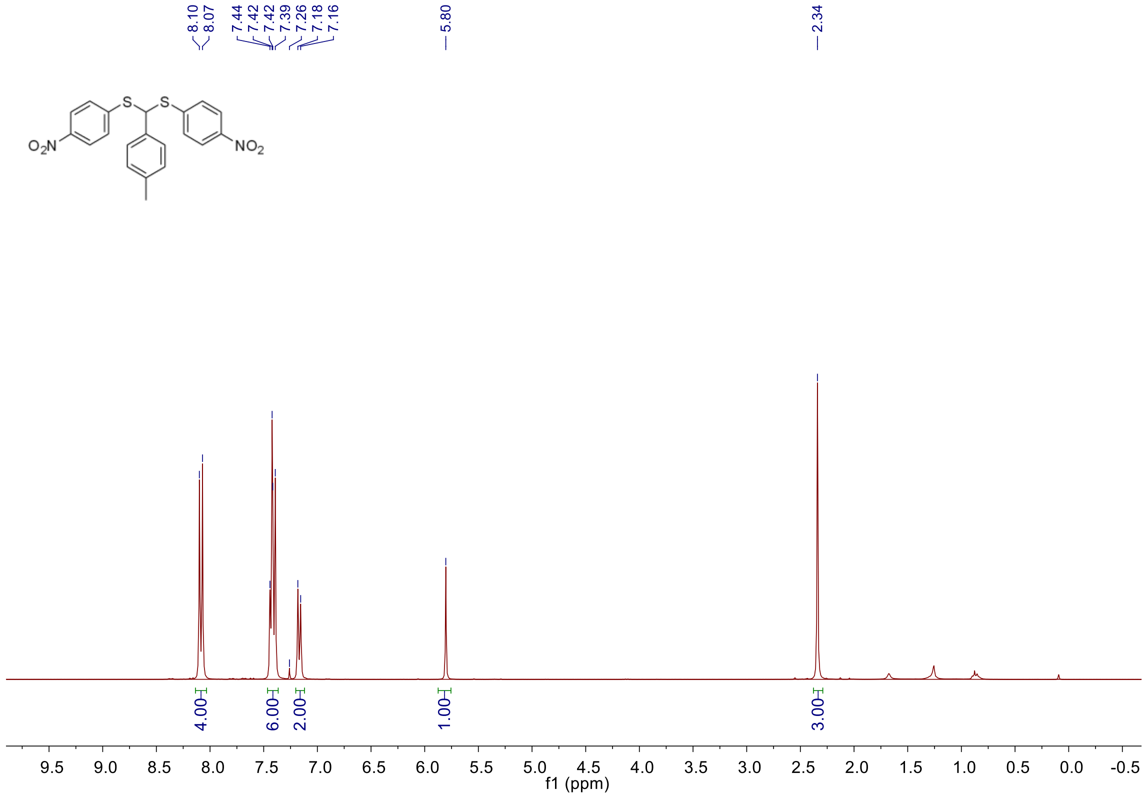

^{13}C NMR of (p-tolylmethylene)bis((4-nitrophenyl)sulfane) (**3′o**)

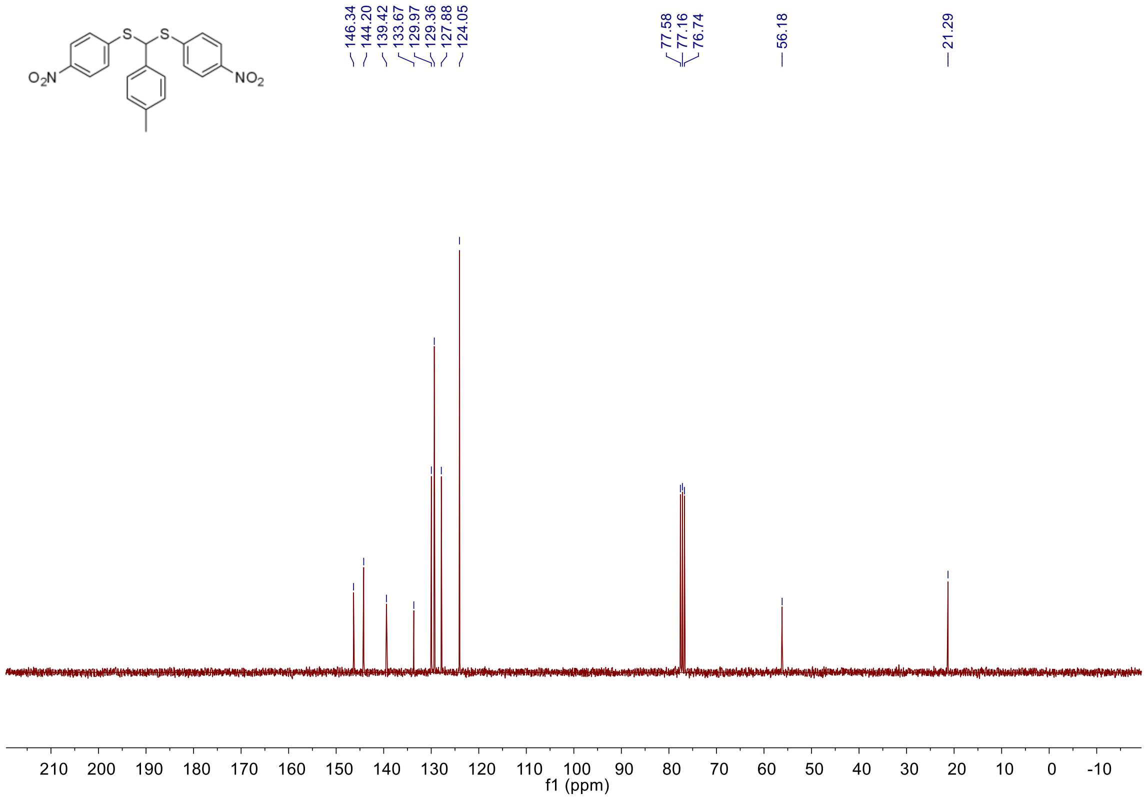

^{1}H NMR of (p-tolylmethylene)bis(naphthalen-2-ylsulfane) (**3′p**)

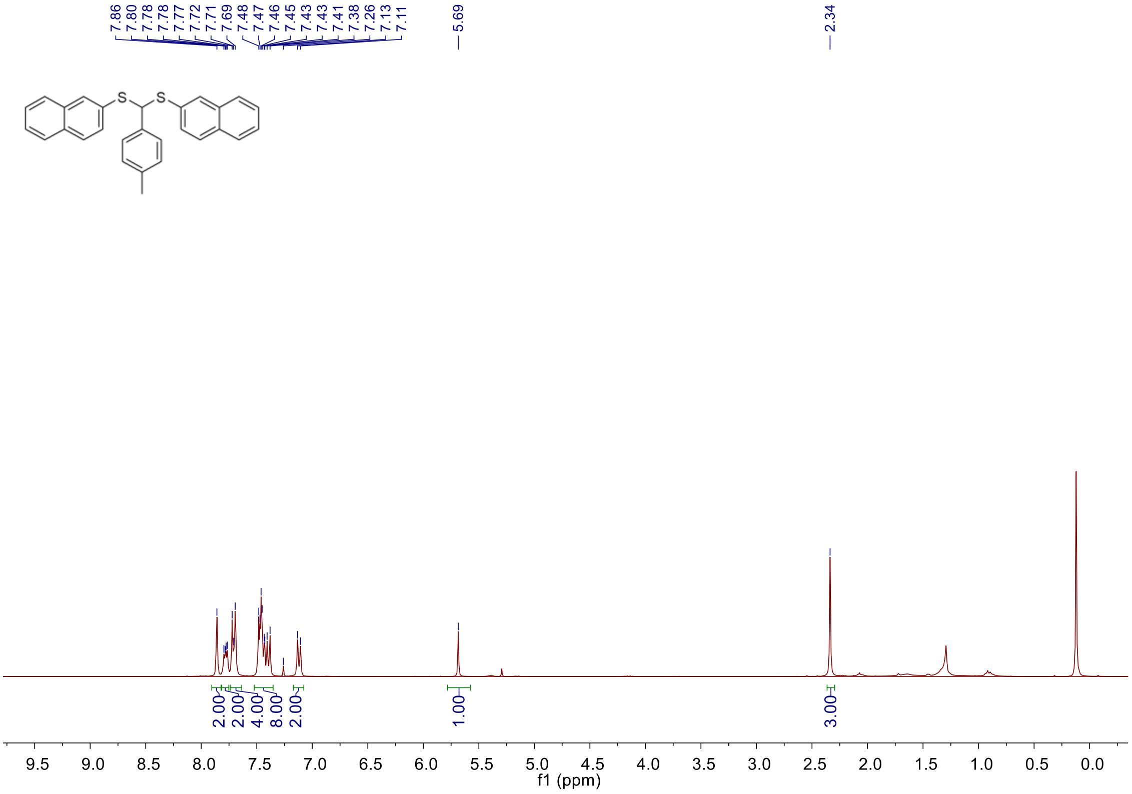

^{13}C NMR of (p-tolylmethylene)bis(naphthalen-2-ylsulfane) (**3′p**)

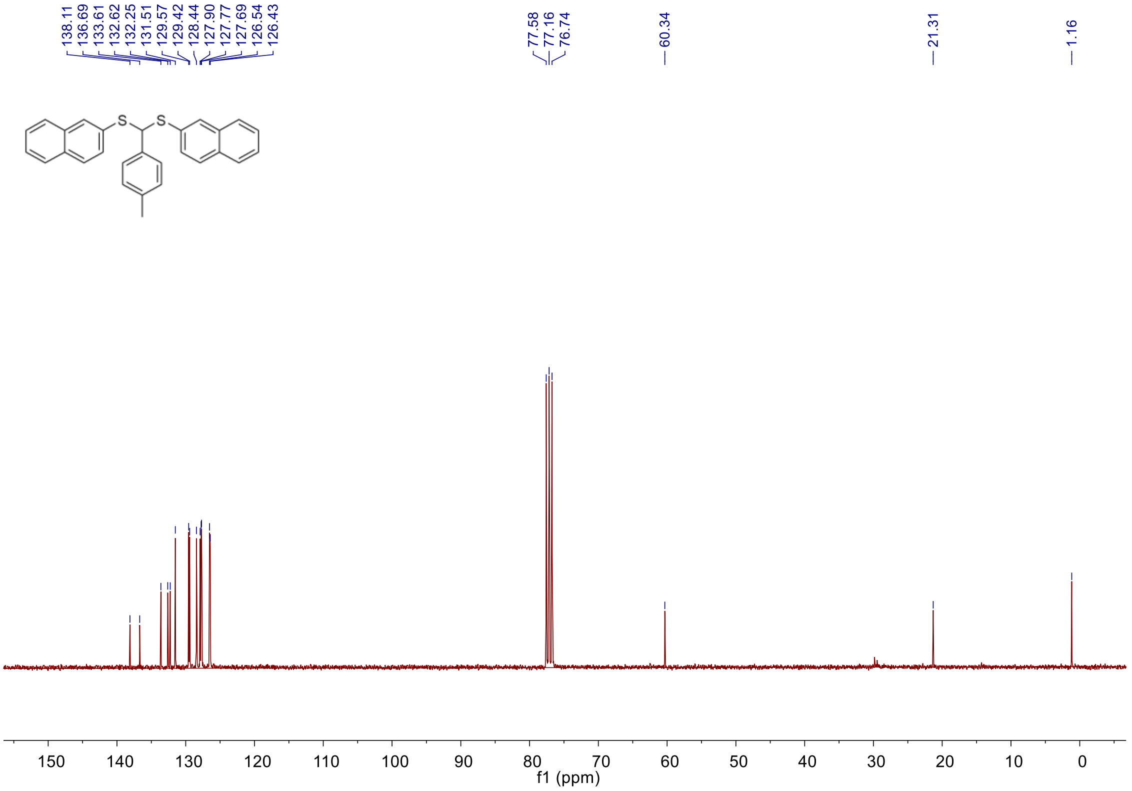

^{1}H NMR of (phenylmethylene)bis(benzylsulfane) (**3′q**)

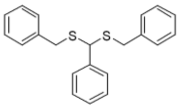

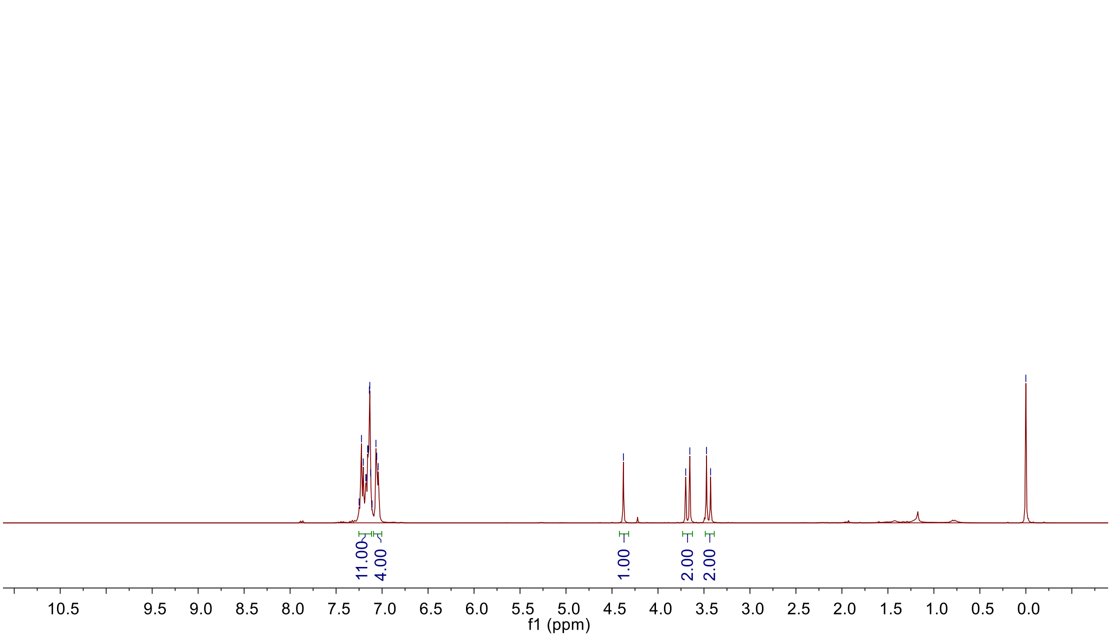

^{13}C NMR of (phenylmethylene)bis(benzylsulfane) (**3′q**)

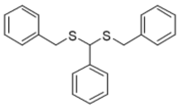

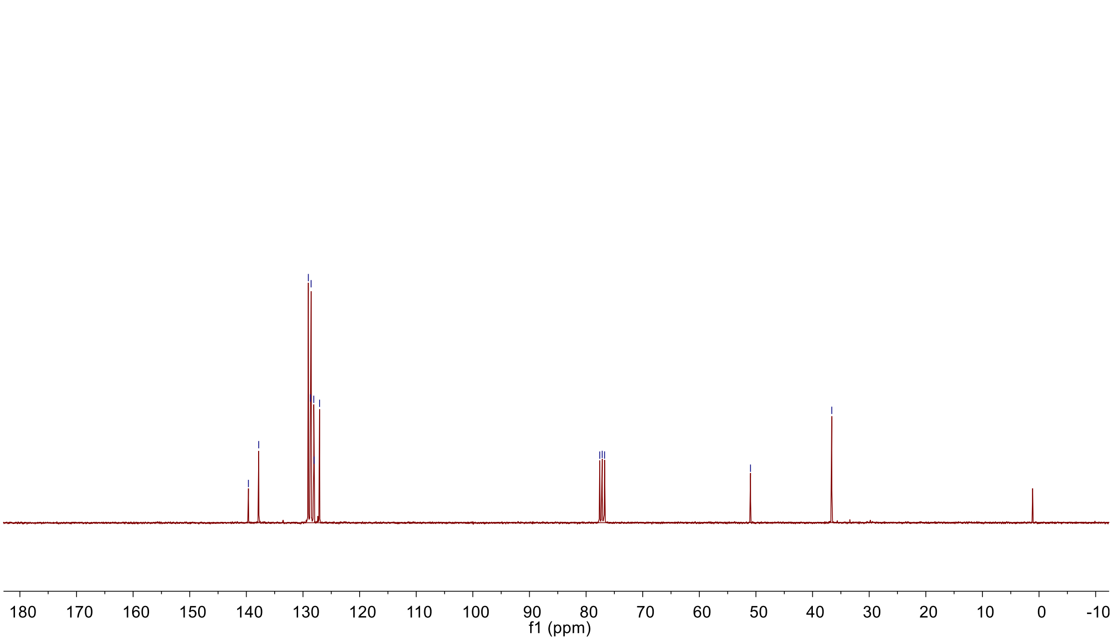

^{1}H NMR of 4-methyl-4-(p-tolylthio)pentan-2-one (**4a**).

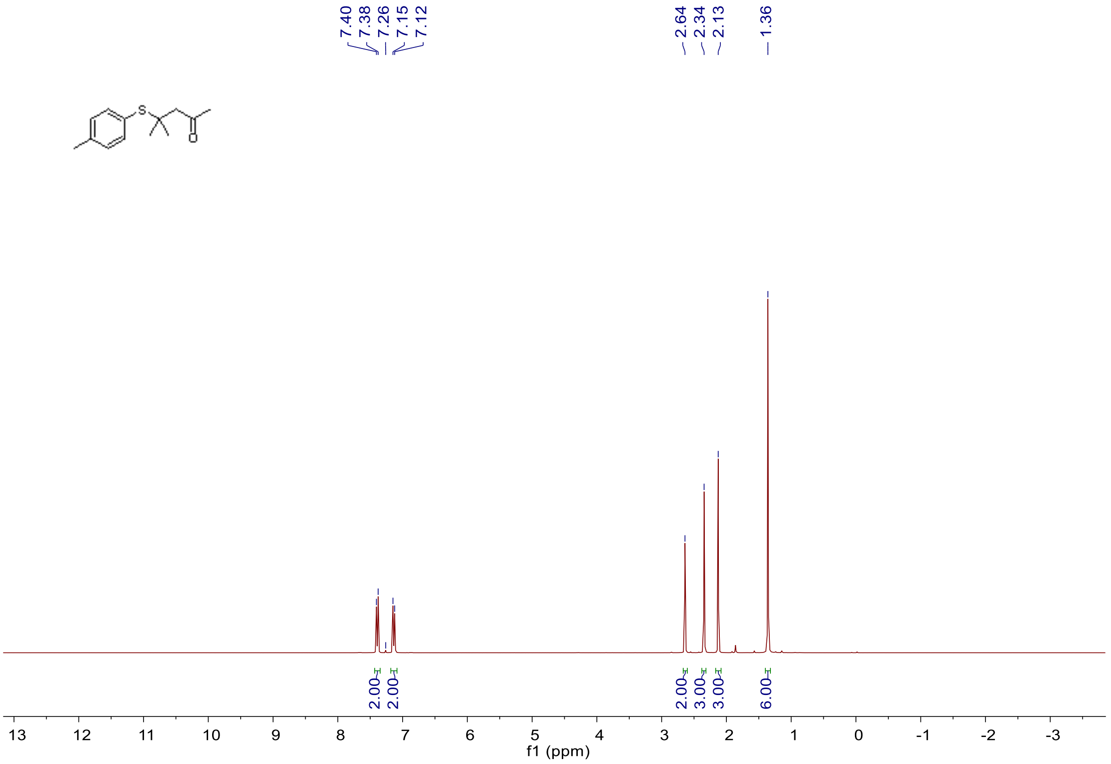

^{13}C NMR of 4-methyl-4-(p-tolylthio)pentan-2-one (**4a**).

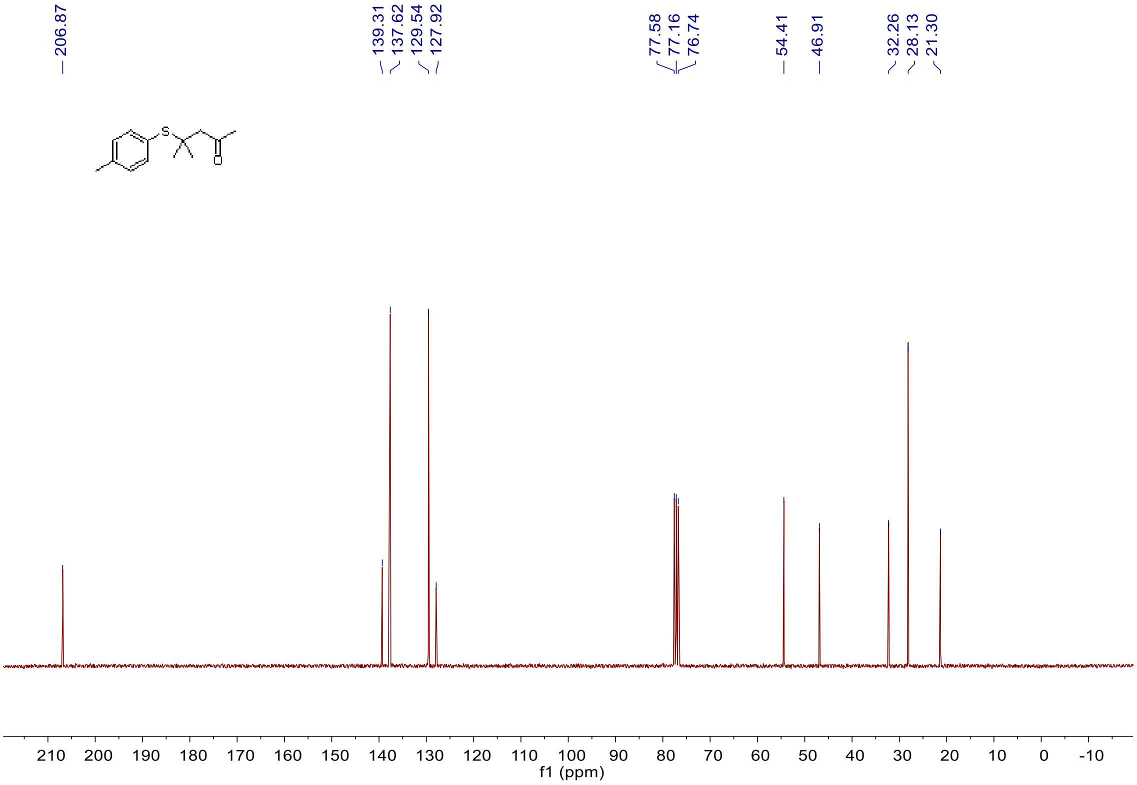

^{1}H NMR of 4-methyl-4-(m-tolylthio)pentan-2-one (**4b**).

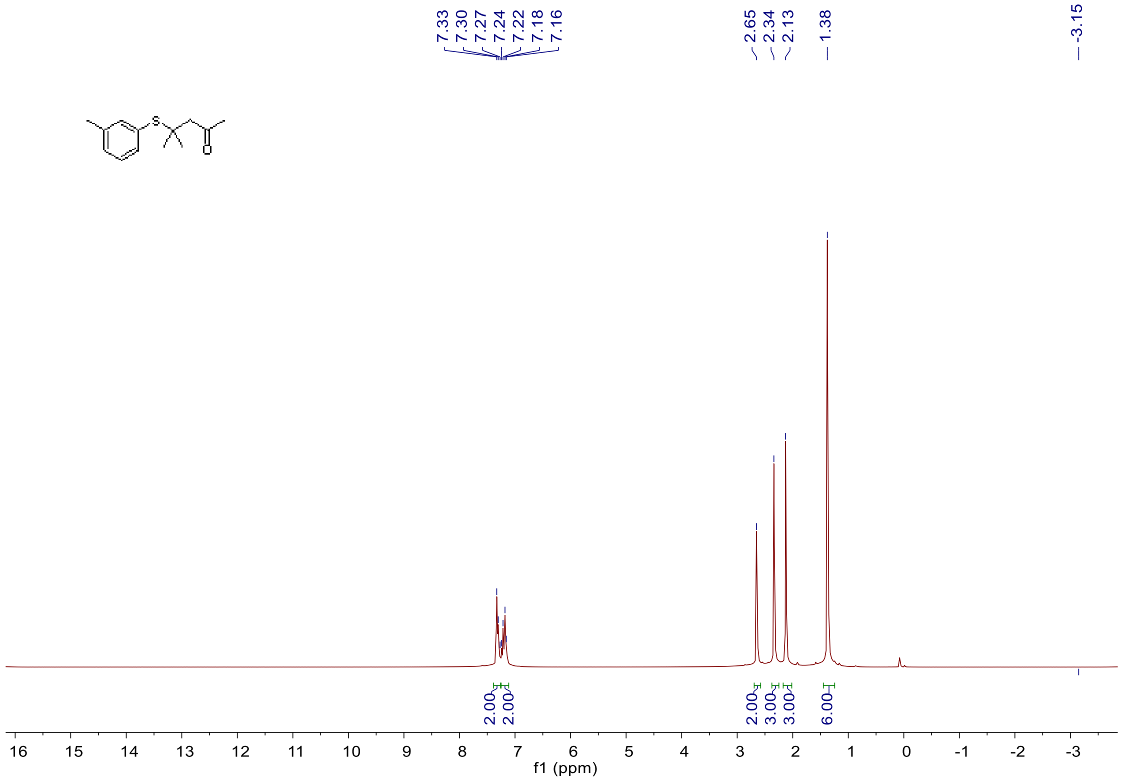

^{13}C NMR of 4-methyl-4-(m-tolylthio)pentan-2-one (**4b**).

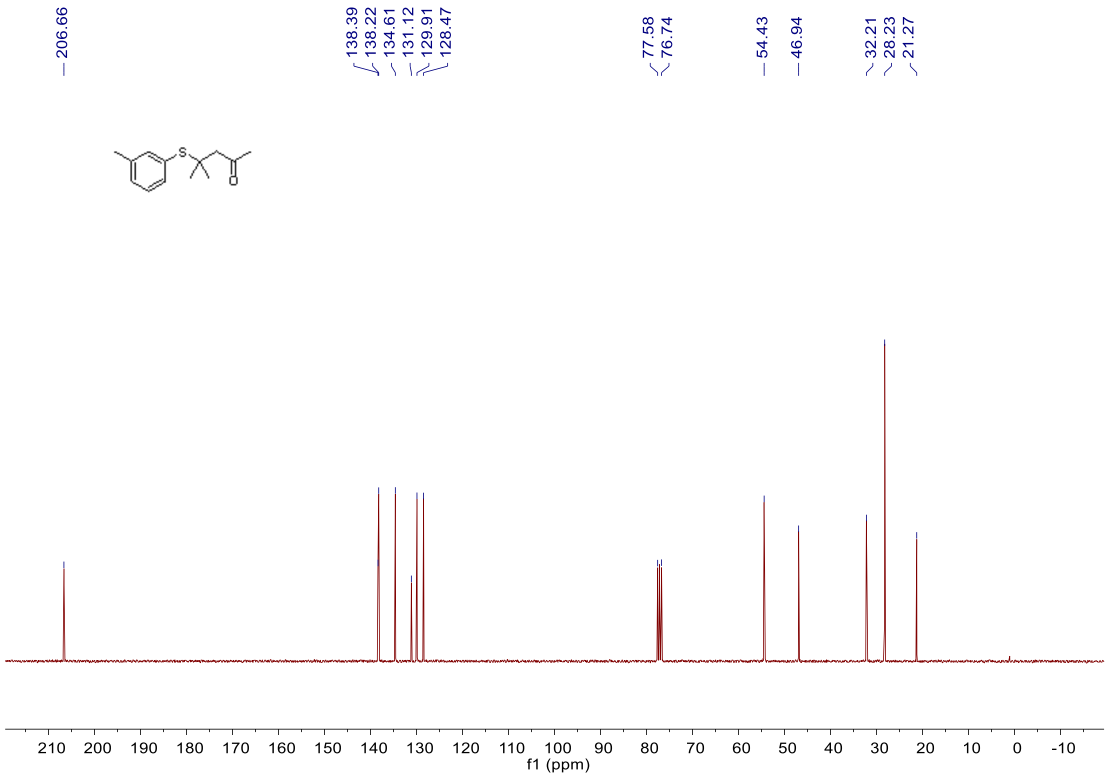

^{1}H NMR of 4-methyl-4-(o-tolylthio)pentan-2-one (**4c**).

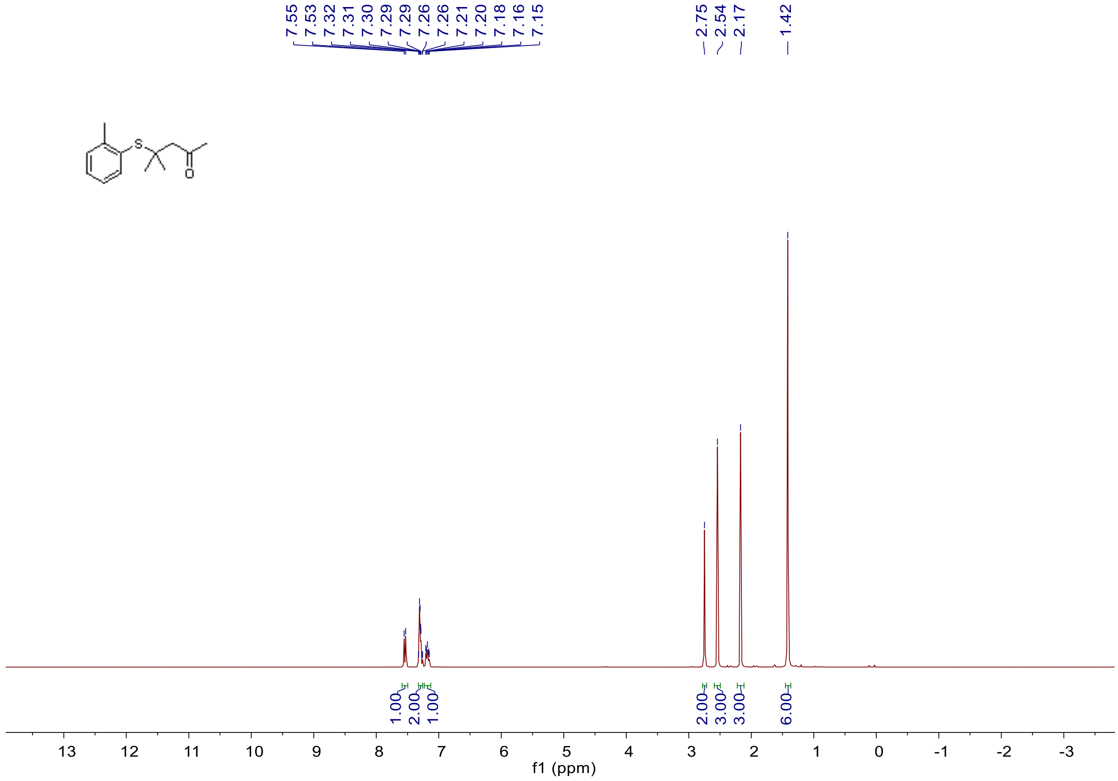

^{13}C NMR of 4-methyl-4-(o-tolylthio)pentan-2-one (**4c**).

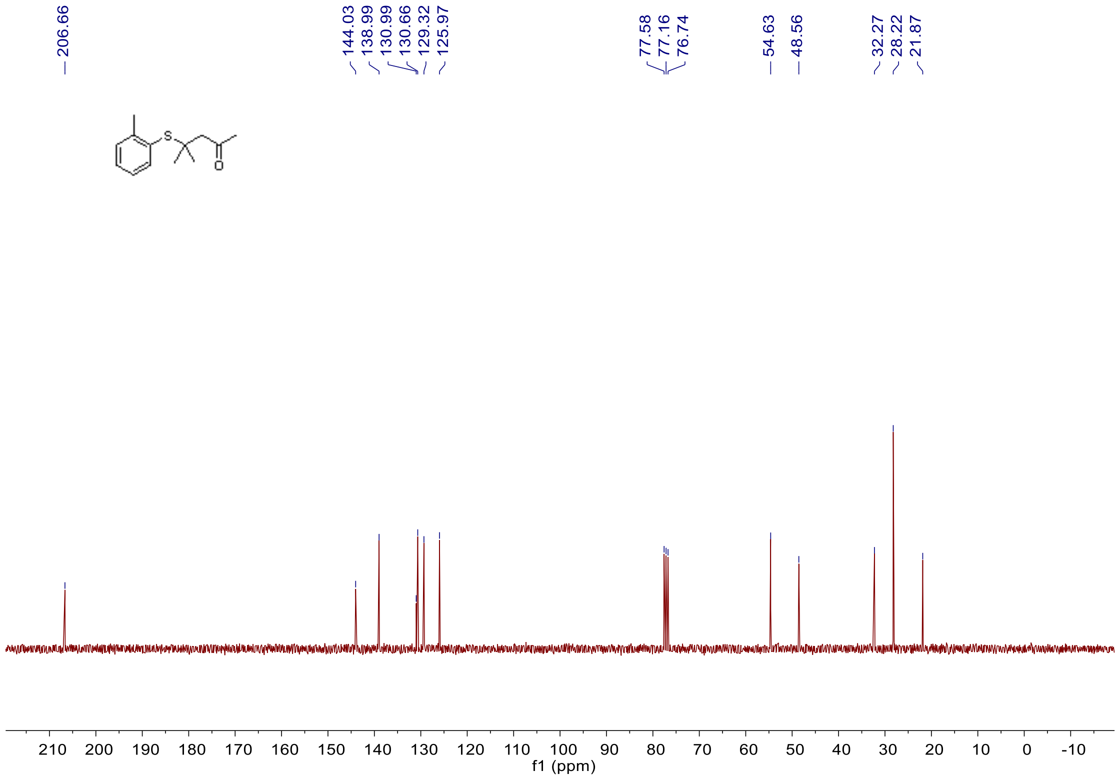

^{1}H NMR of 4-((4-isopropylphenyl)thio)-4-methylpentan-2-one (**4d**).

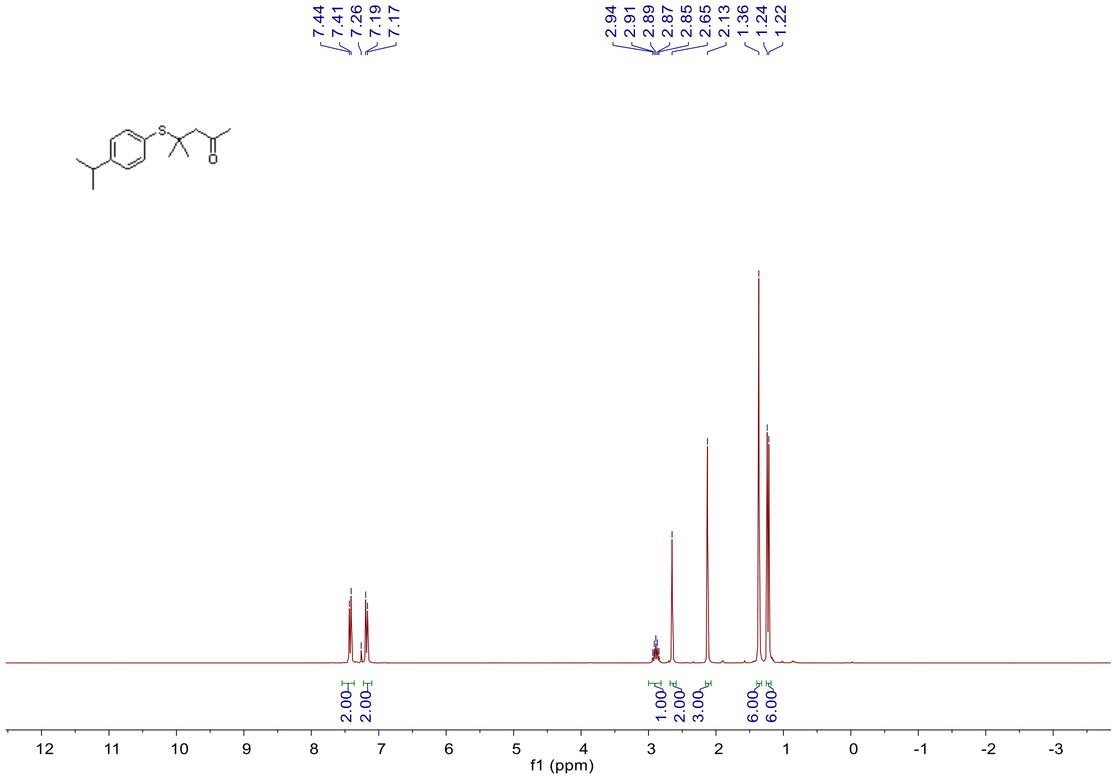

^{13}C NMR of 4-((4-isopropylphenyl)thio)-4-methylpentan-2-one (**4d**).

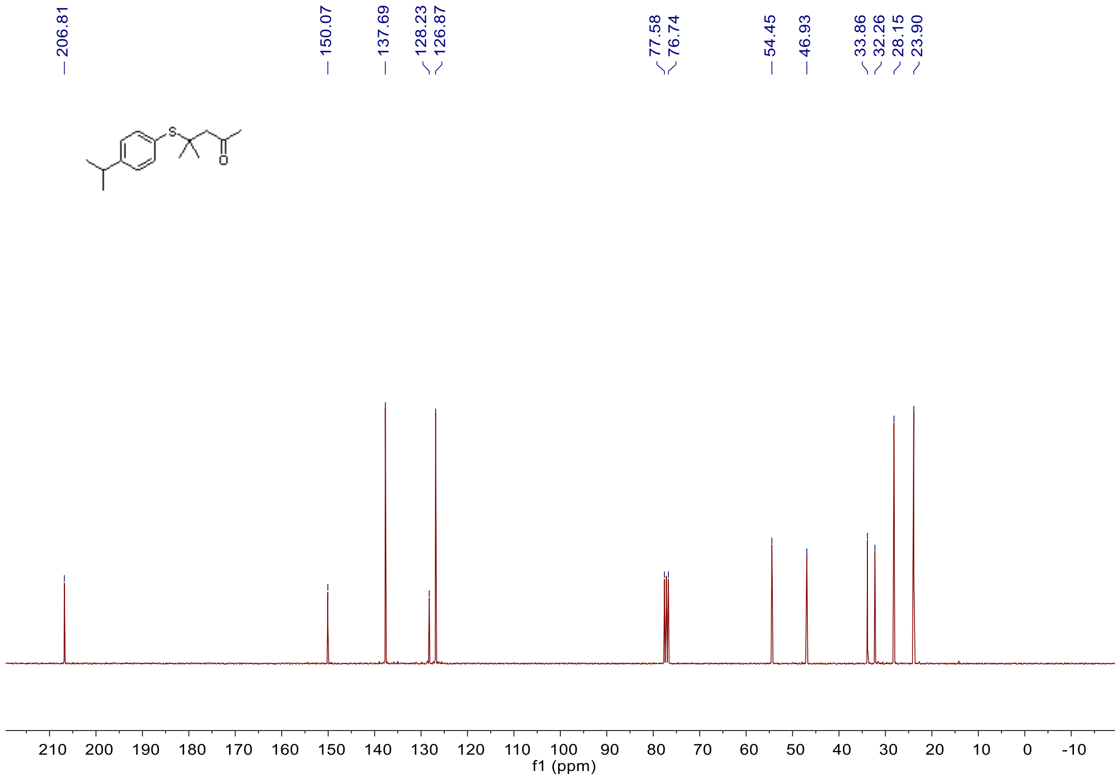

^{1}H NMR of 4-((2,4-dimethylphenyl)thio)-4-methylpentan-2-one (**4e**)

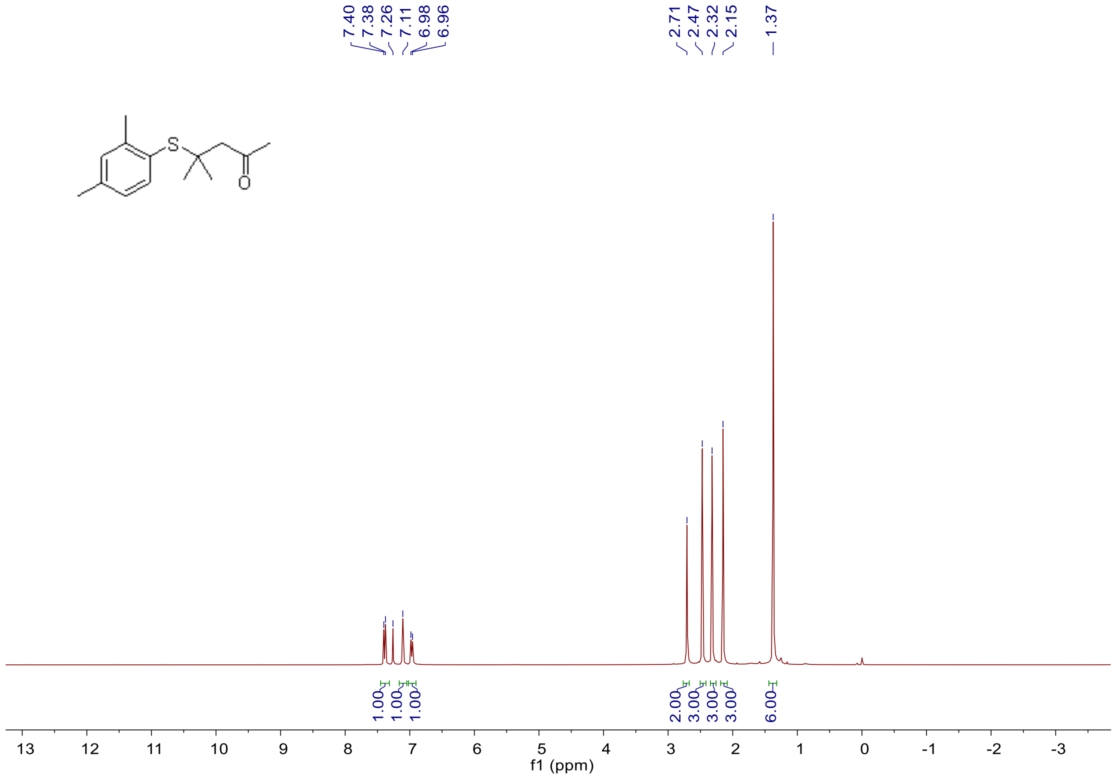

^{13}C NMR of 4-((2,4-dimethylphenyl)thio)-4-methylpentan-2-one (**4e**)

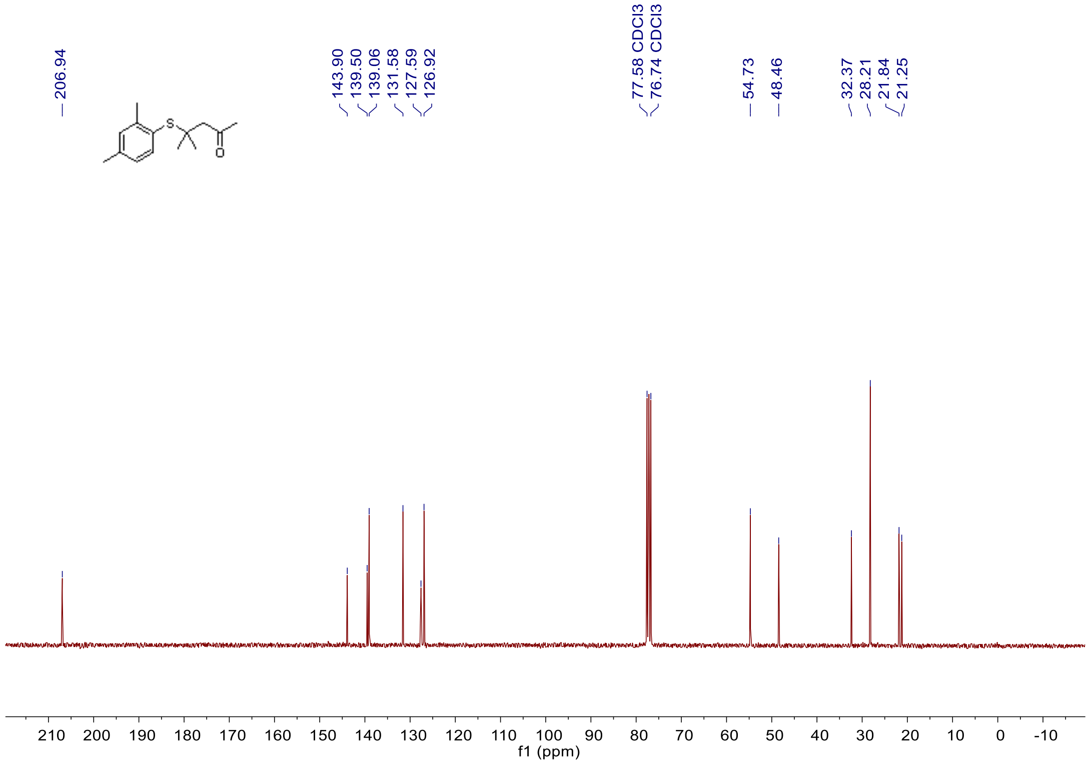

^{1}H NMR of 4-((4-methoxyphenyl)thio)-4-methylpentan-2-one (**4f**)

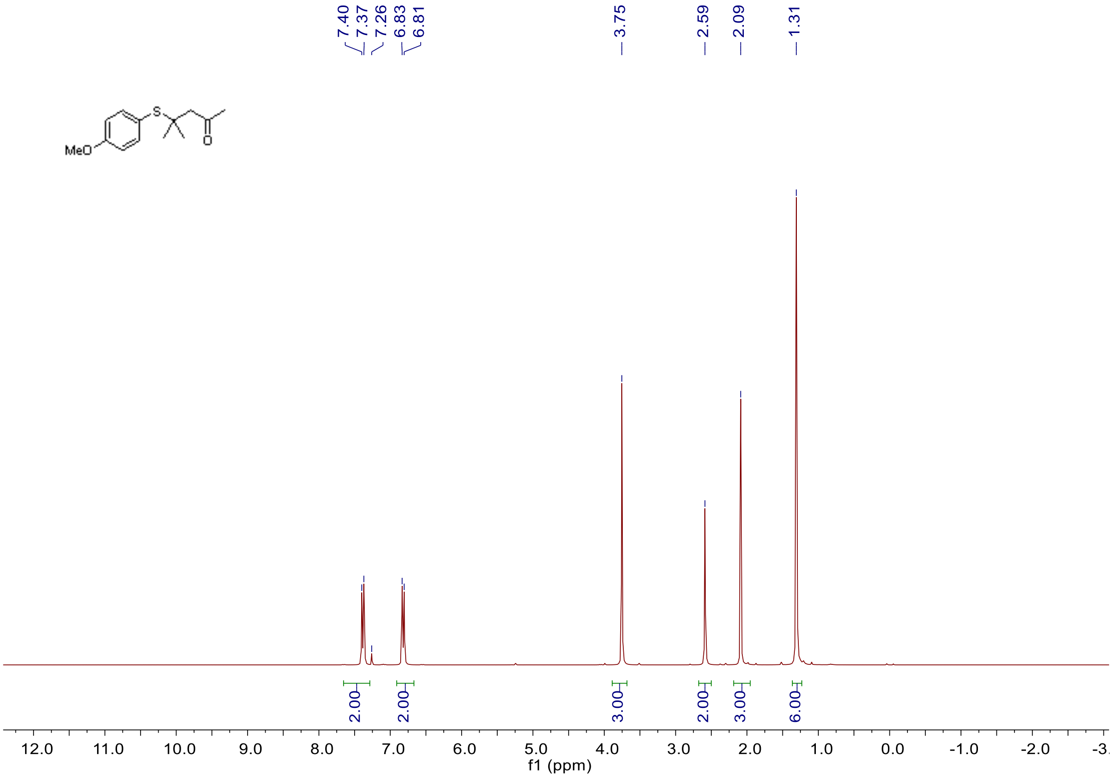

^{13}C NMR of 4-((4-methoxyphenyl)thio)-4-methylpentan-2-one (**4f**)

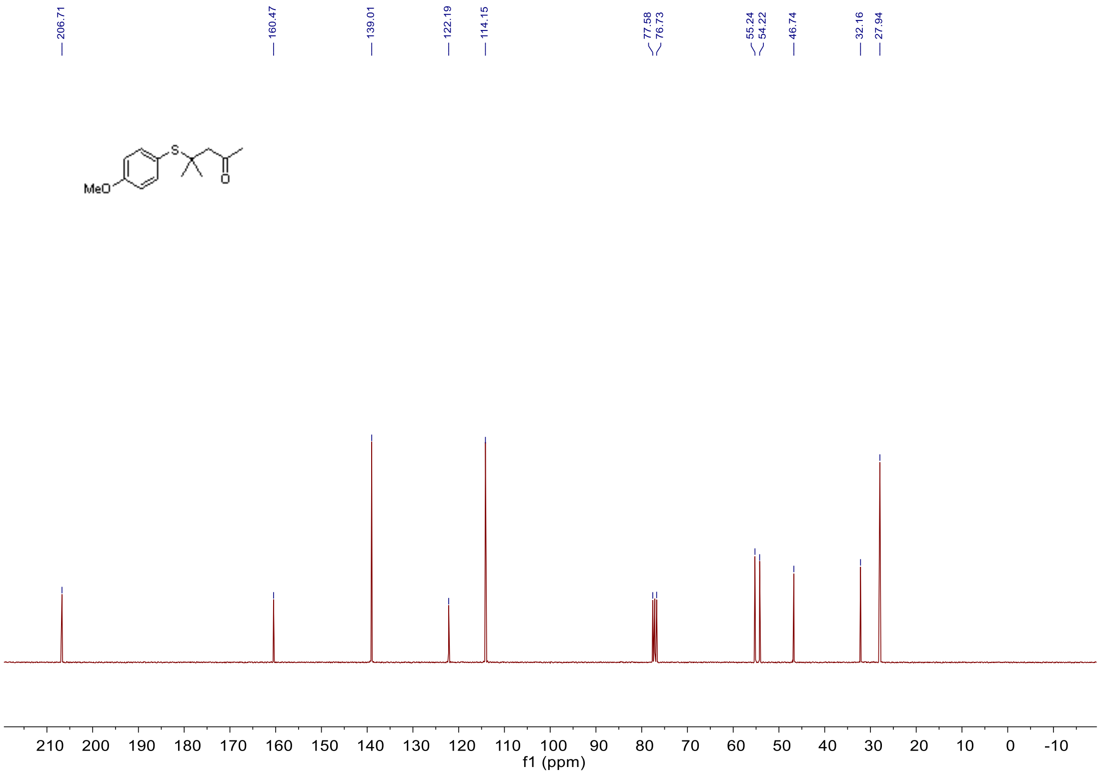

^{1}H NMR of 4-((4-fluorophenyl)thio)-4-methylpentan-2-one (**4g**)

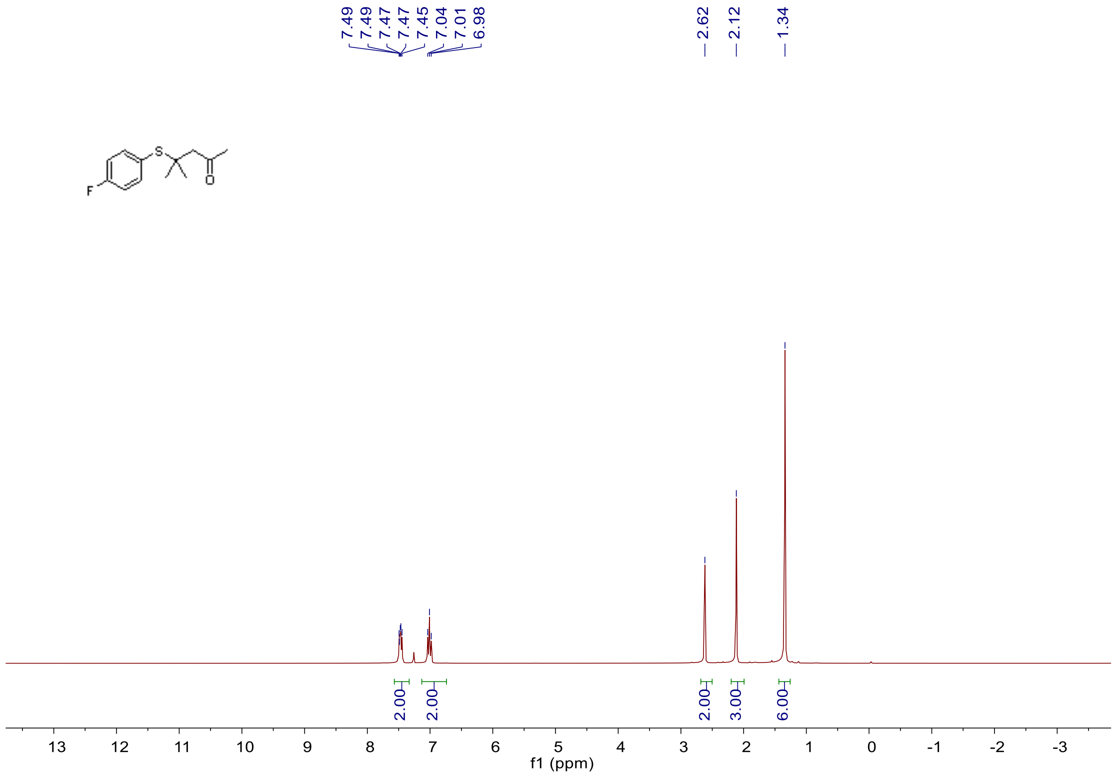

^{13}C NMR of 4-((4-fluorophenyl)thio)-4-methylpentan-2-one (**4g**)

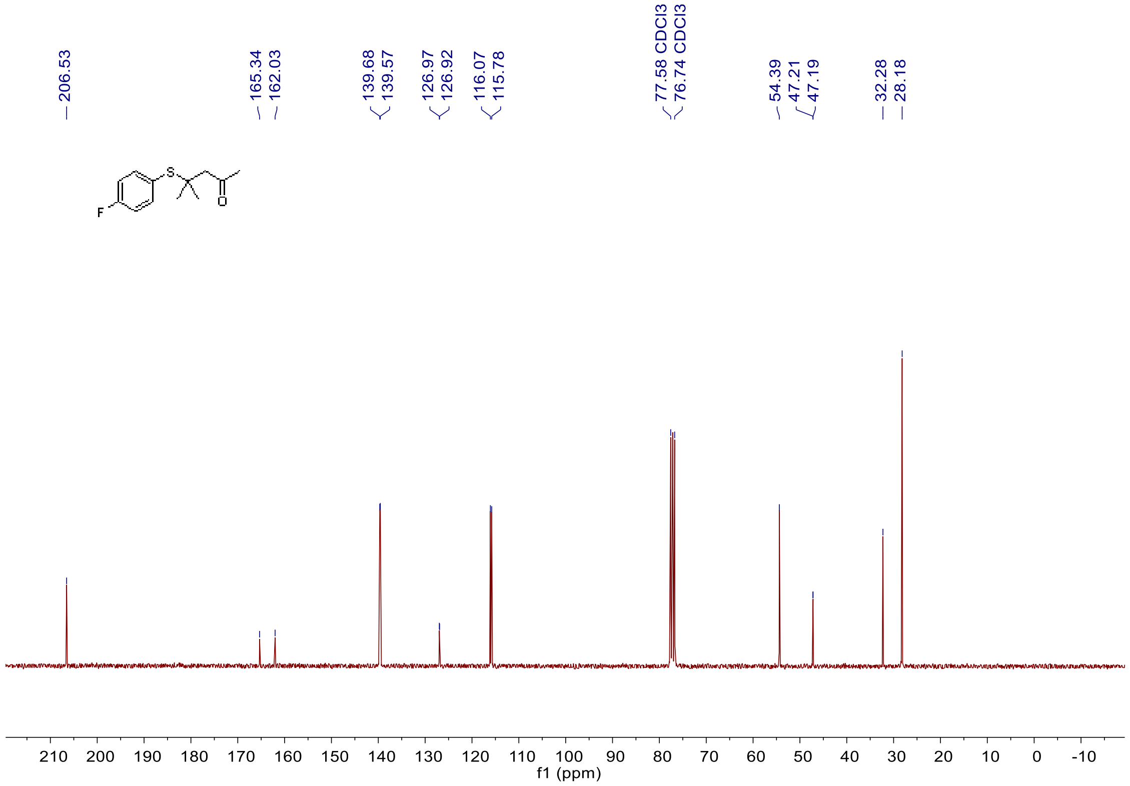

^{19}F NMR of 4-((4-fluorophenyl)thio)-4-methylpentan-2-one (**4g**)

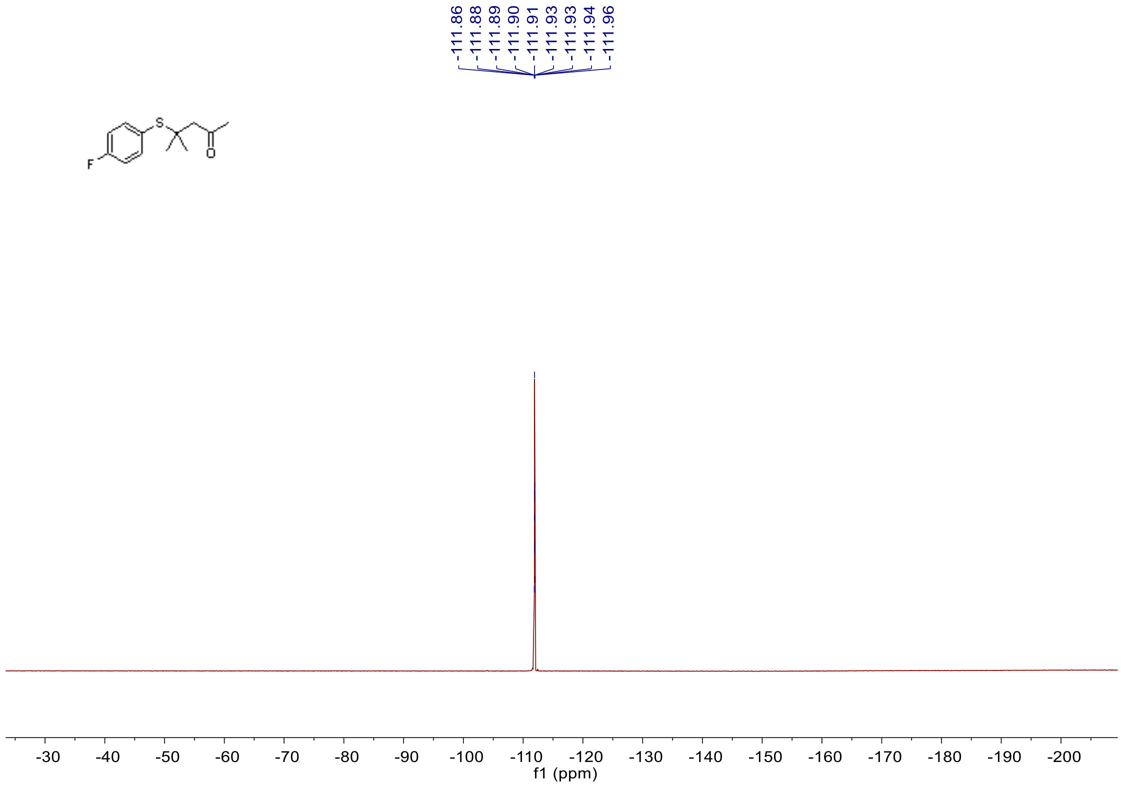

^{1}H NMR of 4-((4-chlorophenyl)thio)-4-methylpentan-2-one (**4h**)

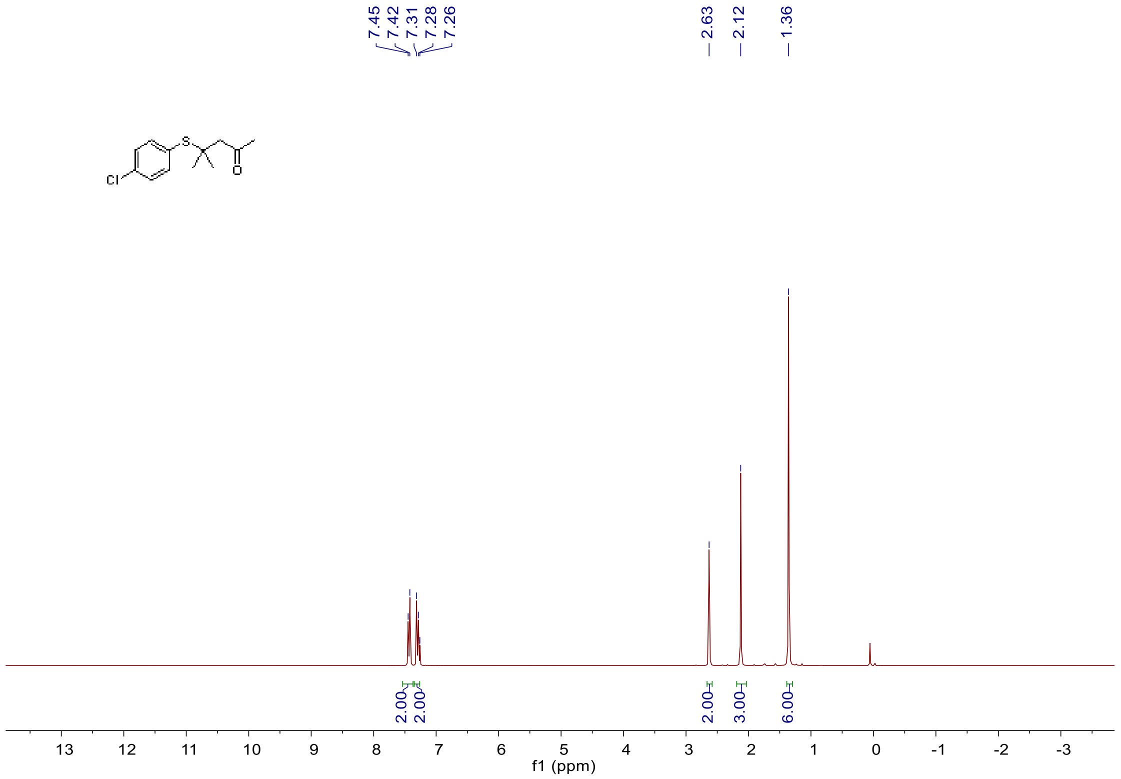

^{13}C NMR of 4-((4-chlorophenyl)thio)-4-methylpentan-2-one (**4h**)

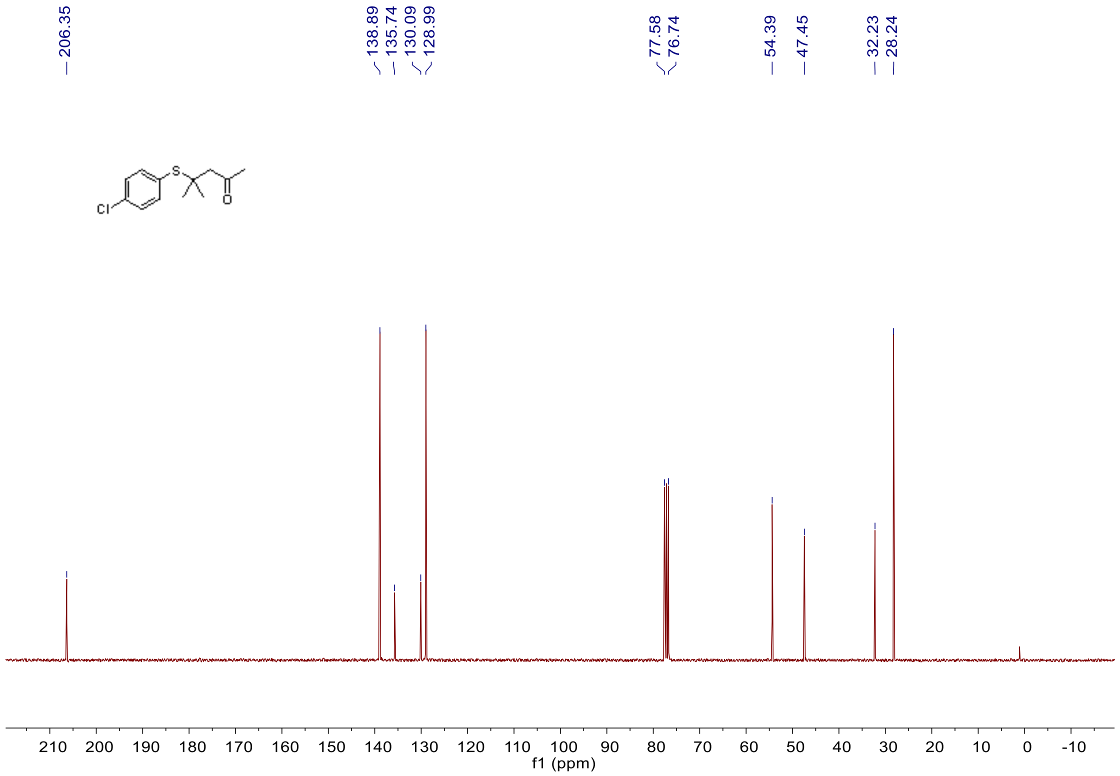

^{1}H NMR of 4-((4-bromophenyl)thio)-4-methylpentan-2-one(**4i**)

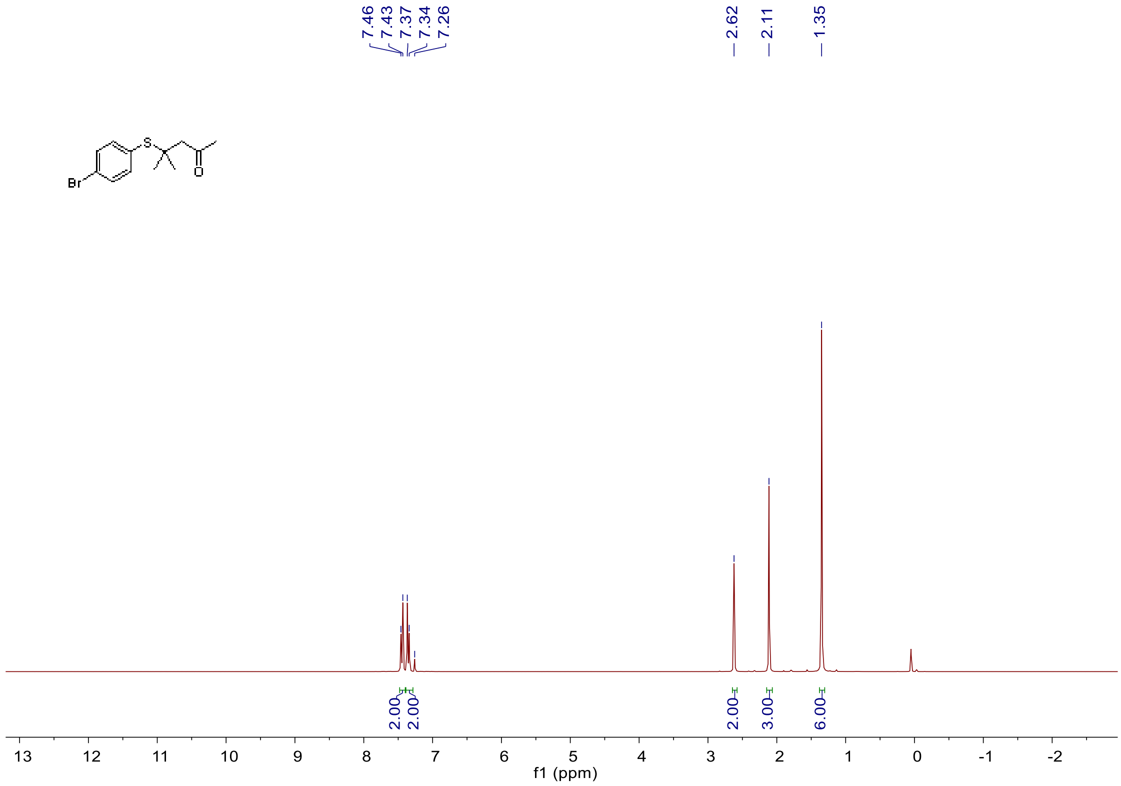

^{13}C NMR of 4-((4-bromophenyl)thio)-4-methylpentan-2-one(**4i**)

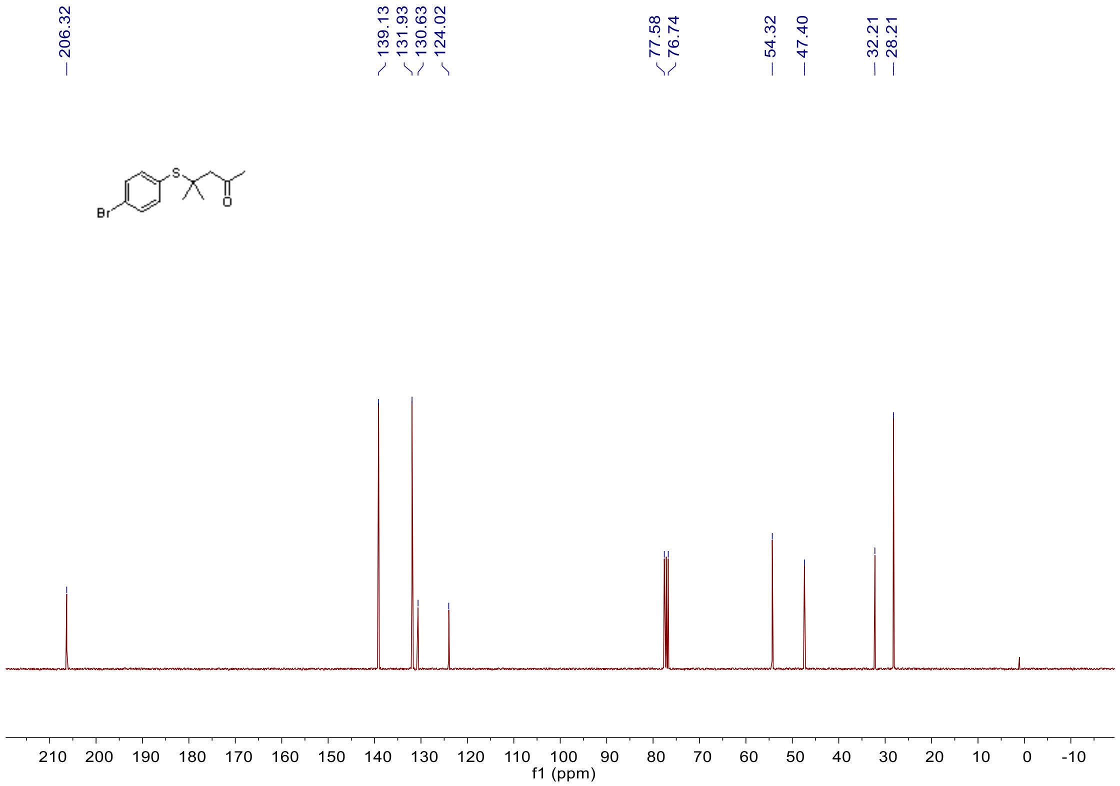

^{1}H NMR of 4-((4-iodophenyl)thio)-4-methylpentan-2-one(**4j**)

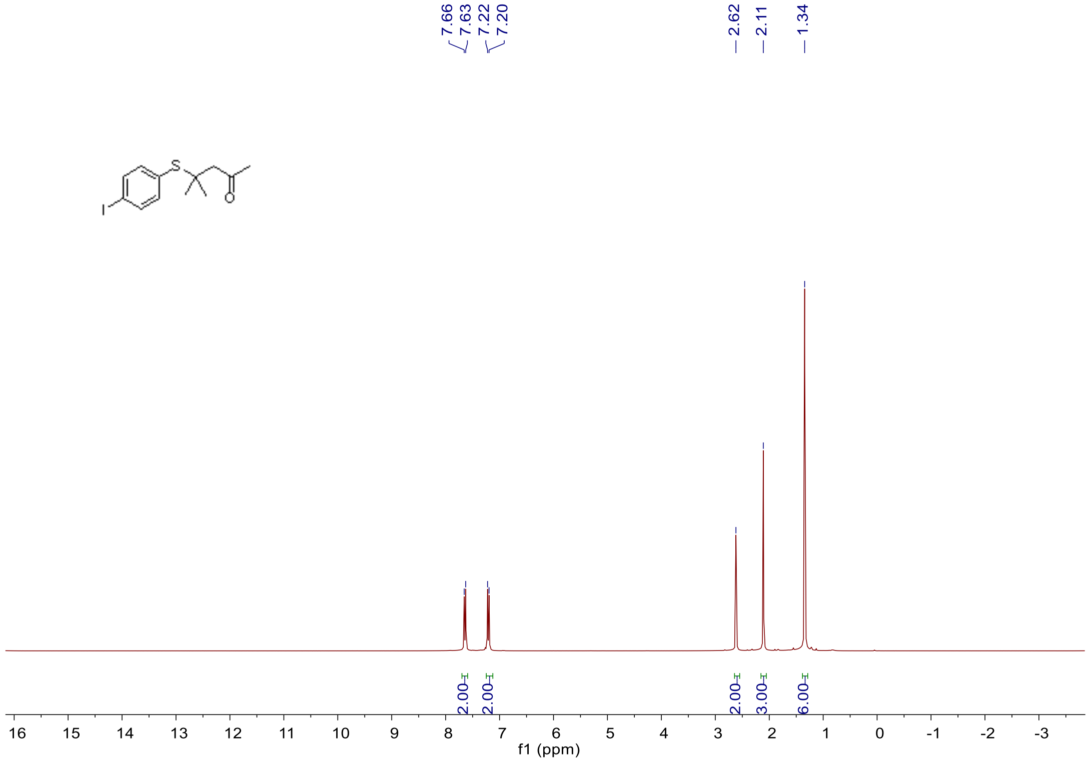

^{13}C NMR 4-((4-iodophenyl)thio)-4-methylpentan-2-one(**4j**)

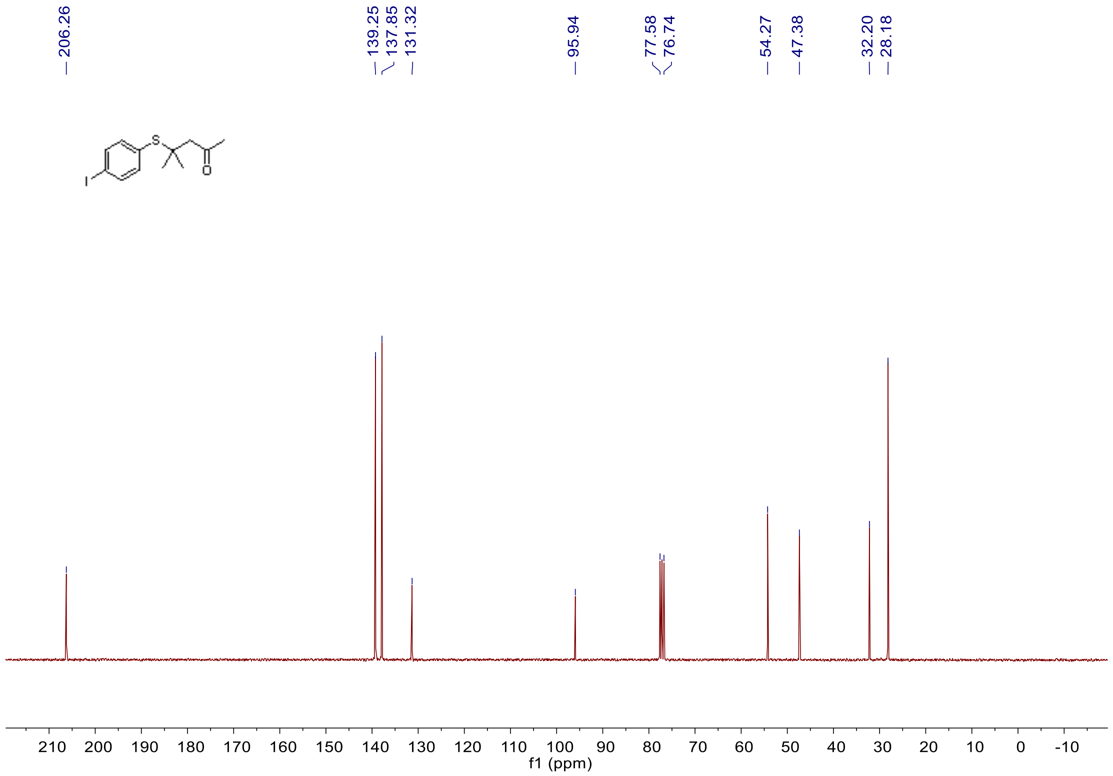

^{1}H NMR of 4-methyl-4-((4-(trifluoromethyl)phenyl)thio)pentan-2-one(**4k**)

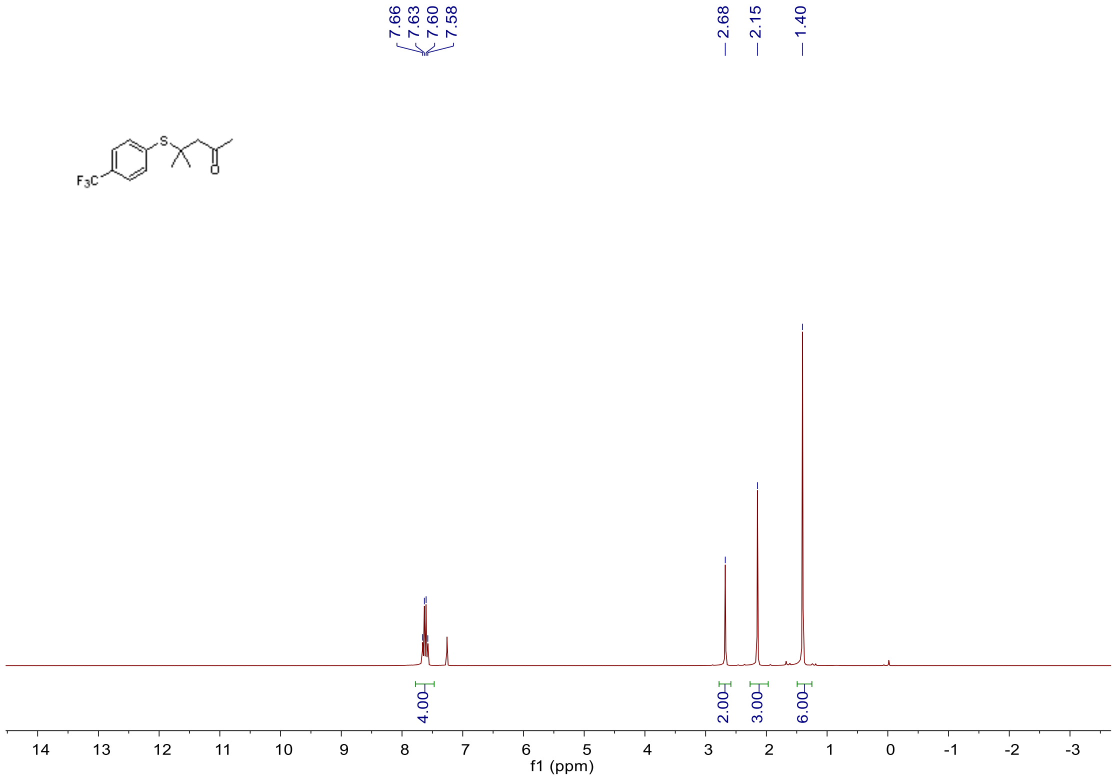

^{13}C NMR of 4-methyl-4-((4-(trifluoromethyl)phenyl)thio)pentan-2-one(**4k**)

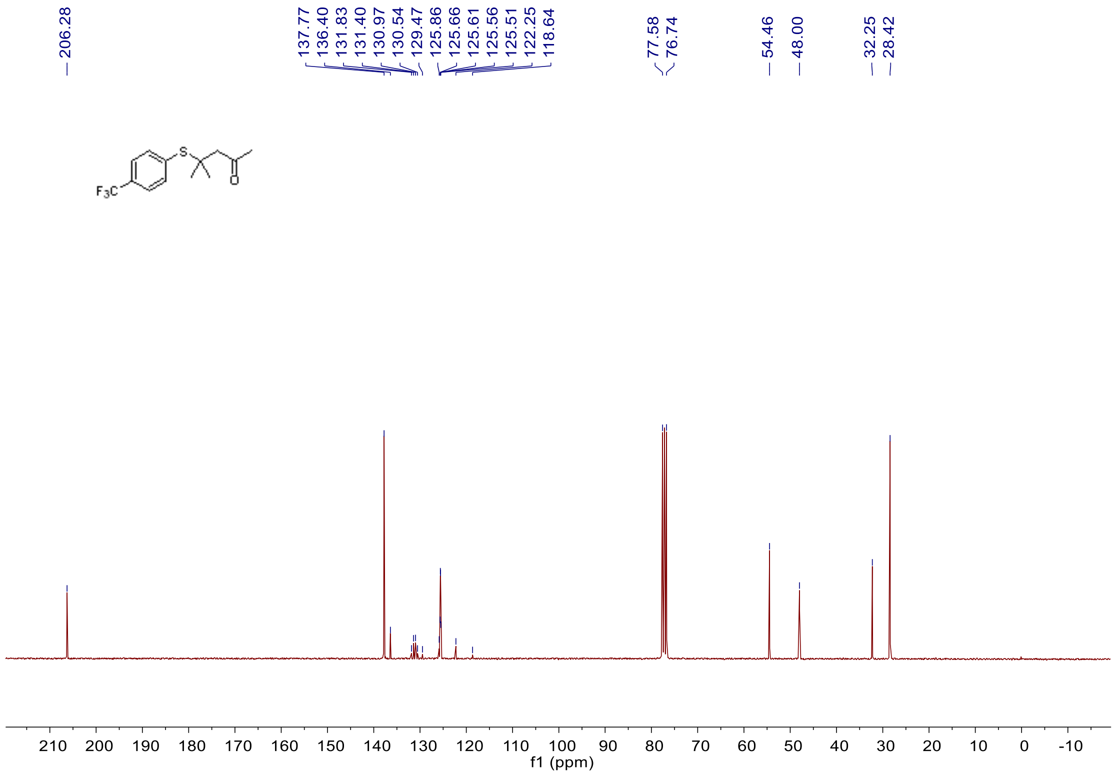

^{19}F NMR of 4-methyl-4-((4-(trifluoromethyl)phenyl)thio)pentan-2-one(**4k**)

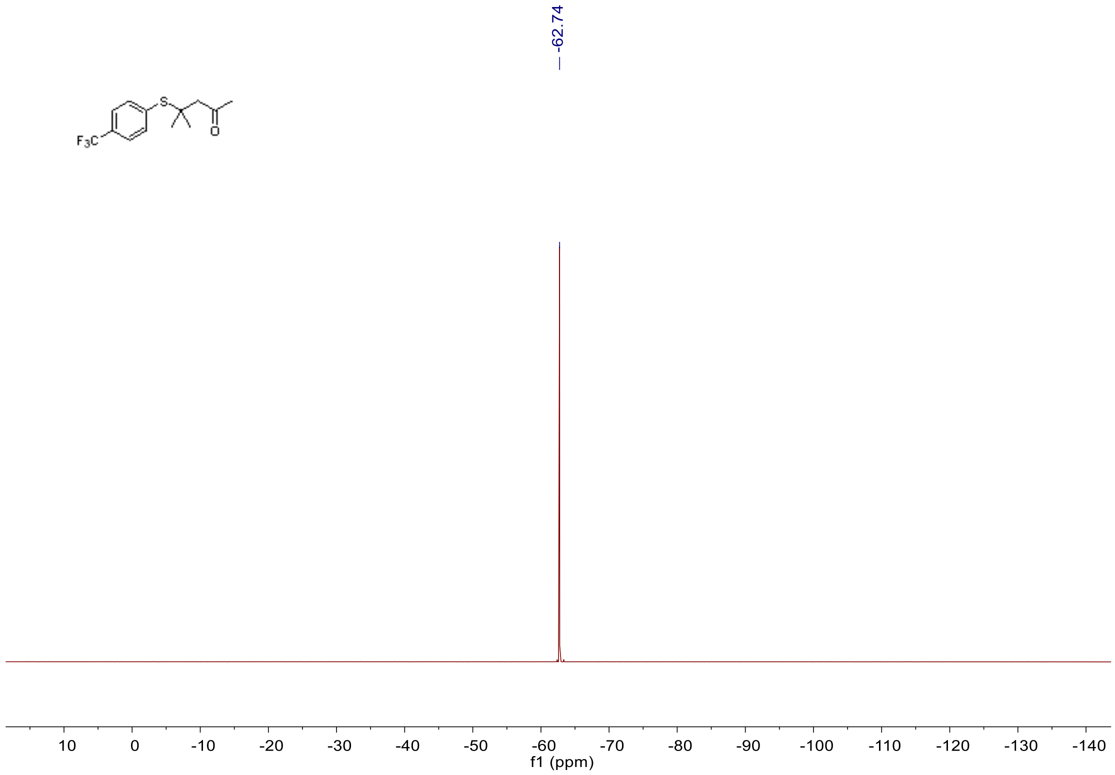

^{1}H NMR of 4-methyl-4-((4-nitrophenyl)thio)pentan-2-one(**4l**)

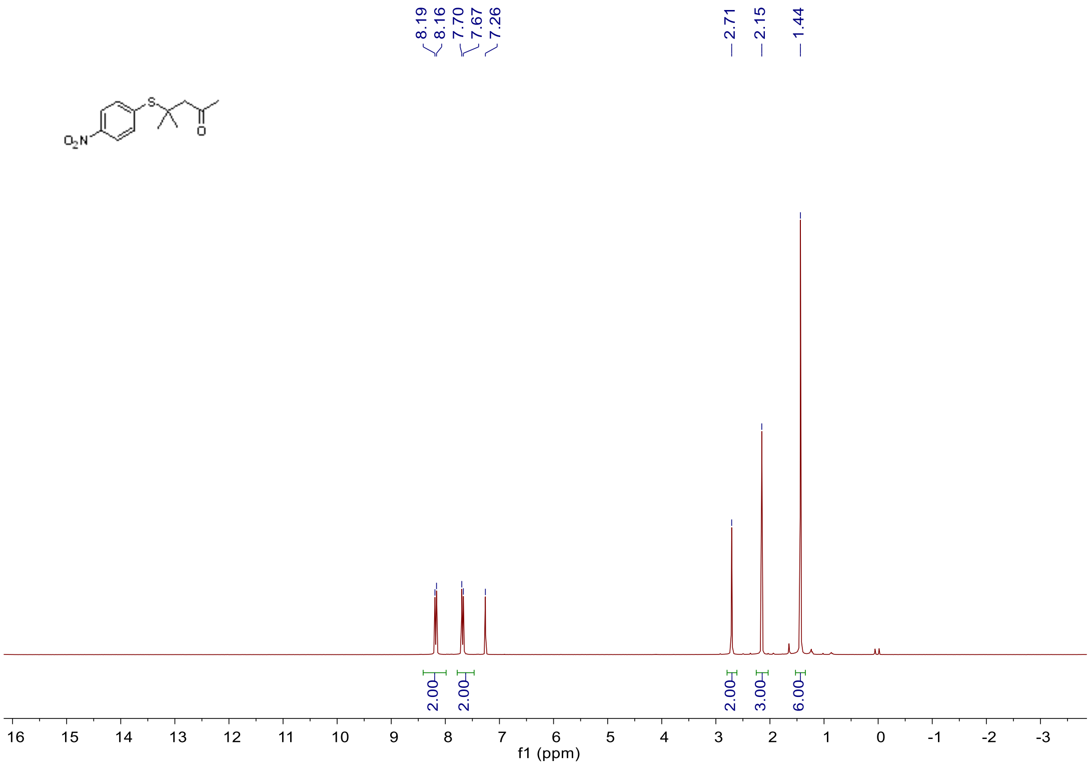

^{13}C NMR of 4-methyl-4-((4-nitrophenyl)thio)pentan-2-one(**4l**)

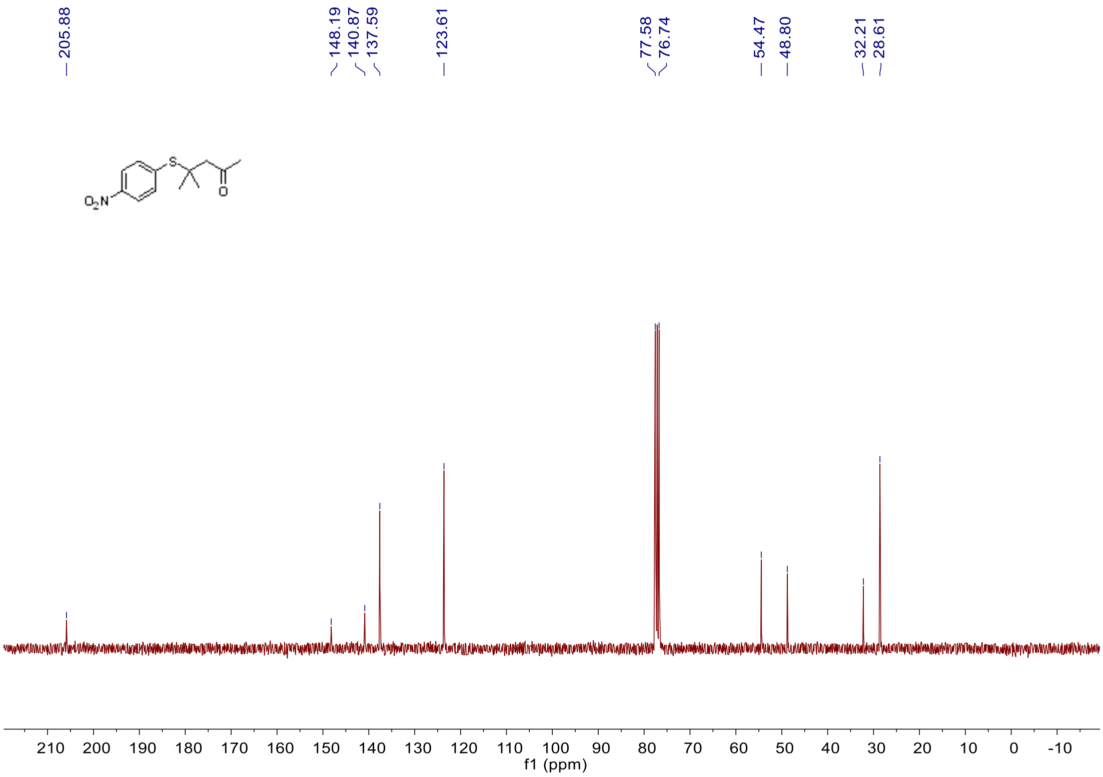

^{1}H NMR of methyl 4-((2-methyl-4-oxopentan-2-yl)thio)benzoate(**4m**)

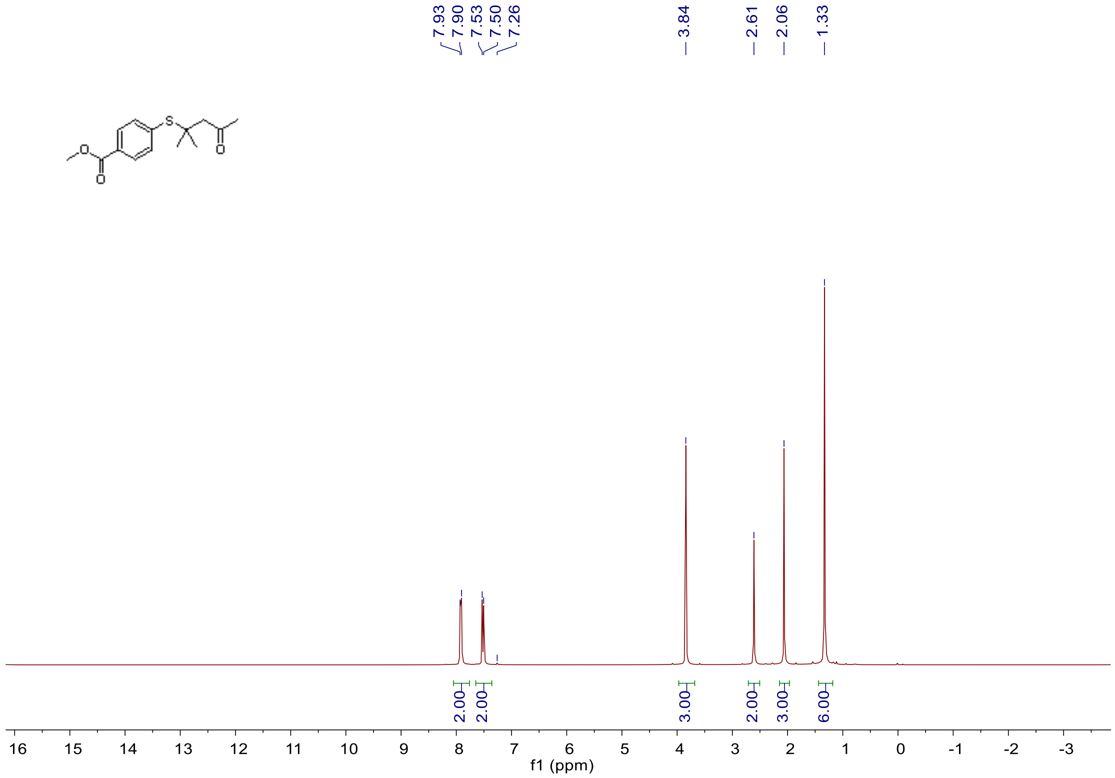

^{13}C NMR of methyl 4-((2-methyl-4-oxopentan-2-yl)thio)benzoate(**4m**)

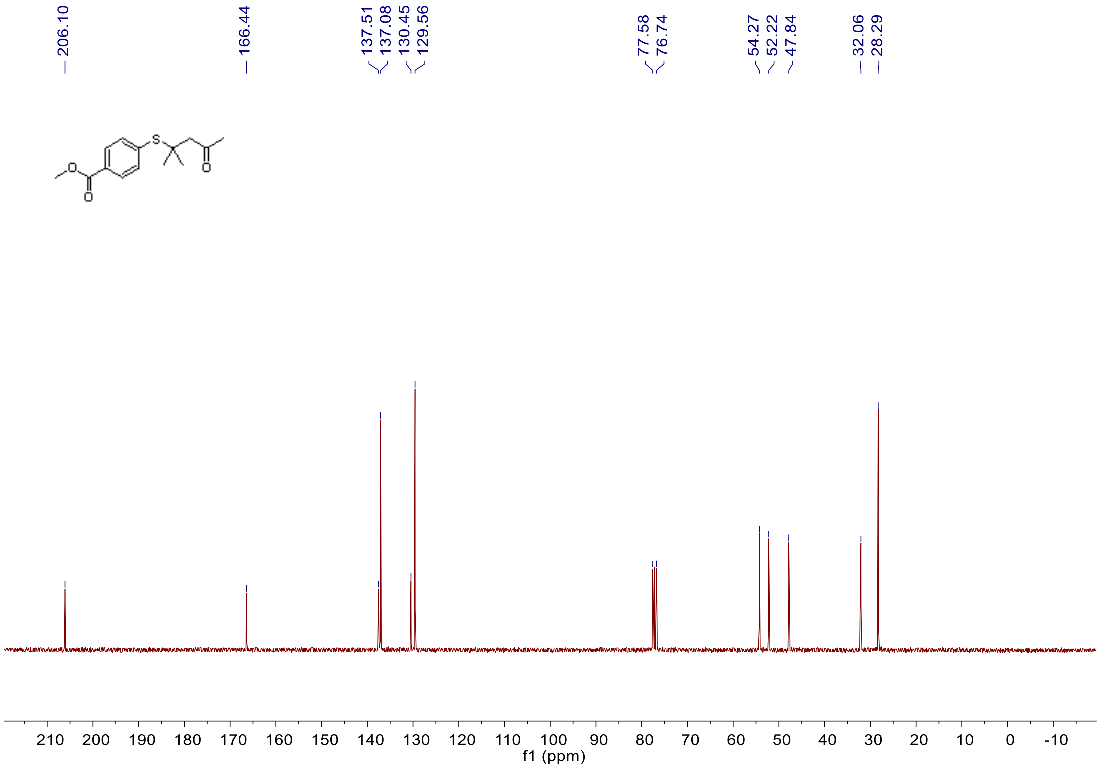

^{1}H NMR of 4-(benzylthio)-4-methylpentan-2-one(**4n**)

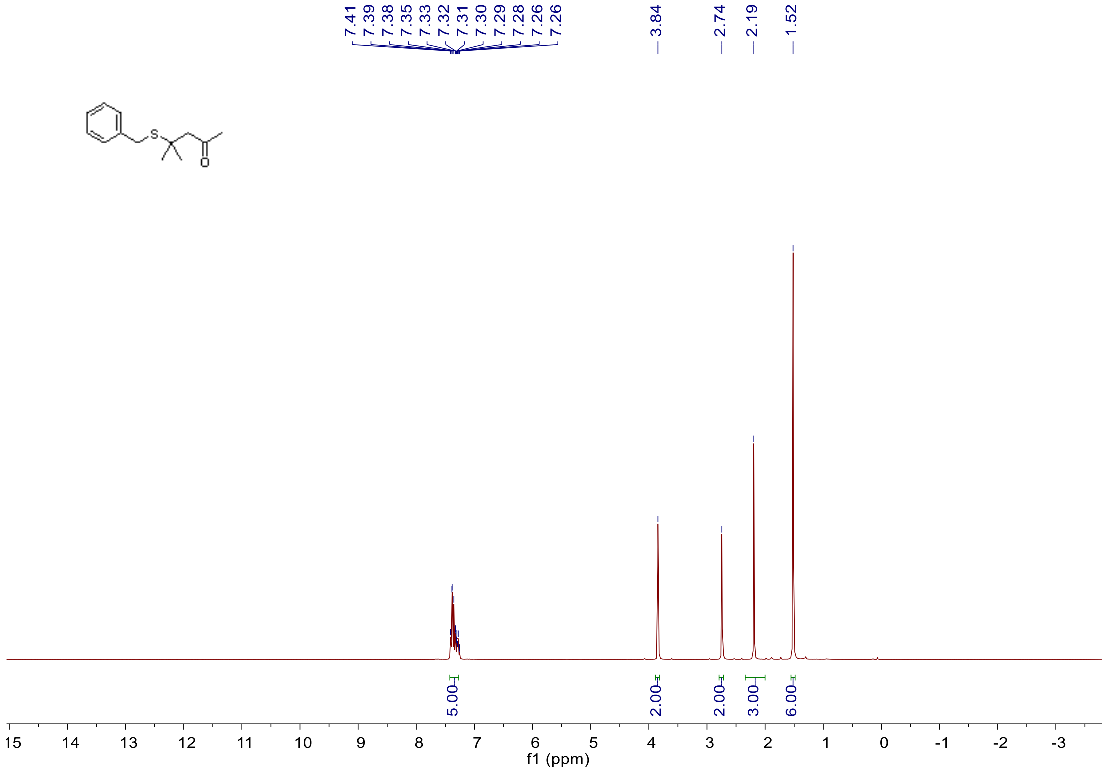

^{13}C NMR of 4-(benzylthio)-4-methylpentan-2-one(**4n**)

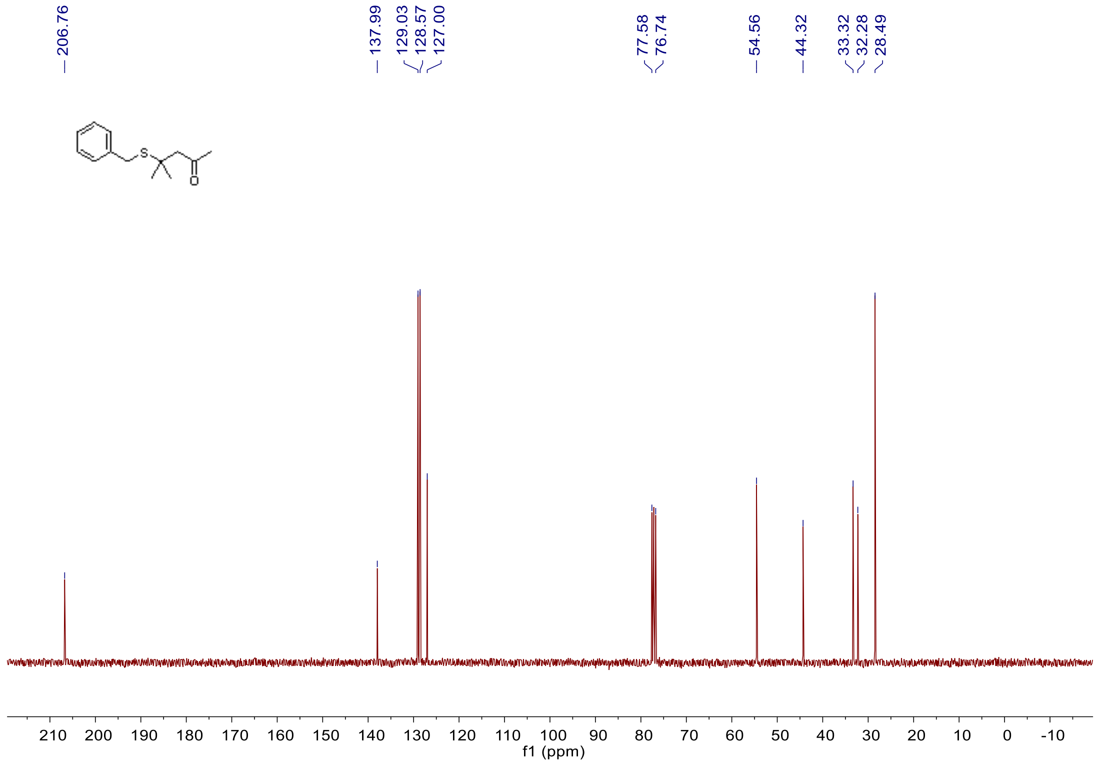

^{1}H NMR of 4-methyl-4-((4-methylbenzyl)thio)pentan-2-one(**4o**)

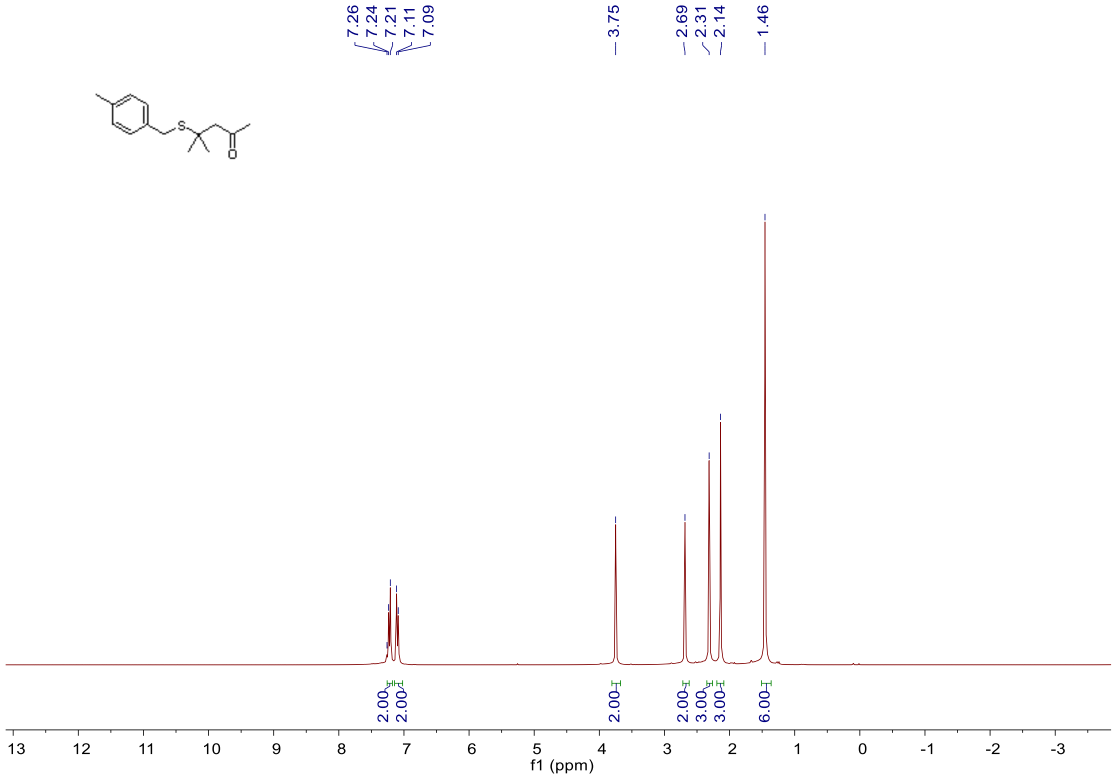

^{13}C NMR of 4-methyl-4-((4-methylbenzyl)thio)pentan-2-one(**4o**)

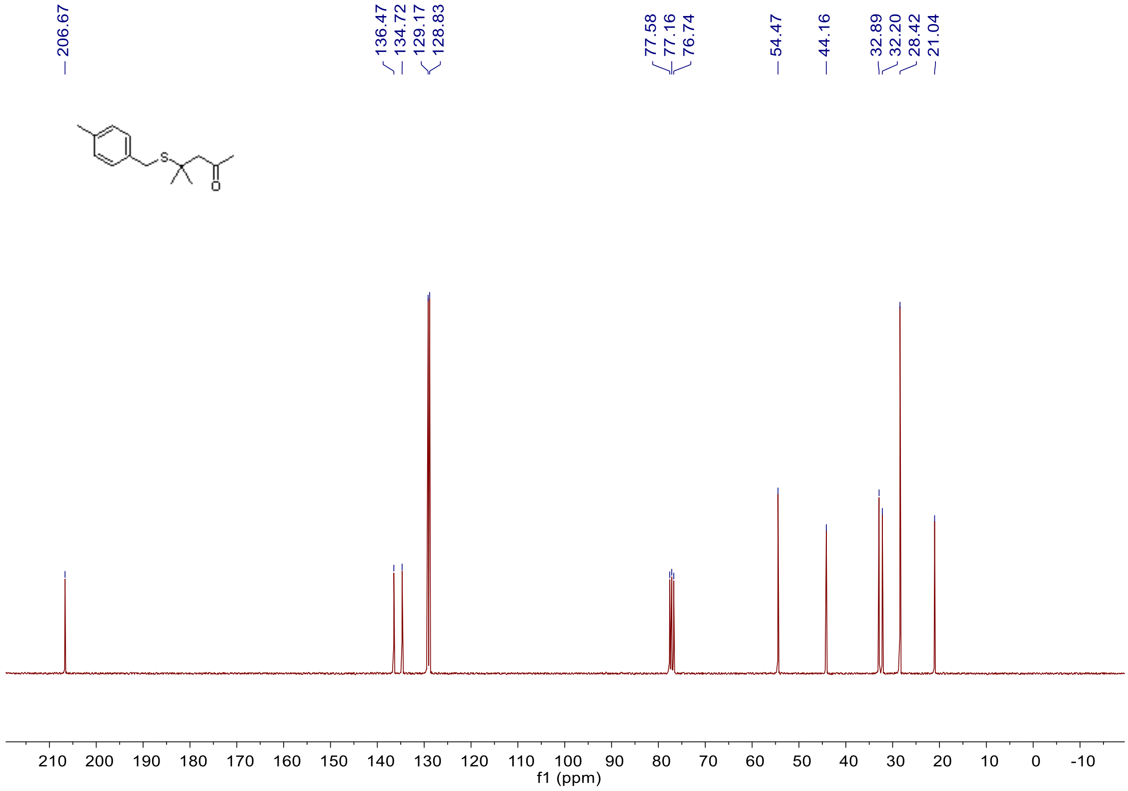

^{1}H NMR of 4-((4-methoxybenzyl)thio)-4-methylpentan-2-one(**4p**)

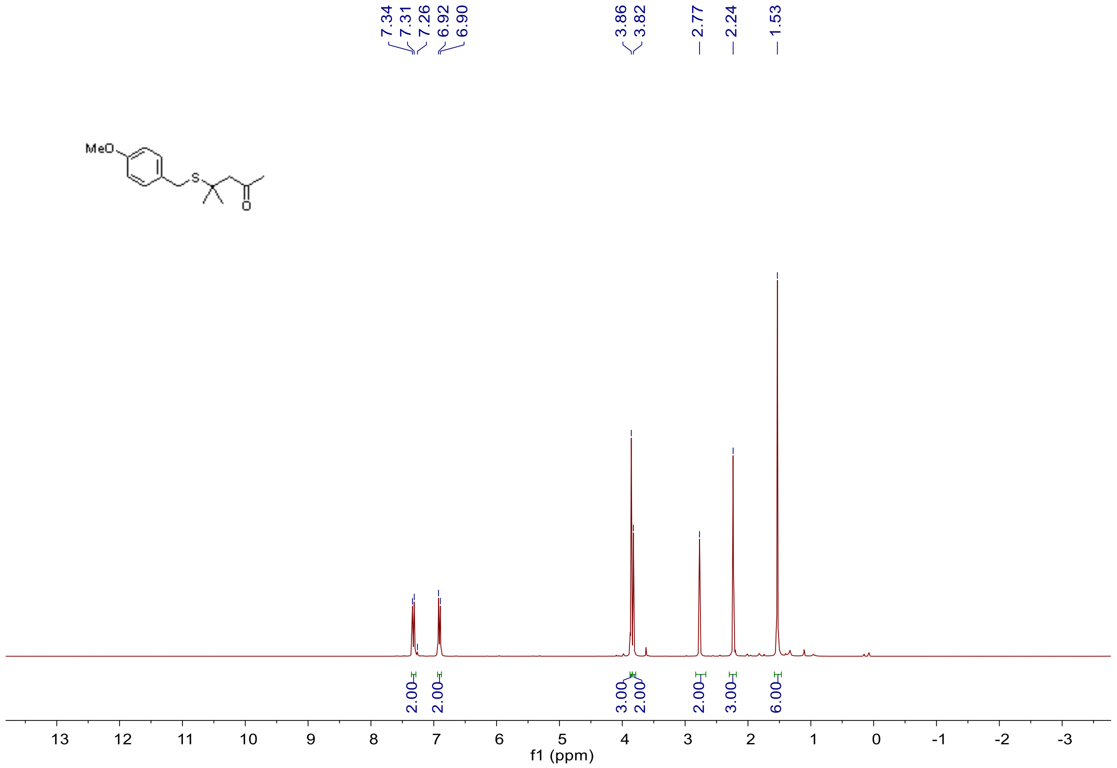

^{13}C NMR of 4-((4-methoxybenzyl)thio)-4-methylpentan-2-one(**4p**)

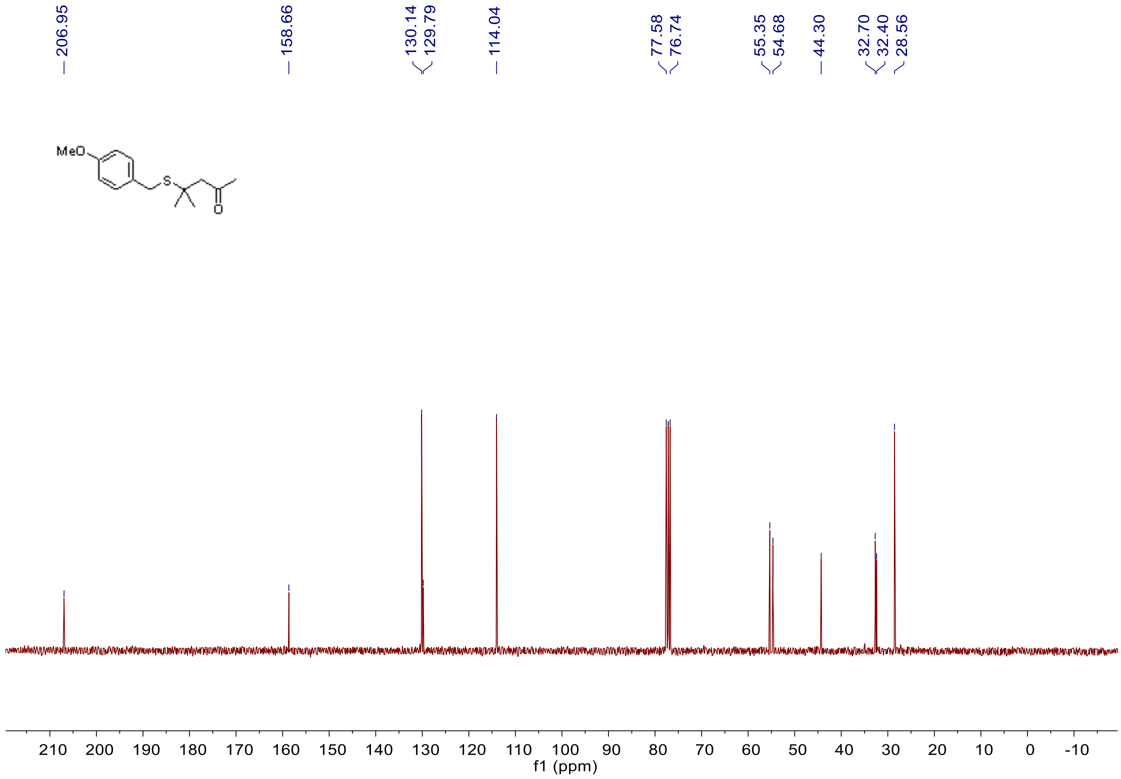

^{1}H NMR of 4-((4-chlorobenzyl)thio)-4-methylpentan-2-one(**4q**)

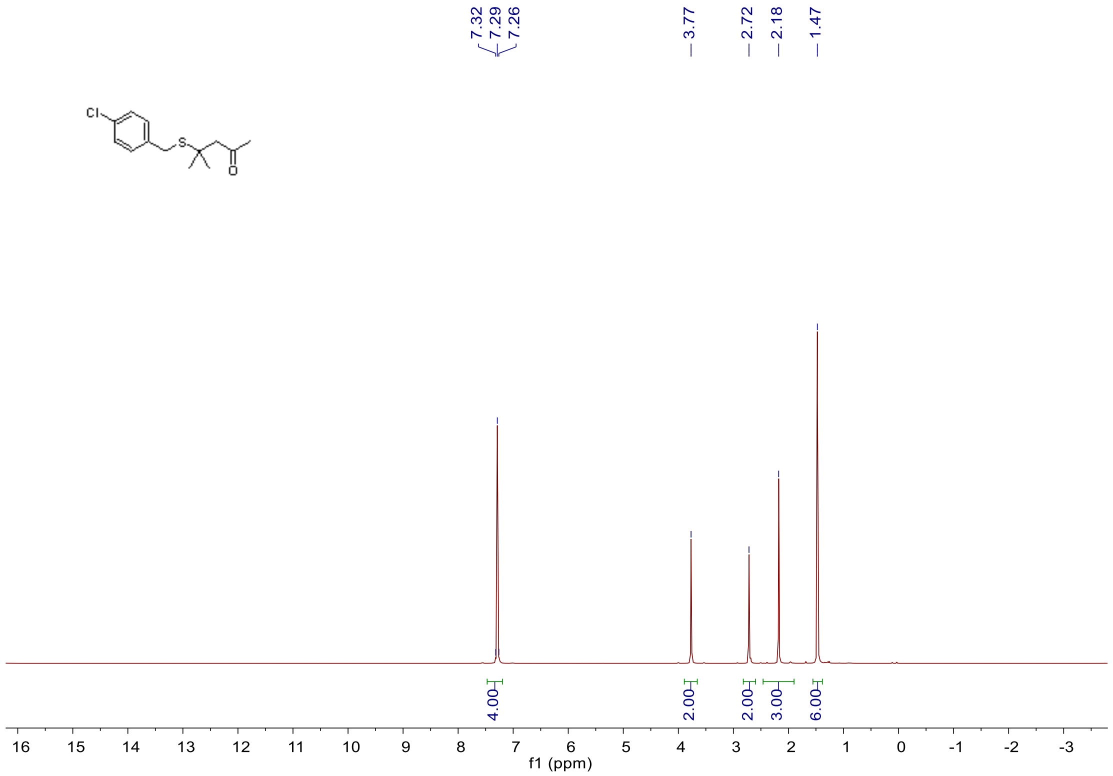

^{13}C NMR of 4-((4-chlorobenzyl)thio)-4-methylpentan-2-one(**4q**)

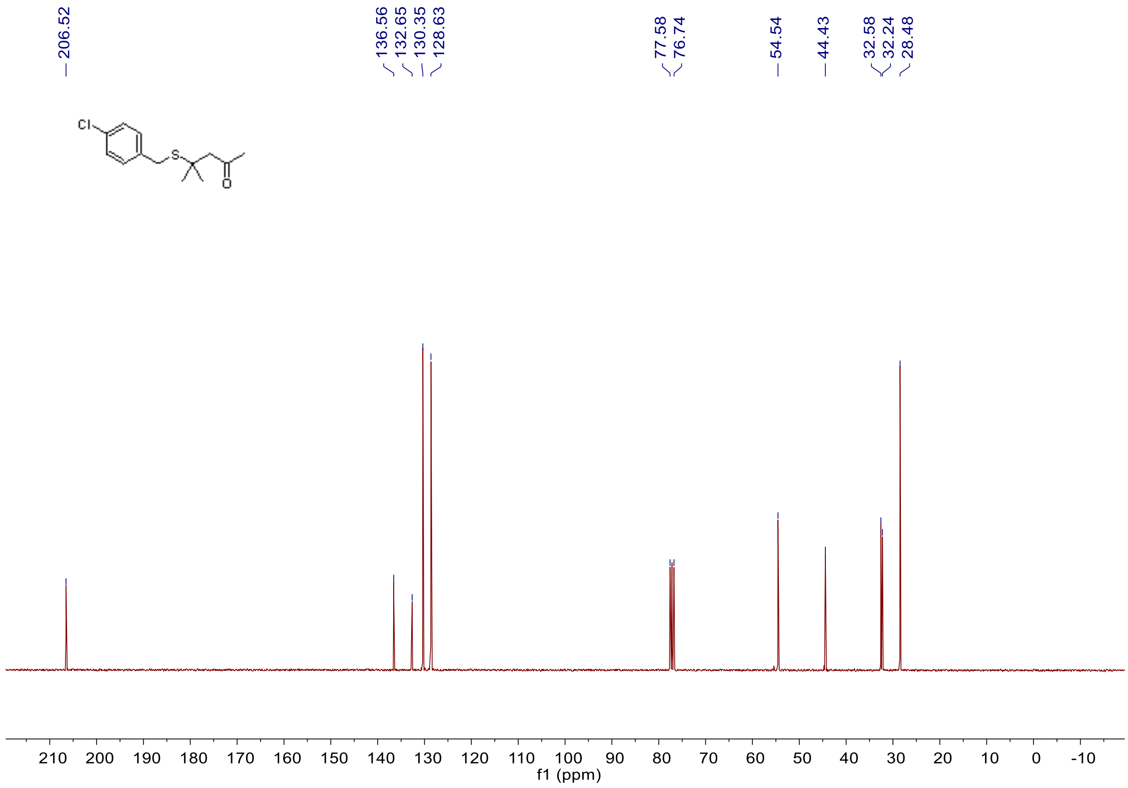

^{1}H NMR of 4-(butylthio)-4-methylpentan-2-one(**4r**)

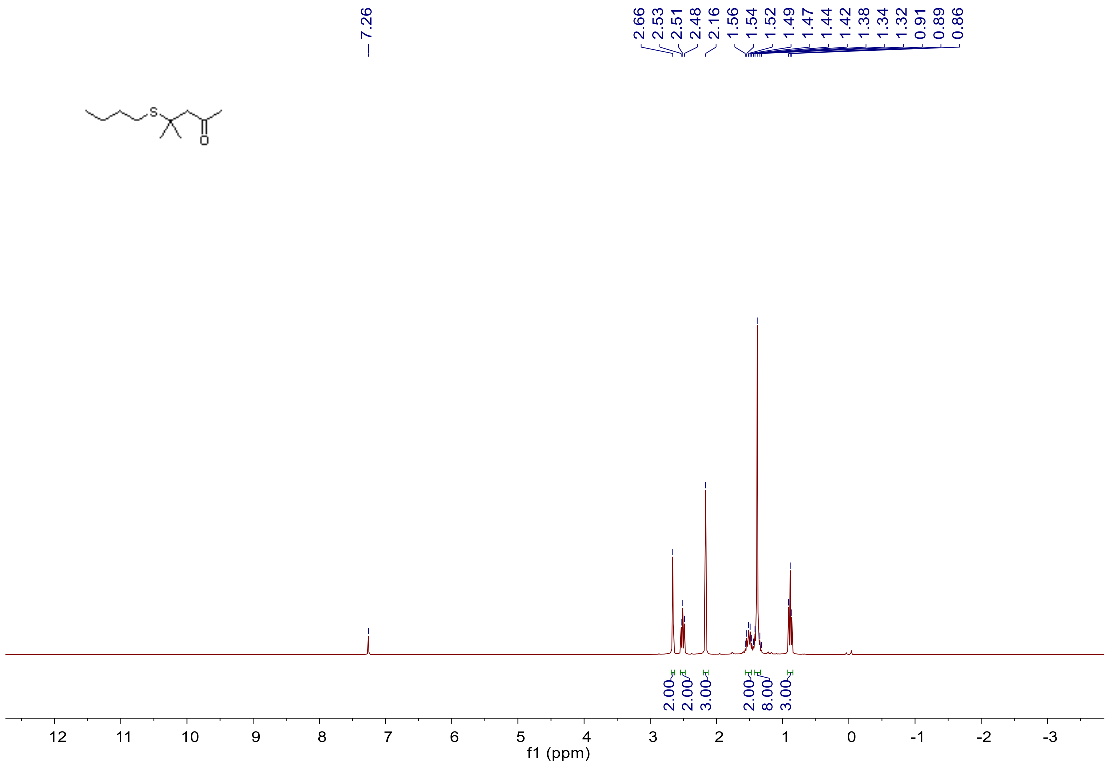

^{13}C NMR of 4-(butylthio)-4-methylpentan-2-one(**4r**)

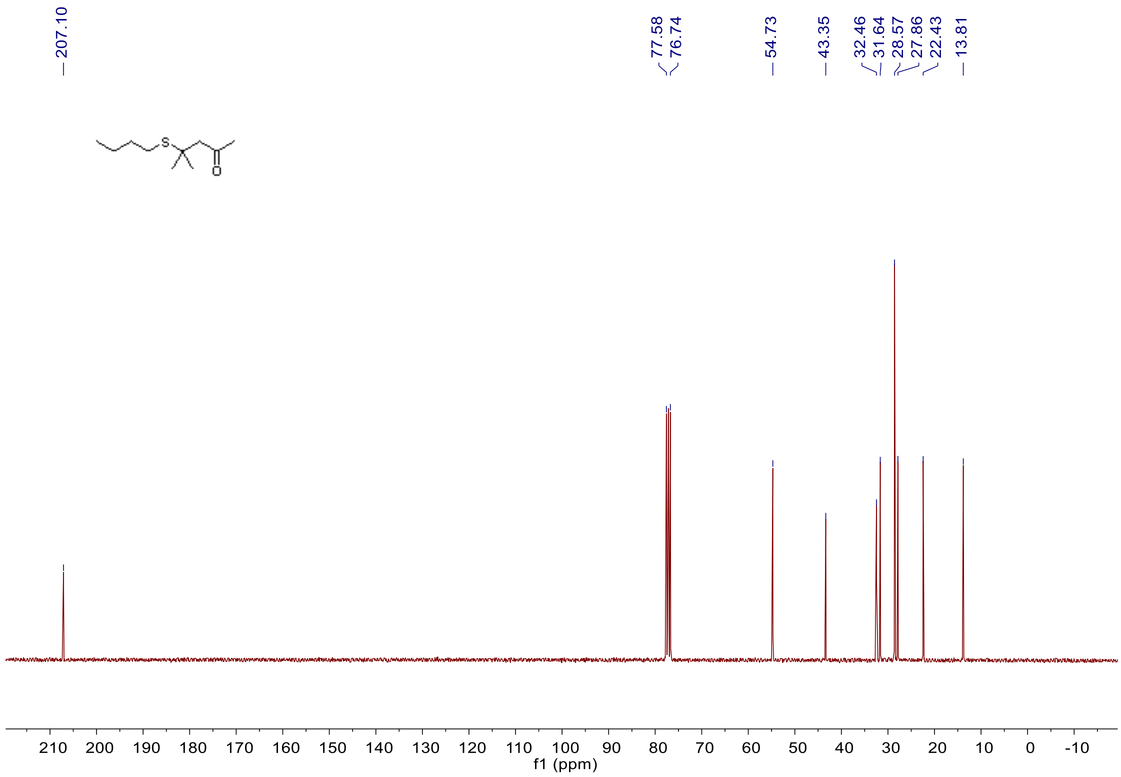

^{1}H NMR of 4-(cyclohexylthio)-4-methylpentan-2-one(**4s**)

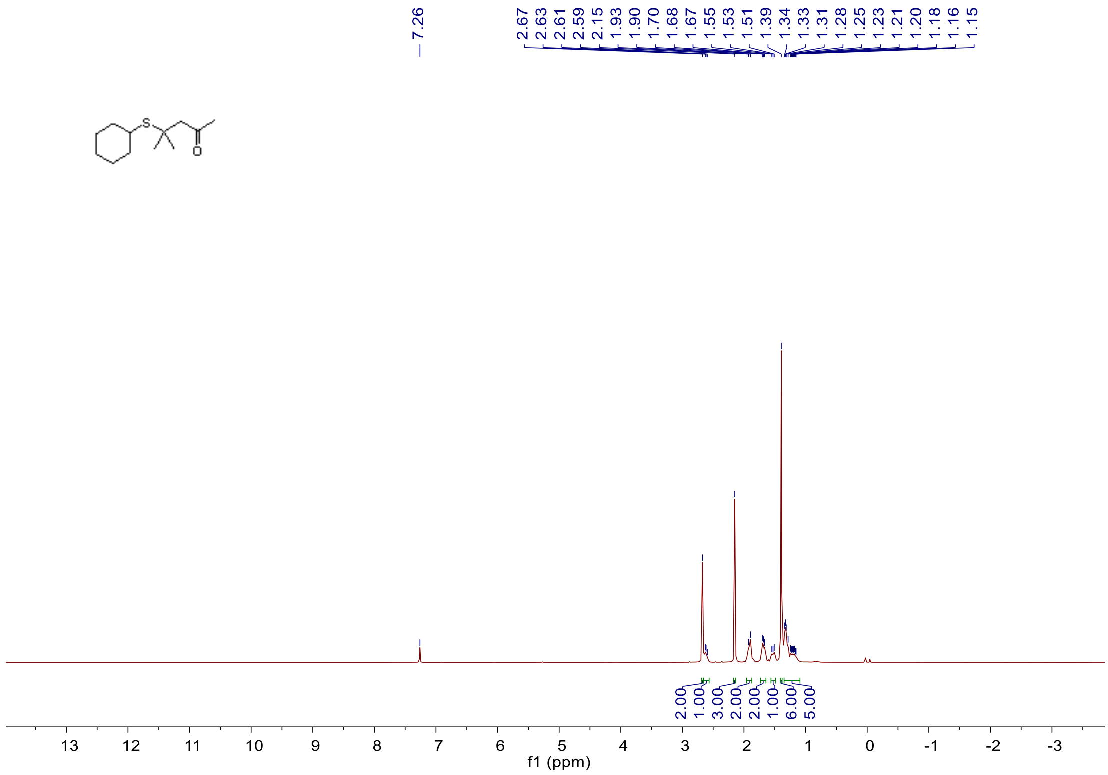

^{13}C NMR of 4-(cyclohexylthio)-4-methylpentan-2-one(**4s**)

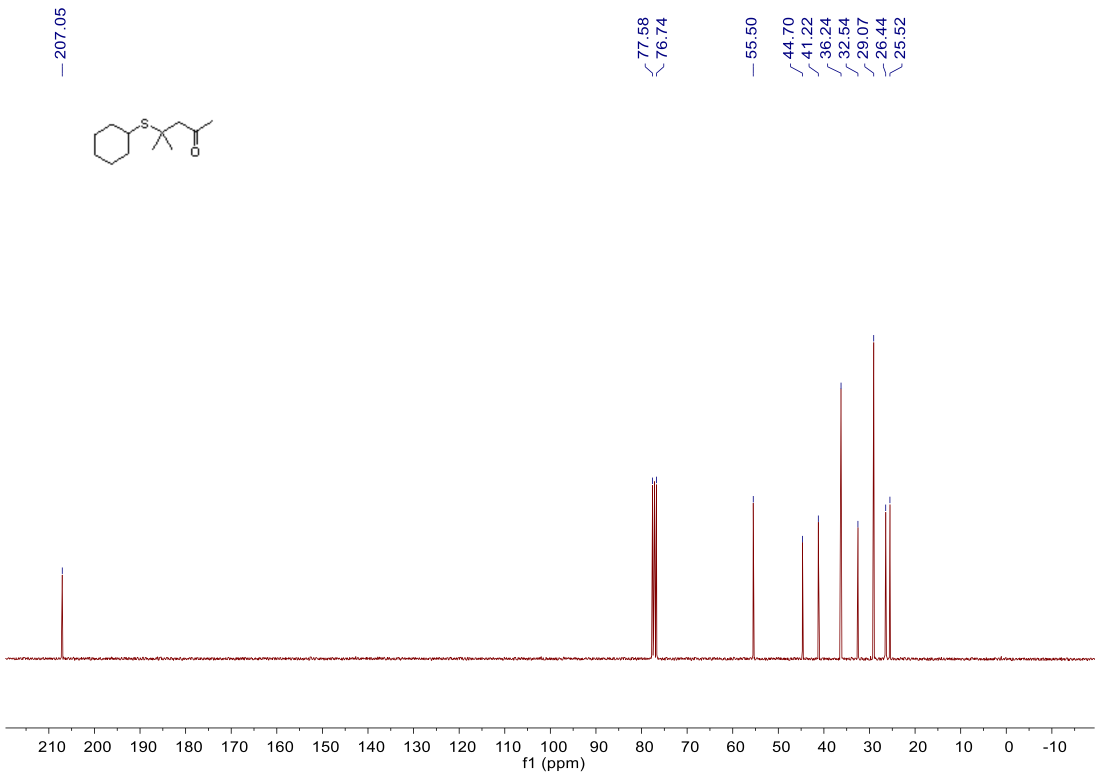

^{1}H NMR of 4-methyl-4-(naphthalen-2-ylthio)pentan-2-one(**4t**)

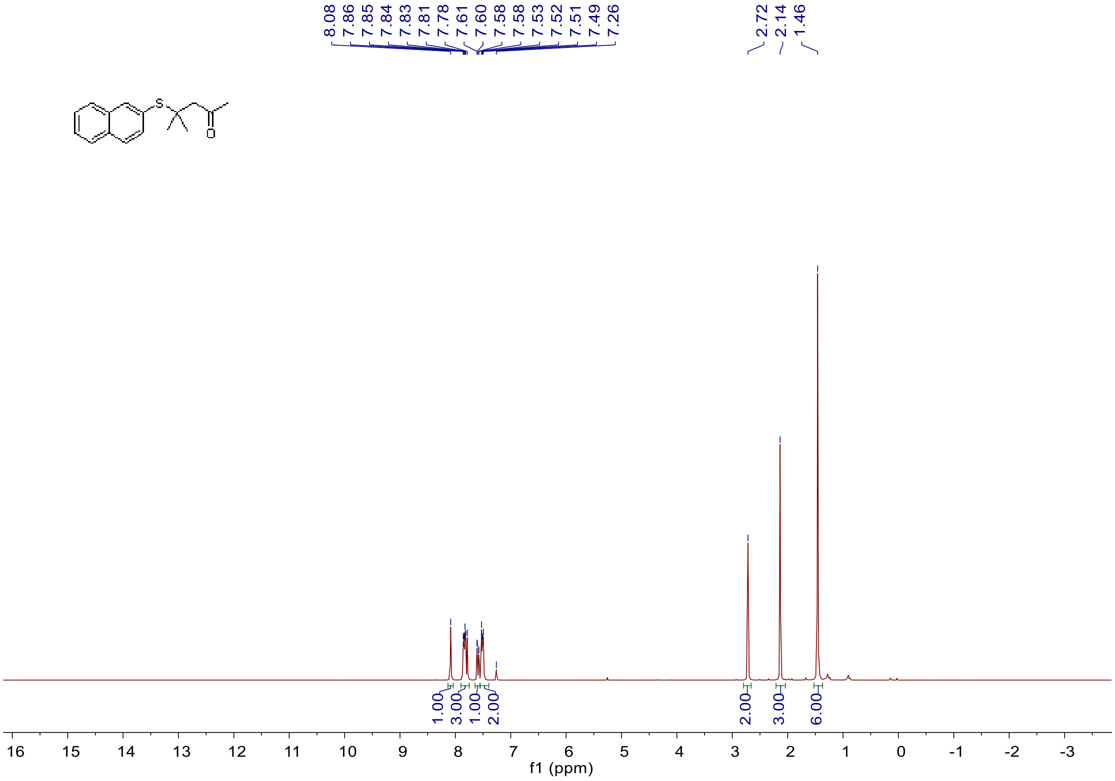

^{13}C NMR of 4-methyl-4-(naphthalen-2-ylthio)pentan-2-one(**4t**)

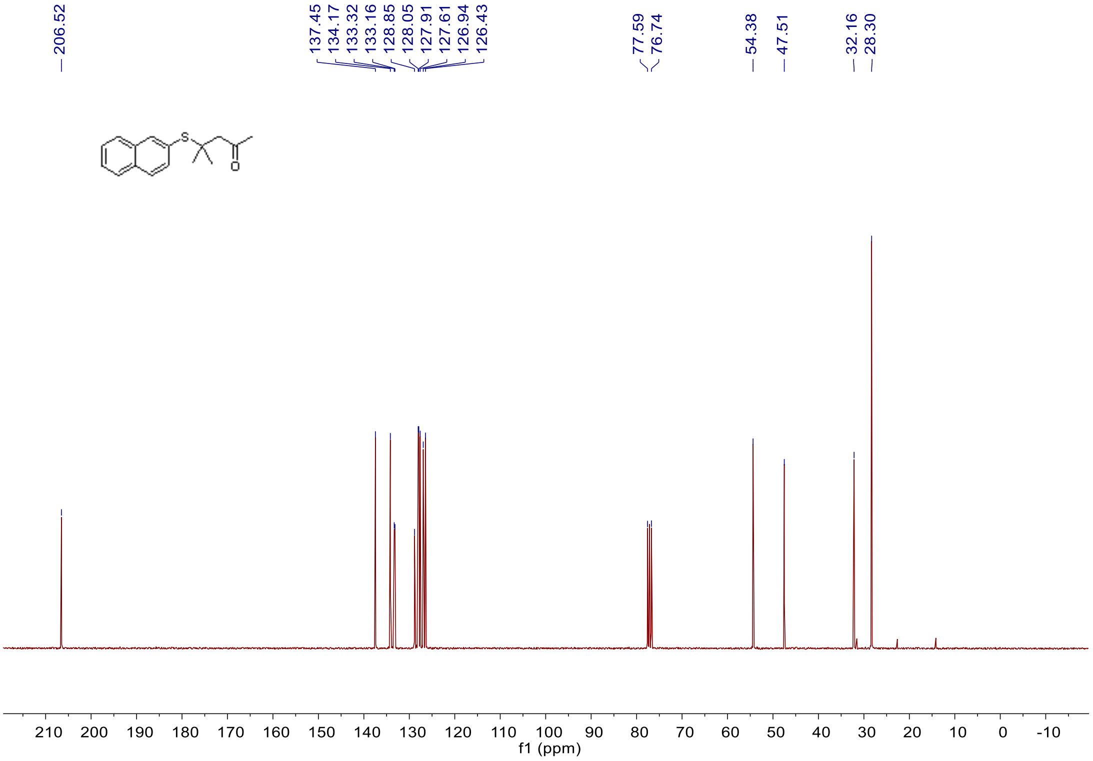

^{1}H NMR of 4-methyl-4-(thiophen-2-ylthio)pentan-2-one(**4u**)

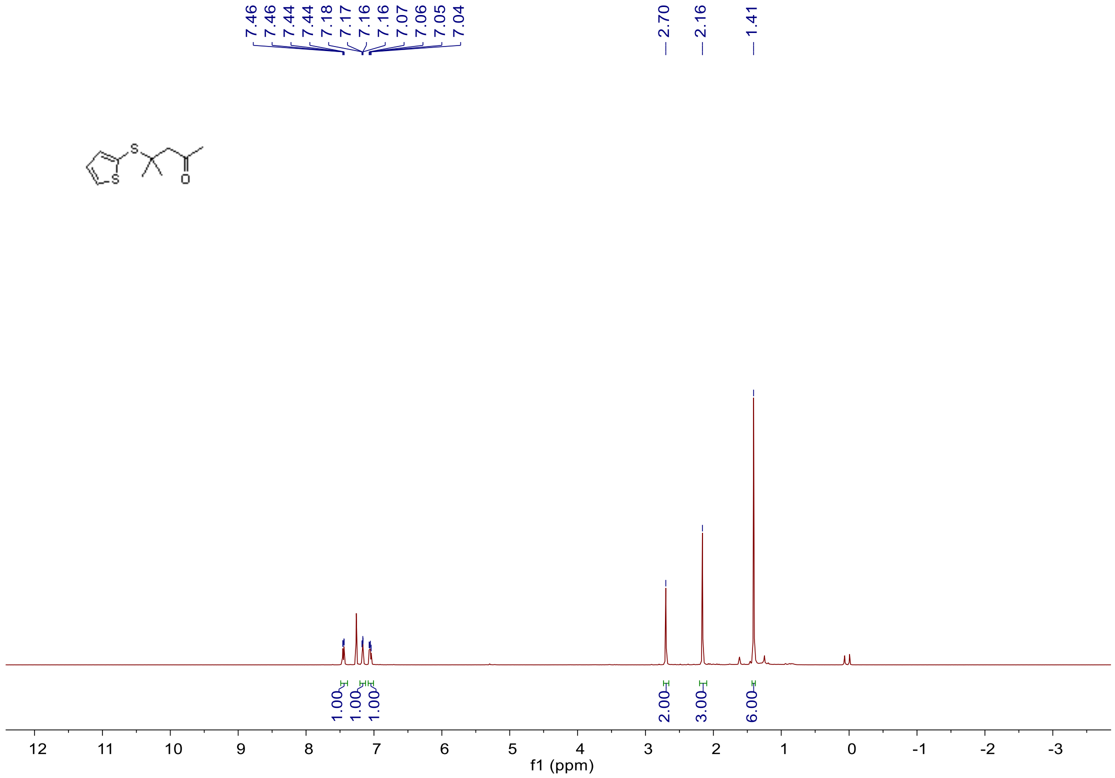

^{13}C NMR of 4-methyl-4-(thiophen-2-ylthio)pentan-2-one(**4u**)

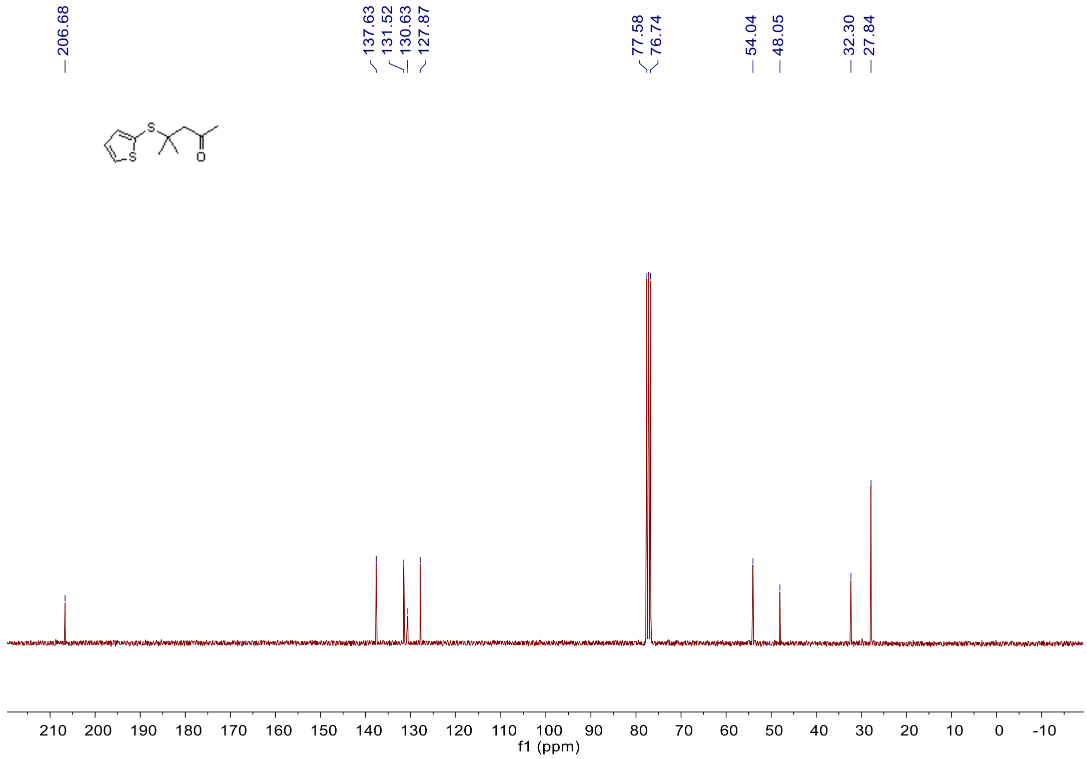

Supplement: Supplementary file 1 [file molecules-29-04785-s001.zip › molecules-3213538-supplementary.pdf]
